# Interspecific hybridization in *Brassica* species leads to changes in agronomic traits through the regulation of gene expression by chromatin accessibility and DNA methylation

**DOI:** 10.1093/gigascience/giaf029

**Published:** 2025-04-22

**Authors:** Chengtao Quan, Qin Zhang, Xiaoni Zhang, Kexin Chai, Guoting Cheng, Chaozhi Ma, Cheng Dai

**Affiliations:** National Key Laboratory of Crop Genetic Improvement, Huazhong Agricultural University, Wuhan 430070, China; Hubei Hongshan Laboratory, Wuhan 430070, China; National Key Laboratory of Crop Genetic Improvement, Huazhong Agricultural University, Wuhan 430070, China; Hubei Hongshan Laboratory, Wuhan 430070, China; National Key Laboratory of Crop Genetic Improvement, Huazhong Agricultural University, Wuhan 430070, China; Hubei Hongshan Laboratory, Wuhan 430070, China; National Key Laboratory of Crop Genetic Improvement, Huazhong Agricultural University, Wuhan 430070, China; Hubei Hongshan Laboratory, Wuhan 430070, China; College of Informatics, Huazhong Agricultural University, Wuhan 430070, China; National Key Laboratory of Crop Genetic Improvement, Huazhong Agricultural University, Wuhan 430070, China; Hubei Hongshan Laboratory, Wuhan 430070, China; National Key Laboratory of Crop Genetic Improvement, Huazhong Agricultural University, Wuhan 430070, China; Hubei Hongshan Laboratory, Wuhan 430070, China

**Keywords:** chromatin accessibility, hybridization, DNA methylation, transposable elements, transgressive

## Abstract

Interspecific hybridization is a common method in plant breeding to combine traits from different species, resulting in allopolyploidization and significant genetic and epigenetic changes. However, our understanding of genome-wide chromatin and gene expression dynamics during allopolyploidization remains limited. This study generated two *Brassica* allotriploid hybrids via interspecific hybridization. We observed that accessible chromatin regions (ACRs) and DNA methylation interact to regulates gene expression after interspecific hybridization, ultimately influencing the agronomic traits of the hybrids. In total, 234,649 ACRs were identified in the parental lines and hybrids; the hybridization process induces changes in the distribution and abundance of their accessible chromatin regions, particularly in gene regions and their proximity. Genes associated with proximal ACRs were more highly expressed than those associated with distal and genic ACRs. More than half of novel ACRs drove transgressive gene expression in the hybrids, and the transgressive upregulated genes showed significant enrichment in metal ion binding, especially magnesium ion, calcium ion, and potassium ion binding. We also identified *Bna.bZIP11* in the single-parent activation ACR, which binds to *BnaA06.UF3GT* to promote anthocyanin accumulation in F_1_ hybrids. DNA methylation plays a role in repressing gene expression, and unmethylated ACRs are more transcriptionally active. Additionally, the A-subgenome ACRs were associated with genome dosage rather than DNA methylation. The interplay among DNA methylation, transposable elements, and sRNA contributes to the dynamic landscape of ACRs during interspecific hybridization, resulting in distinct gene expression patterns on the genome.

## Background

Interspecific hybridization is an essential tool in plant breeding and genetic improvement. It allows the incorporation of desirable traits from different species into a single organism, improving crop quality and productivity [1–4]. This process has revolutionized crop breeding and contributed significantly to the global food supply [5, 6]. A major advantage of interspecific hybridization is the generation of hybrids with superior vigor compared with their parents. This vigor arises from interactions between alleles at multiple loci, epistatic interactions, and possibly complementation of deleterious mutations [7]. Emerging evidence suggests that epigenetic factors are crucial for hybrid vigor [2, 8]. However, the genetic mechanisms underlying interspecific hybridization are complex and poorly understood.

Accessible chromatin regions (ACRs) are typically nucleosome-free or loosely bound to nucleosomes, making them more susceptible to binding by regulatory proteins that affect gene expression. In plant genomes, chromatin's open state directly influences gene expression regulation during essential biological processes such as cell differentiation, growth, and development [9–11]. Pinpointing ACRs is critical to understanding *cis*-regulatory elements (CREs) in the genome, which form the intricate transcriptional regulatory networks that control gene expression [12]. For instance, auxin has been shown to quickly rewire the totipotency network involved in somatic embryogenesis in *Arabidopsis* by altering chromatin accessibility [13]. Further investigation shows that the B3-type transcription factor LEC2 promotes the formation of somatic embryos by directly activating the early embryonic patterning genes *WOX2* and *WOX3* [13]. In *Arabidopsis*, the long-day condition induces a greater number of ACRs in the leaf epidermis and vascular companion cell; additionally, compared with the leaf epidermis cell, more ACRs were identified in the vascular companion cells, which are situated further away from the gene region [14]. ACRs at different locations in the genome can be categorized as genic, proximal, and distal [10]. Genic ACRs are usually located within genes, such as exons and introns, which affect the conduct of transcription and the processing of mRNA [15]. Therefore, the maintenance and monitoring of genic ACRs is essential to maintain the integrity and stability of the genome [16]. Proximal ACRs are typically located near critical promoter regions that bind transcription factors and other regulatory proteins, directly influencing the expression of nearby genes [12, 13]. Several binding sites for flowering-related transcription factors were identified in proximal ACRs induced by long-day in *Arabidopsis*, and *trehalose phosphatase/synthase 9* (*TPS9*) was identified as a flowering activator [14]. Distal ACRs contain long-range-acting regulatory elements, such as enhancers or silencers, essential for regulating complex gene expression patterns [14]. Despite their long physical distance from target genes, they can interact with the promoter regions of genes through multiple mechanisms (e.g., chromatin looping and folding) to influence gene expression [17, 18]. For instance, distal ACRs can be modified by H3K56 acetylation (H3K56ac), and may function as enhancers, but when modified by H3K27 trimethylation (H3K27me3) may act as repressors [19]. Changes in these ACRs can result in cell-specific alterations in expression patterns and biological functions [13, 14, 19]. In summary, ACRs provide valuable insights into how the physical structure of chromatin affects gene expression. Thus, unraveling these ACRs and their association with gene expression profiles may help to elucidate the complex regulatory network behind hybrid vigor.

DNA methylation is a vital process of epigenetic modification in which methyl groups are added to the DNA molecule. It is critical in regulating gene expression without affecting the genetic sequence [20, 21]. Most DNA methylation patterns in plants are generally stable, but DNA methylation in CHG and CHH contexts can be altered following interspecific hybridization [22]. Dynamic DNA methylation variation was observed during the development of hybrid rice, with many of the differentially methylated regions (DMRs) of the parental species retained in hybrids, but only a few of the DMRs exhibited nonadditive variation, and these were not significantly correlated with changes in gene expression [23]. Changes in DNA methylation levels in *Brassica napus* do not adequately explain subgenomic dominance [24]. However, the interaction between DNA methylation and accessible chromatin regions may be the key to differential gene expression [25, 26]. In *Osmanthus fragrans*, the expression of *CCD4*, a key gene for ionone synthesis, was correlated with the gene's promoter region exhibiting different methylation levels and chromatin accessibility [25]. Genome-wide chromatin accessibility profiles in 18 deletion mutants of *Arabidopsis* with CG, CHG, or CHH DNA methylation revealed that DNA methylation affects chromatin accessibility in all three sequence contexts [26]. Most chromatin accessibility regions are hypomethylated, and acclimation-induced changes in DNA methylation can influence the expression of proximal and distal genes [27–29].

*Brassica napus* (NCBI:txid3708; AACC; 2*n* = 4*x* = 38) is generated by hybridizing from *Brassica rap*a (AA; 2*n* = 2*x* = 20) and *Brassica oleracea* (CC; 2*n* = 2*x* = 18) [30]. DNA methylation patterns and ACRs in *B. napus*, shaped by breeding and natural selection, are linked to its adaptive and agronomic traits [31–34]. Methylation changes between the globular embryo and mature green stages and leaves show that most promoter methylation is established early in seed development and remains stable [31]. DNA methylation also plays a key role in enhancing hybrid vigor in early seed and seedling growth [32]. It helps reduce the activity of transposable elements during hybridization by affecting DNA methylation levels through nonadditively expressed siRNA clusters [33]. Furthermore, the C subgenome has been found to have greater chromatin accessibility than the A subgenome, mainly due to the genes in the C subgenome being unique and not shared [34]. This indicates a complex relationship between epigenetic regulation and the traits of *B. napus*.

Interspecific hybridization between *B. napus* and *B. rapa* effectively broadens the genetic base of *B. napus*. This method takes advantage of the significant potential of natural triploids in polyploid breeding [35, 36]. Additionally, these allotriploid hybrids provide a unique opportunity to explore the effects of polyploidization on global gene expression, allowing for precise comparisons between F_1_ hybrids and their diploid or tetraploid progenitors [37–39]. Previous research on interspecific hybrids of *B. napus* and *B. rapa* has primarily focused on genomic structural variations and differences in gene expression [35, 36, 40–43]. However, the potential mechanisms by which interspecific hybridization induces differences in gene expression leading to phenotypic changes in hybrids have not been extensively explored. We obtained a series of allotriploid hybrids (AAC, 2*n* = 29) by crossing *B. rapa* with *B. napus*. Two F_1_ hybrids exhibiting distinct characteristic biases were selected for RNA-seq, sRNA-seq, ATAC-seq, and whole genome bisulfite sequencing (WGBS) to comprehensively evaluate the relationship between phenotypic changes induced by gene expression differences after interspecific hybridization and DNA methylation and accessible chromatin regions. We reveal that novel ACRs drove transgressive upregulated genes in hybrids and that transgressive upregulated genes were significantly enriched for metal ion binding. Additionally, DNA methylation plays a role in repressing gene expression within ACRs, and unmethylated ACRs are more transcriptionally active. This information can be used to develop breeding programs to improve crop performance.

## Materials and Methods

### Plant material

Two *B. napus* cultivars (s*70*, A_s_A_s_C_s_C_s_; *yu25*, A_y_A_y_C_y_C_y_, allotetraploid) were selected as the maternal parent, and *B. rapa* cultivar (*B. campestris* L. ssp. *chinensis* var. *purpuria* Hort.; A_h_A_h_, diploid) were selected as the paternal parent. The F_1_ allotriploid hybrids were generated by crossing between two different *B. napus* and the *B. rapa* species. All plant materials were grown under the same field conditions located at the Huazhong Agricultural University (30°28ʹN, 114°21ʹW). For sampling, the plant materials were collected at 10.00–11.00 am in December (average temperature, 8°C). Stem epidermal tissue below the top of the fifth true leaf was collected and immediately frozen in liquid nitrogen. These samples were then used for subsequent ATAC-seq, WGBS, sRNA-seq, and RNA-seq library construction and for determining ions and metabolites.

### ATAC-seq analysis

The low-quality reads and adapters from the raw ATAC-seq data were filtered and removed using Trimmomatic (RRID:SCR_011848) [44]. The clean data were aligned to the *B. napus* (Zhongshuang 11, *ZS11*) reference genome by Bowtie2_v2.5.2 (RRID:SCR_016368) [45, 46]. The mapped reads in sam format were converted to bam format using SAMtools_v1.9 (RRID:SCR_005227) [47]. Subsequently, MACS2_v2.2 (RRID:SCR_013291) peak-calling software was used to identify ATAC-seq peaks [48]. The overlapping peaks over 50 bp in the biological replicates were considered ACRs. The genomic distribution of ACRs and associated genes was confirmed using the ChIPseeker tool (RRID:SCR_021322) [49]. Differential binding events were identified using the DiffBind_v6.0 package [50]. The motifs of the ACRs were identified using HOMER (RRID:SCR_010881) [51]. The term “silent ACR” refers to ACRs identified in parents (reads ≥ 5) but not in F_1_ hybrids (reads = 0). On the other hand, the term “novel ACR” refers to ACRs identified in F_1_ hybrids (reads ≥ 5) but not in parents (reads = 0).

### WGBS analysis

We then used BatMeth2-align with default parameters to map the filtered WGBS reads to the *B. napus* (*ZS11*) genome [45]. The sequences covering five or more cytosine sites were set as valid methylation sites. Finally, BatMeth2-Meth2BigWig was used to generate BigWig files to identify and visualize differentially methylated regions (DMRs) in IGV [52]. Only cytosine regions with adjusted *P*-values < 0.05 and DNA methylation differences more significant than 0.3, 0.2, and 0.1 (for CG, CHG, and CHH, respectively) were considered DMRs.

### RNA-seq analysis

Trimmomatic was used to remove barcode adaptors, and low-quality reads [44]. The filtered reads were aligned to the *B. napus (ZS11*) reference genome using HISAT2_v2.2.0 with default parameters [53]. The uniquely mapped reads were filtered using SAMtools_v1.9 [47]. Counting and normalizing transcripts per million mapped reads (FPKM) were performed on BAM files using StringTie_v2.1.4 [54]. Genes with FPKM > 1 were defined as expressed genes. Genes with an adjusted *P*-value < 0.05 found by DESeq2 and a |log_2_ fold change| ≥ 2 were assigned as differentially expressed [55].

### sRNA-seq analysis

The raw sequencing reads were trimmed using cutadapt (RRID:SCR_011841) v3.1 to remove adapters. Subsequently, sRNAs between 18 and 30 nt in length were selected and mapped to the *B. napus* (*ZS11*) genome and defined into sRNA clusters using Shortstack_v3.8.4 (RRID:SCR_010834) [56]. sRNA-mapped reads were normalized to the total cleaned reads for further analysis.

### Elemental mass spectrometry analysis

The concentrations of mineral elements in the stem and epidermis were measured using an inductively coupled plasma-mass spectrometer (ICP-MS) (NexION 300D; Perkin Elmer, Massachusetts, USA). The replicate samples for each line were pooled and then subjected to oven-drying at 80°C for a minimum of 72 h, followed by grinding. Approximately 0.2 g of the dried ground powder was placed in a PTFE digestion tube with 6 ml of concentrated nitric acid, and the tube was tightly sealed and processed in a closed vessel acid digestion microwave (MARSXpress; CEM Corporation, Matthews, NC, USA). After digestion, each digested sample was diluted to 10 ml with deionized water and elemental analysis was performed using an ICP-MS in standard mode, monitoring 15 elements.

### Measurement of metabolites

The measurement of soluble sugars, soluble proteins, total phenolics, total flavonoids, total anthocyanins, and proanthocyanidins in the stem epidermal tissue was performed as described in previous studies [57]. All samples were quantified in triplicate in three independent biological replicates.

### Electrophoretic mobility shift assay

Polymerase chain reaction (PCR) amplified the full‐length coding DNA sequences of *BnaA03.bZIP11* and *BnaC03.bZIP11* which were then cloned into pGEX4T-2 (GST) to express the BnaA03.bZIP11 and BnaC03.bZIP11 proteins in *Escherichia coli* DE3 strain in the presence of 0.5 mM IPTG at 28°C for 12 h. The recombinant BnaA03.bZIP11 and BnaC03.bZIP11 protein was purified using a GST 4FF prepacked gravity column (C600911; Sangon Biotech, Shanghai, China). For the electrophoretic mobility shift assay (EMSA), the Cy5-labeled probes and recombinant proteins were mixed in an EMSA/Gel-Shift binding buffer (GS005; Beyotime, Shanghai, China) at 25°C for 20 min in the presence or absence of unlabeled competitor DNA. The reaction mixture was then electrophoresed on 6% nondenaturing polyacrylamide gels under ice-water conditions.

### Dual-luciferase assay

The full length of *Bna.bZIP11* was amplified by PCR and inserted into *pGreenII-62SK* for transient overexpression. The 2-kb promoter sequence of *BnaA06.UF3GT* was also amplified by PCR and inserted into the *pGreenII 0800-LUC* vector using a ClonExpress II One Step Cloning Kit (C112; Vazyme, Nanjing, China). According to previously described methods, all relevant effector and reporter constructs were transformed into *Arabidopsis* mesophyll protoplasts [58]. The dual luciferase assay was then conducted according to the instructions from the Dual-Luciferase Reporter Assay System (E1910; Promega, Madison, WI, USA). The data were expressed as the ratio of firefly to renilla luciferase activity (Fluc/Rluc). Each data point was based on at least three replicates, and three independent experiments were performed for each experiment.

### Functional enrichment analysis

Gene function descriptions were obtained from the *B. napus* (*ZS11*) reference genome. The GO enrichment analysis was performed using agriGO2, and terms with a false discovery rate (FDR) < 0.05 were considered significant.

### Identification of additive genes

To identify additive and nonadditive gene expression, we constructed independently *in silico* “hybrids” by combining the RNA-seq data from sequenced parental individuals in a 1:1 ratio for the *B. napus* (*s70* and *yu25*) and *B. rapa* (Hort) datasets. This ratio reflects the respective 1:2 genomic contribution of the parents to the F_1_ hybrids. The differentially expressed genes (DEGs) were identified by comparing the expression levels of genes between the hybrids and *in silico* hybrids, using the criteria of an adjusted *P*-value < 0.05 and a |log_2_ fold change| ≥ 1.5. These genes are indicated as additive if shown to be no differential, and vice versa for nonadditive genes.

### Identification of dosage-dependent and independent genes

The identification of dosage-dependent and dosage-independent genes followed the method described in a previous study [59]. Briefly, Pearson correlation tests, employing the Benjamini–Hochberg FDR method, were conducted to assess the relationship between gene expression and genotype dosage for the A-subgenome (1/2:2/3:1) and C-subgenome (1/2:1/3:0) genes. The Pearson correlation and multiple test corrections were performed using the adjustment method in R. Genes exhibiting a significant correlation between expression and dosage (*R*^2^ > 0.64, FDR < 0.05) were classified as dosage-dependent. In contrast, those with no significant correlation were deemed dosage-independent. For instance, the Pearson correlation coefficient for genomic dosage (1/2:2/3:1) and gene expression values (1.72, 1.96, 2.54) of the gene *BnaA01G0002000ZS* is 0.99, with an *R*^2^ of 0.99, indicating that this gene is dosage-dependent.

### RT-qPCR assay

A quantitative reverse transcription PCR (RT-qPCR) assay was performed according to a previous report [41]. The melting curve of RT-qPCR was analyzed to ensure the presence of only one peak. The expression level of each gene was calculated using the 2^−ΔΔCT^ method. All analyses were performed at least three times. The *BnaActin7* gene (XM_013858992) was used as an internal control. All primers were listed in Supplementary Table S1.

## Results

### Resynthesized allotriploid *B. napus*–*B. rapa* hybrids and phenotypic characterization

**Figure 1:**
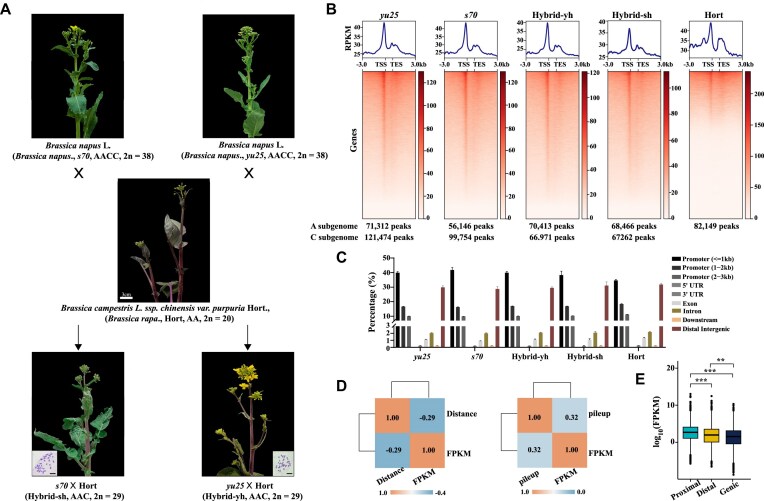
Overview of chromatin accessibility in the F_1_ hybrids and their parental lines. (A) The images showed the phenotypes of the allotriploid F_1_ hybrids and their parents at the flowering stage (scale bars, 3 cm) and the typical chromosome number (scale bars, 10 µm) of the allotriploid *Brassica* hybrids. (B) Chromatin accessibility around genes in the F_1_ hybrids (Hybrid-sh and Hybrid-yh), and their relative parents (maternal parents: *s70* and *yu25*; paternal parent: Hort). (C) The bar graph showed the proportion of ACR regions in the genome of F_1_ hybrids and their relative parents. (D) Comparison of gene expression and ACR intensity (left) and ACR distance (right). (E) Expression levels of genes associated with different types of ACRs. Boxplots show the median (horizontal line). The upper and lower quartiles are the boundaries of the boxplots. The outlier was the data point outside the whiskers of the boxplot. The Kruskal–Wallis test was used to calculate the *P*-value (*P* < 2.2 × 10−16).

We previously generated two allotriploid hybrids of *Brassica* species by crossing two *B. napus* inbred lines (s*70*, A_s_A_s_C_s_C_s_; *yu25*, A_y_A_y_C_y_C_y_) with a *B. rapa* (Hort, A_h_A_h_) (Materials and Methods). The two *B. napus* inbred lines had green stems, while the *B. rapa* had red stems (Fig. 1A). The resulting allotriploid hybrids, designated Hybrid-sh (s70 × Hort, AsAhCs) and Hybrid-yh (yu25 × Hort, AyAhCy), had 29 chromosomes. Phenotype observations revealed that the stem of Hybrid-yh was red, resembling that of the paternal line Hort. In contrast, Hybrid-sh displayed the morphological characteristics of the maternal line s70, characterized by a stem color that was lighter than that of Hybrid-yh (Fig. 1A). Additionally, the soluble protein and sugar contents in the two allotriploid F_1_ hybrids were approximately 141–168 mg/g and 51–56 mg/g higher, respectively, compared with their respective parental lines (Supplementary Fig. S1A). The total phenolic content in the allotriploid hybrids was similar to that of the maternal lines and exceeded that of the paternal line (Student's *t*-test, *P* < 0.05; Supplementary Fig. S1A). The total flavonoid content in the allotriploid F_1_ hybrids was approximately 7.2–8.1 mg/g, which was higher than that of the maternal line but lower than that of the paternal line (Supplementary Fig. S1B). Notably, the flavonoid content of Hybrid-yh was higher than that of Hybrid-sh (Student's *t*-test, *P* < 0.05; Supplementary Fig. S1B).

### Genome-wide identification of ACRs in F_1_ hybrids and their respective parentals

We conducted RNA-seq and ATAC-seq of stem epidermis from two allotriploid hybrids (Hybrid-sh and Hybrid-yh) and their corresponding parental lines to investigate the genome-wide transcriptional dynamics during allotriploid hybridization. The RNA-seq generated an average of 20 million clean reads, with 93.4% successfully mapped to the reference genomes (Supplementary Table S2). Meanwhile, ATAC-seq produced an average of 47 million clean reads per replicate (Supplementary Table S3). Spearman’s rank correlation and principal component analysis (PCA) revealed high-quality RNA-seq and ATAC-seq datasets (Supplementary Fig. S2A, B). The average sequencing depth of accessibility chromatin regions for *yu25, s70*, Hybrid-yh, Hybrid-sh, and Hort were quantified, resulting in 17-, 18-, 20-, 19-, and 17-fold values, respectively (Supplementary Fig. S3). The genome browser results showed that consistent ACR peaks were identified in three biological replicates of the parental line and F_1_ hybrids (Supplementary Fig. S3). The ATAC-seq peaks were enriched in the following samples: 192,786 in *yu25*,155,900 in *s70*,137,384 in Hybrid-yh, 135,728 in Hybrid-sh, and 82,149 in Hort (Fig. 1B). The ACR peaks in Hort were less than maternal lines and F_1_ hybrids, which may be due to the Hort lacking the C subgenome. We then compared the ACR peaks in different subgenomes in F_1_ hybrids and parental lines. The result showed that 71,312, 56,146, 70,413, 68,466, and 82,149 ACR peaks were identified in the A subgenome of *yu25, s70*, Hybrid-yh, Hybrid-sh, and Hort, respectively (Fig. 1B). However, in the C subgenome, 121,474, 99,754, 66,971, and 67,262 ACR peaks were identified in *yu25, s70*, Hybrid-yh, and Hybrid-sh (Fig. 1B), indicating that the ACR densities in the C-subgenome are suppressed by hybridization. The ACRs were significantly enriched at gene transcription start sites (TSS) (Fig. 1C). The majority of ACRs were between 200 and 500 base pairs (bp) in length, with the highest enrichment observed at 250 bp (Supplementary Fig. S4A). Overall, the ATAC-seq data provide a comprehensive and reliable overview of ACRs in allotriploid hybrids and their parental plants.

The distribution of ACRs on the genome was categorized based on their proximity to genes, which were classified as genic (overlapping with a gene), proximal (within 2 kb of a gene), and distal (more than 2 kb away from any gene). Our analysis revealed a positive correlation between gene expression levels and the presence of ACRs in the TSS region (*R* = 0.32, Kruskal–Wallis test, *P* < 2.2 × 10^−16^) (Fig. 1D). Conversely, gene expression showed a negative correlation with the distance of TSS region ACRs (*R* = −0.29, Kruskal–Wallis test, *P* < 2.2 × 10^−16^) (Fig. 1D). On average, genes associated with proximal (including TSS and TTS) ACRs were more highly expressed than the genes related to distal and genic ACRs (Fig. 1E). In contrast to distal and genic ACRs, proximal ACR played a predominant role in regulating gene expression, suggesting that the positioning of ACRs is closely related to their transcriptional regulation, which is consistent with previous studies [16, 27, 60].

### ACRs show different convergent distributions after interspecific hybridization

**Figure 2:**
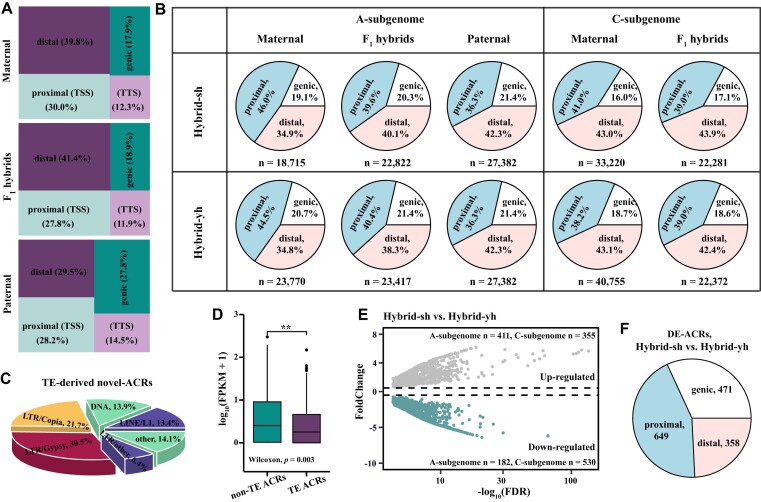
Distribution of ACRs in F_1_ hybrids and their parental lines. (A) Graph showing the proportion of ACR regions in genic (overlapping with a gene), proximal (within 2 kb upstream or downstream of a gene, including transcription start sites (TSSs) and transcription termination sites (TTSs)], and distal (more than 2 kb away from any gene) in the F_1_ hybrids and their parents. (B) Genomic distribution of ACRs in the two hybrids and their parents. The percentage of ACRs categorized as genic, proximal, and distal within different subgenomes (A/C). (C) Pie chart showig the proportion of TE-derived novel ACRs (TE-driven ACRs when >50% of the region overlaps with TEs). (D) Boxplots showed the expression levels of genes associated with non-TE-mediated ACRs and TE-mediated ACRs. The Kruskal–Wallis test was used to calculated the *P*-value (**P < 0.01). (E) MA plot showing differentially expressed ACRs (DE-ACRs) between Hybrid-sh and Hybrid-yh. (F) Pie charts showing the distribution of DE-ACRs in genic, proximal, and distal.

Interspecific hybridization may lead to the activation of ACR in unopened regions [27, 61]. After examining the variation in the distribution of ACRs within genes and across the genomes of parents and hybrids, we found that more than 40% of the ACRs in the F_1_ hybrids were located in distal regions (Fig. 2A). In comparison, only 28% were distributed distally in the paternal line (Fig. 2A). After hybridization, there was a 2.8% decrease in proximal ACRs, with 39.7% in F_1_ hybrids compared with 42.5% in the parents (Supplementary Fig. S4B). Conversely, distal ACRs showed a 6.7% increase in F_1_ hybrids compared with parental ACRs (Supplementary Fig. S4C), confirming a distinct ACR distribution between parents and F_1_ hybrids. These results suggest that the hybridization process induces changes in the distribution and abundance of accessible chromatin regions, particularly in gene regions and their proximity.

The parental lines *yu25, s70*, and Hort exhibited 23,770, 18,715, and 27,382 ACRs in the A subgenomes, respectively (Fig. 2B). In contrast, 40,755 and 33,220 ACRs were identified in the C subgenomes of *yu25* and *s70*, respectively, showing the divergent distribution of ACRs between the A subgenome and the C subgenome. In total, 23,417 and 22,822 ACRs were identified in the A subgenomes of the Hybrid-yh and Hybrid-sh (Fig. 2B). In comparison, 22,372 and 22,281 ACRs were detected in the C subgenomes of Hybrid-yh and Hybrid-sh, respectively (Fig. 2B). We found no significant difference in the number of ACRs in the A subgenome in F_1_ hybrids and their parents, indicating a convergent distribution of ACRs in the A subgenome in interspecific hybridization. The number of ACRs in the C subgenome in F_1_ hybrids was about half of that in the maternal lines (Fig. 2B), demonstrating that the number of ACRs was positively correlated with the subgenomic dosage. We further analyzed whether there is immediate convergence in the distribution of different types of ACRs (genic, proximal, and distal ACRs) across subgenomes in interspecific hybrids. There was no difference in genic ACR between the F_1_ hybrid and its parental A subgenome; however, significant differences were observed between proximal and distal ACRs (Fig. 2B). This suggests that genic ACRs in the A subgenome converge immediately following interspecific hybridization. Notably, in the A subgenome, proximal ACRs were most abundant in the maternal line, followed by F_1_ hybrids, while the paternal line exhibited the least abundance; in contrast, distal ACRs show the opposite trend (paternal > F_1_ hybrids > maternal) (Fig. 2B). However, no significant differences were found in the proportions of the genic, proximal, and distal ACRs in the C subgenome (Fig. 2B), suggesting that the different types of ACRs converge immediately upon the halving of the C subgenome following interspecific hybridization.

### Identification of novel ACRs in F_1_ hybrids

We identified 969 novel ACRs and 25 silent ACRs in F_1_ hybrids (Supplementary Fig. S5A), following the criteria ACRs with reads > 5 in F_1_ hybrids and read = 0 in their parental lines were defined as novel ACRs; ACRs with reads = 0 in F_1_ hybrids and reads > 5 in their parental lines were defined as silenced ACRs [27]. Among these, there were 209 and 760 novel ACRs in the A and C subgenomes, respectively (Supplementary Fig. S5B), indicating that the formation of novel ACRs was greater in the C subgenome than in the A subgenome. The distribution of novel ACRs revealed that the proportions of genic, proximal, and distal ACRs were 35.7%, 32.4%, and 32.9%, respectively (Supplementary Fig. S5C). Additionally, the distribution of novel ACRs was primarily observed at the telomeres situated distally to the centromere (Supplementary Fig. S5D). Of these novel ACRs, 375 (38.7%) were classified as TE-driven ACRs, with LTR/Gypsy-type retrotransposons playing a significant role, accounting for 30.5% compared with other types of transposable elements (Fig. 2C). We found that gene expression in the proximal regions of non-TE-mediated ACRs was significantly higher than that of TE-mediated ACRs (Fig. 2D). TE-mediated ACRs may exhibit lower gene expression levels due to the influence of methylation on both the TE and its adjacent sequences [12,62,63]. In contrast, non-TE-mediated ACRs are typically unaffected by TEs; consequently, DNA methylation levels in these regions may be reduced, thereby facilitating the expression of genes in neighboring areas.

Differentially expressed accessible chromatin regions (DE-ACRs) were then compared between the two hybrids. In total, 1,478 DE-ACRs were identified in Hybrid-yh compared with Hybrid-sh (Fig. 2E). The numbers of differentially up-regulated ACRs and differentially down-regulated ACRs were 766 and 712, respectively (Fig. 2E). Interestingly, in the F_1_ hybrids, the number of DE-ACRs in the C subgenome (855) was significantly higher than that in A subgenome (593) (Fig. 2F), demonstrating a positive correlation between the number of DE-ACRs and genome size. There were greater numbers of proximal DE-ACRs than genic and distal DE-ACRs (Fig. 2F). These results suggest that the distribution of DE-ACRs was uneven across the genome in F_1_ hybrids.

### Nonadditive gene expression in F_1_ hybrids

**Figure 3:**
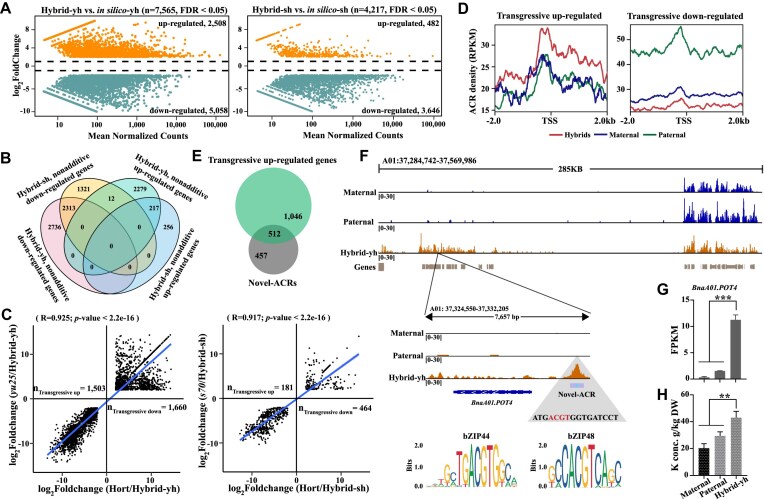
Nonadditive genes in F_1_ hybrids. (A) The MA plot showed differentially expressed genes (DEGs) between the F_1_ hybrids and the mid-parent value (MPV). (B) Venn diagram showing the overlap of Hybrid-yh nonadditive down-regulated genes, nonadditive up-regulated genes, Hybrid-sh nonadditive down-regulated genes, and nonadditive up-regulated genes. (C) Scatterplot showing the distribution of differential expression levels of Hybrid-sh (left) and Hybrid-yh (right) versus maternal (*x* axis) and paternal (*y* axis) parents. (D) Graph showing the ACR densities of transgressive up-regulated (left) and down-regulated (right) genes. (E) Venn diagram showing an overlap of novel ACRs and transgressive up-regulated genes. (F) The genome browser showed ATAC-seq peaks around *Potassium Transporter 4* (*POT4*) in the parents and Hybrid-yh, and the enrichment of the *bZIP44* and *bZIP48* motif (ACGT) in the promoter region of *POT4*. (G,H) Bar graph showing the expression level of *BnaA01.POT4* (G), and potassium levels in Hybrid-yh and its relative parents (H). Error bars indicate the mean ± SD of three biological replicates. In (H) and (I), Student’s *t*-test was used to calculate significance: **P < 0.01, ***P < 0.001.

Previous studies have emphasized the potential role of nonadditive genes in resynthesized plant materials [41, 64]. Gene expression quantification was then performed to examine the pre-existing gene expression levels of allotriploid hybrids and their parents. This involved comparing *in silico* hybrids (where the RNA-seq data combined maternal and paternal in a 1:1 ratio) with the F_1_ hybrids. The results showed that most expressed genes exhibited additive expression patterns in Hybrid-yh (82.9%) and Hybrid-sh (90.1%) (Supplementary Fig. S6A). More than 86% of the additive genes were conserved in the two hybrids (Supplementary Fig. S6B). GO enrichment analysis of conservative additive genes revealed enrichment for cellular, rhythmic, metabolic, and carbon–nitrogen utilization (Supplementary Table S4). This suggests that conserved additive genes are necessary for normal plant growth and development. However, a total of 7,565 and 4,217 genes showed nonadditive expression in Hybrid-yh and Hybrid-sh, respectively (Fig. 3A). In Hybrid-yh, there were 2,508 nonadditively up-regulated genes and 5,058 nonadditively down-regulated genes (Fig. 3A). In contrast, Hybrid-sh exhibited 482 nonadditively up-regulated genes and 3,646 nonadditively down-regulated genes (Fig. 3A). The nonadditively up-regulated genes in Hybrid-yh were mainly associated with responses to biotic and abiotic stimuli, immune responses, and metabolic processes (Supplementary Table S5). Conversely, nonadditively up-regulated genes in Hybrid-sh were enriched in ion transport, response to hormones, and metabolic processes (Supplementary Table S6). This suggests that GO enrichment of nonadditively up-regulated genes in F_1_ hybrids creates an enhanced capacity for metabolite production and improved tolerance to environmental conditions. Furthermore, 2,530 identical nonadditive expressed genes (2,313 down-regulated and 217 up-regulated) were isolated in both Hybrid-yh and Hybrid-sh (Fig. 3B). These down-regulated genes were associated with primary meristem tissue development, organic acid transmembrane transport, phloem transport, and auxin polar transport by GO enrichment analysis (Supplementary Table S7). In contrast, the up-regulated genes were enriched with response to external biotic stimulus, lipid metabolic process, and defense response pathways (Supplementary Table S8).

There are two main categories within nonadditive genes: expression-level dominance (ELD) and transgressive [64]. Based on gene expression level, the transgressive genes could be further categorized as transgressive up-regulated genes and transgressive down-regulated genes [64]. Approximately 3,163 and 645 transgressive expression genes were identified in Hybrid-yh and Hybrid-sh, respectively (Fig. 3C). In Hybrid-sh, the number of down-regulated genes (464) was significantly higher than the number of up-regulated genes (181) (Fig. 3C). In comparison, no difference was observed between the number of up-regulated (1,503) and down-regulated (1,660) transgressive genes in Hybrid-yh (Fig. 3C). These results suggest that the expression levels of genes in Hybrid-sh show relative stability in the hybrid offspring, with a greater likelihood of inheriting the expression pattern from the parent.

Previous studies have demonstrated a significant positive correlation between ACRs and gene expression [17, 27]. In F_1_ hybrids, chromatin accessibility was found to be higher than in the parents for transgressively up-regulated genes (hybrids > maternal = paternal) (Fig. 3D). Conversely, for transgressive down-regulated genes, the chromatin accessibility was significantly lower in F_1_ hybrids than in the parents (paternal > maternal > hybrids) (Fig. 3D). Notably, more than half of the novel ACRs were found to target transgressive up-regulated genes (Fig. 3E). The transgressive up-regulated genes in Hybrid-yh showed significant GO enrichment in metal ion binding, especially magnesium ion, calcium ion, and potassium ion binding, as well as secondary metabolic processes (Supplementary Fig. S7A). In particular, two genes, *POT4* (*Potassium Transporter 4*) and *POT2* (*Potassium Transporter 2*), which are involved in the regulation of potassium ion uptake and transport in plants [65, 66], were identified. Two novel ACRs associated with *BnaC04.POT2* and *BnaA01.POT4* were identified in their promoters (Fig. 3F, Supplementary Fig. S7B). The expression of *BnaC04.POT2* and *BnaA01.POT4* was significantly higher in Hybrid-yh than the parental lines (Fig. 3G, Supplementary Fig. S7C). As expected, Hybrid-yh has a significantly higher potassium content than the parental lines (Fig. 3H). Previous studies have shown that the loss of the *bZIP48* gene function in rice results in increased sensitivity to zinc deficiency, while the *bZIP44* gene is involved in the plant's response to cadmium tolerance [58, 67]. Notably, the bZIP transcription factor binding *cis*-element, ACGT, was identified in the novel ACR of *BnaA01.POT4* promoter region (Fig. 3F). Based on these findings, we hypothesize that *bZIP48* and *bZIP44* may interact with *BnaA01.POT4*, thereby playing a role in potassium ion transport.

To investigate the differences in mineral element content between two F_1_ hybrids and their parents, we analyzed 16 mineral elements, including 5 major elements (e.g., magnesium) and eight trace elements (e.g., boron) (Supplementary Table S9). The results showed that the major elements (e.g., calcium) were significantly higher in F_1_ hybrids than their parents. However, the trace elements (e.g., boron) showed additive effects, and other elements such as iron, zinc, copper, and molybdenum showed no significant differences between F_1_ hybrids and their parents. These results suggest that genetic factors regulate the mineral element content in F_1_ hybrids and are not solely determined by the additive effects of the parent plants.

### *Bna.bZIP11* in SPA-ACR regulates the expression of *BnaA06.UF3GT* to promote the accumulation of anthocyanins

**Figure 4:**
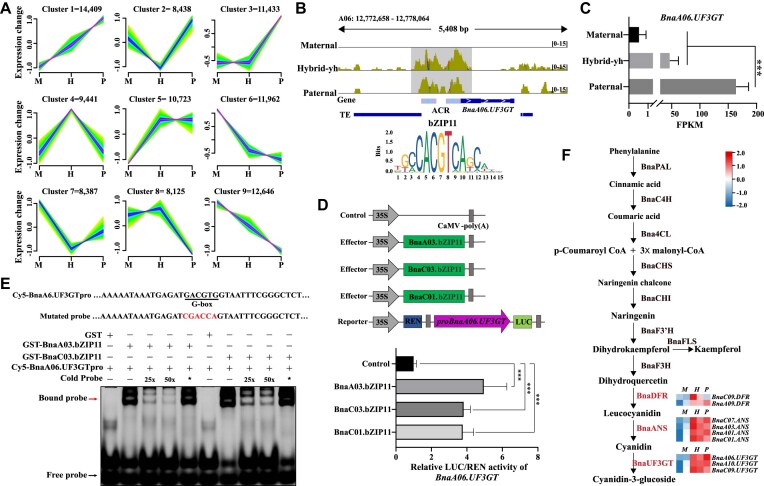
A-subgenome SPA-ACR drives bZIP11 to promote UF3GT expression in paternal and Hybrid-yh. (A) Graphs showing the *c*-means soft clustering analysis of chromatin accessibility levels of A subgenomes in the Hybrid-sh and its relative parents. (B) The genome browser showed the ATAC-seq peaks around *BnaA06.UF3GT* in the parents and Hybrid-sh, and the bZIP11 motif (ACGT) enrichment in the promoter region of *BnaA06.UF3GT*. (C) Bar graph showing the expression level of *BnaA06.UF3GT* in Hybrid-sh and its relative parents. (D) Schematic diagrams of the effector and reporter constructs for the dual-luciferase transcriptional activity assay. The LUC/REN activities in Arabidopsis protoplasts were analyzed following co-transformation with the (*pBnaA06.UF3GT:LUC*) and various reporter constructs: *p35S* (control), *p35S:BnaA03.bZIP11, p35S:BnaC03.bZIP11*, and *p35S:BnaC01.bZIP11*. (E) EMSA results showing that *BnaA03.bZIP11* and *BnaC03.bZIP11* could directly bind to the *BnaA06.UF3GT* promoter. Increasing amounts (25- and 50-fold) of the unlabeled DNA fragments were added as competitors. The red arrow indicates the shift bands. Red letters indicate the mutated G-box *cis*-element within the *BnaA06.UF3GT* promoter. An asterisk indicates that the mutated cold competitor probes were added to the panel. (F) Anthocyanin biosynthesis pathway. The colored boxes show the gene expression heatmap from RNA-seq, normalized by log_2_FPKM (calculation method). Genes responsible for the leucocyanidin, cyanidin, and cyanidin-3-glucoside steps are shown in red. BnaPAL, phenylalanine ammonialyase; BnaC4H, cinnamic acid 4-hydroxylase; Bna4CL, 4-coumarate: coenzyme A ligase; BnaCHS, chalcone synthase; BnaCHI, chalcone isomerase; BnaF3H, flavanone 3-hydroxylase; BnaF3ʹH, flavonoid 3ʹ-hydroxylase; BnaDFR, dihydroflavonol-4-reductase; BnaANS, anthocyanidin synthase; BnaUF3GT, UDP glucose-flavonoid 3-O-glucosyltransferase. In (C) and (D), error bars indicate the mean ± SD of three biological replicates. Student’s *t*-test was used to calculate significance: ***P < 0.001.

The *c*-means clustering method was then used to classify the differential peaks according to chromatin accessibility levels in F_1_ hybrids and their respective parents, resulting in nine clusters labeled C1–C9 (Fig. 4A, Supplementary Fig. S8A). For ACRs, the ATAC-seq peaks were detected in only one parent, while they were not detected in the other parent, termed single-parent activation ACRs (SPA-ACRs). These SPA-ACRs could be further categorized into two patterns: SPA-M ACRs, where the ACRs were detected in the maternal and F_1_ hybrids but not in the paternal; and SPA-P ACRs, where the ACRs were detected in the paternal and F_1_ hybrids but not in the maternal. Since the Hort lacks the C-subgenome, it is reasonable to expect a limited number of ATAC-seq peaks in the Hort materials compared to those in the maternal (AACC) and F_1_ hybrid (AAC) genomes. We then focused the analysis only on peaks corresponding to the A subgenomes. A total of 8,125, 9,681, and 22,885 SPA-ACRs were identified in the A_s_ (A subgenome of *s70, B. napus*), A_y_ (A subgenome of *yu25, B. napus*), and A_h_ (A genome of *B. rapa*) subgenomes (Fig. 4A, Supplementary Fig. S8A), which were represented in clusters C5 and C8, respectively (Fig. 4A, Supplementary Fig. S8A). The SPA-ACR can lead to the emergence of single parental expression (SPE), a gene expressed in only one parent but is silent in the other parent after interspecific hybridization [68]. On average, 336 genes expressed in the F_1_ hybrids were exclusively expressed in the maternal parents (SPE-M, A_s_ and A_y_), and 277 genes expressed in the F_1_ hybrids were exclusively expressed in the paternal parent (SPE-P) in the A genome (A_h_) (Supplementary Fig. S8B). Approximately 20% of the SPE genes were correlated with SPA-ACR (Supplementary Fig. S8C).

The datasets were then prioritized based on the occurrence of proximal SPA-P ACRs and SPE-P genes. Notably, the top-10 ranked in the SPA-P genes showed a significant presence of genes associated with anthocyanin synthesis (SupplementaryFig. S8D). For example, we found that the chromatin accessibility of *BnaA06.UF3GT*, which encodes the flavonoid 3-O-glucosyltransferase (UF3GT) enzyme [69], was more significant in the hybrids and the paternal promoter region compared with in the maternal promoter region (Fig. 4B). The expression of the *BnaA06.UF3GT* was significant in F_1_ hybrids and paternal individuals, whereas no expression was detected in the maternal plant (Fig. 4C). Analysis using the HOMER algorithm showed that the *BnaA06.UF3GT* gene might be regulated by the *BnabZIP11* transcription factor (Fig. 4B). To investigate this, a luciferase (LUC) gene driven by a ∼2 kb promoter of *BnaA06.UF3GT* was used as a reporter, and three *BnabZIP11* genes were driven by the *CaMV 35S* promoter (Fig. 4D). Co-transformation of the vector containing *BnaA03.bZIP11, BnaC03.bZIP11*, and *BnaC01.bZIP11* with the *pBnaA06.UF3TG-LUC* construct significantly increased LUC/REN activities by 4.95-, 3.81-, and 3.76-fold, respectively, compared with the expression of *pBnaA06.UF3TG-LUC* alone (Fig. 4D). Further analysis revealed the binding of the BnabZIP11 transcription factor to the G-box elements upstream of *BnaA06.UF3GT* (Fig. 4E), suggesting that *BnabZIP11* may directly bind to the G-box elements upstream of the *BnaA06.UF3GT* promoter and thereby positively regulate the expression of *BnaA06.UF3TG*. The expression patterns of genes involved in anthocyanin synthesis and related transcription factors were also highly expressed in the F_1_ hybrids and the paternal parent (Fig. 4F, Supplementary Fig. S9). This finding suggests a possible reason for the high accumulation of total anthocyanins and phenolics in the paternal and hybrid plants (Supplementary Fig. S1B). The activation of ACRs by a single parent after interspecific hybrid genome recombination is positively associated with gene regulation, which may contribute to the nonadditive phenotypic traits observed in resynthesized F_1_ hybrids. The interaction between the activated ACRs and gene expression patterns may lead to altered gene regulatory networks, resulting in unique phenotypic traits in hybrids that differ from those of either parent.

### Hybridization-induced DNA methylation related to parents in F_1_ hybrids

The process of hybridization often results in significant reprogramming of global DNA methylation. This can be considered an “epigenetic shock” resulting from the fusion of different epigenomes from the parental plants [22, 70, 71]. Therefore, we evaluated the total methylation levels in hybrids and compared them with the parental methylation levels. The sequencing depth of WGBS was approximately 30-fold genome coverage, and the bisulfite conversion was greater than 99% in all samples (Supplementary Table S10). The sRNA-seq data generated an average of 27 million clean reads per replicate, of which 93.4% were mapped to the reference genome (Supplementary Table S11). PCA plots and Spearman’s rank correlation coefficients generated from the sRNA-seq and WGBS libraries showed clear clustering patterns and reproducibility across biological replicates (Supplementary Fig. S10A, B). At the chromosomal level, a bias in the total methylation levels of CG, CHG, and CHH towards the hypermethylated parent was observed (Supplementary Fig. S11A). Further, the methylation levels of TE bodies in F_1_ hybrids and their parental lines across the whole genome were analyzed. We identified 25.9-30.6%, 35.0-40.2%, and 39.7-44.3% TEs in F_1_ hybrids CG, CHG, and CHH contexts, respectively, which were not significantly different from the maternal line (*s70* and *yu25*) (Supplementary Fig. S11B). In comparison to the paternal line (Hort), the proportions of TE no differences in CG, CHG, and CHH contexts that were observed in F_1_ hybrids were 13.5-13.9%, 19.6-20.0%, and 22.6-22.7% (Supplementary Fig. S11B). This indicates that the F_1_ hybrids tended to inherit higher methylation levels from the parent with higher methylation.

**Figure 5:**
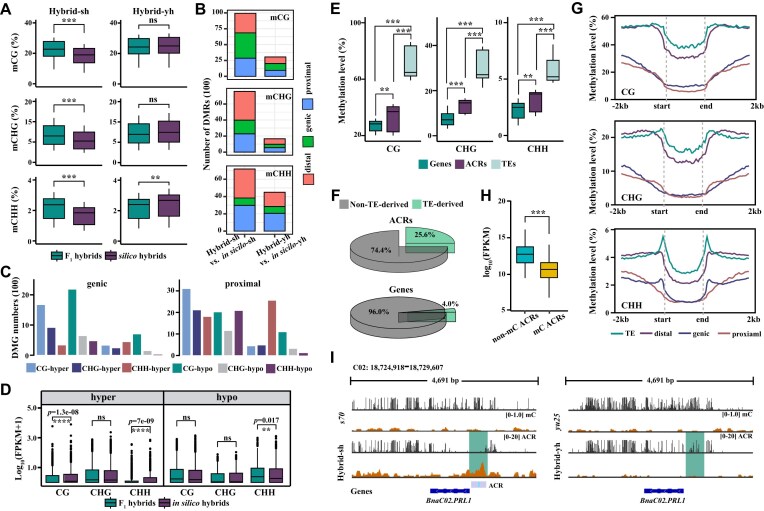
Divergence of DNA methylation landscape between parents and hybrids. (A) Boxplots showing the total DNA methylation level (CG, CHG, and CHH) of F_1_ hybrids (Hybrid-sh (left) and Hybrid-yh (right)) and *in silico* hybrids (Wilcoxon rank-sum test; **P < 0.01; ***P < 0.001; ns, no significant difference). (B) Bar graph showing the number of differentially methylated regions (DMRs) between F_1_ hybrids and *in silico* hybrids in 200 bp bins. Different colors indicate the distribution of DMRs in the distal (red), genic (green), and proximal (blue) regions. (C) Bar graph showing the number of genic (left) and proximal (right) DMGs in the Hybrid-sh and Hybrid-yh. (D) Boxplots showing the expression levels of hyper- and hypo-DMGs in F_1_ hybrids and *in silico* hybrids with methylation sites in proximal regions. The *y* axis represents the gene expression level log_10_(FPKM+1) (Wilcoxon rank-sum test; **P < 0.01; ***P < 0.001; ns, no significant difference). (E) Boxplots showing the methylation level (CG, CHG, and CHH) across genes, TEs, and ACRs. (F) Pie chart showing the proportion of TE-derived and non-TE-derived ACRs (top) and genes (bottom). The TE-derived ACR was defined as having >50% of the region overlapping with TEs. (G) Graphs showing the methylation level (CG, CHG, and CHH) of TE ACRs, distal ACRs, genic ACRs, and proximal ACRs. (H) Boxplots showing the expression level of genes associated with methylated and nonmethylated ACRs. (I) The genome browser showed DNA methylation loci around *BnaC02.PRL1* in F_1_ hybrids (Hybrid-sh and Hybrid-yh) and their relative parents. In (E) and (H), the upper and lower quartiles are boundaries of the boxplots. The Kruskal–Wallis test was used to calculated the *P*-value: **P < 0.01; ***P < 0.001).

To investigate the changes in DNA methylation in F_1_ hybrids after interspecific hybridization, two *in silico* hybrids (*in silico*-sh and *in silico*-yh) were first constructed by combining maternal and paternal WGBS data in a 1:1 ratio. All DNA methylation levels (CG, CHG, and CHH) of Hybrid-sh were higher than those of *in silico*-sh, whereas no significant difference in the methylation levels of CG and CHG was found between Hybrid-yh and *in silico*-yh (Fig. 5A). However, the methylation level of CHH was lower in Hybrid-yh than *in silico*-yh (Fig. 5A). Compared to *in silico* hybrids, 24,693 and 9,214 differentially methylated regions (DMRs) were identified in Hybrid-sh and Hybrid-yh, respectively (Fig. 5B). There were fewer hypo-DMRs (9,701) compared with hyper-DMRs (13,492) in Hybrid-sh (Supplementary Fig. S12A). In total, 9,214 DMRs were found in Hybrid-yh, and nearly half of the DMRs (49%) were CHH-DMRs (Supplementary Fig. S12A). Compared with proximal and distal DMRs, a more significant increase in genic DMRs was identified for CG methylation in both Hybrid-sh and Hybrid-yh (Fig. 5B). For CHG methylation, the distribution of DMRs was as follows: distal > proximal > genic (Fig. 5B). The distribution of DMRs at gene locations suggests that the context of DNA methylation influences this distribution. More than 68% of the differentially methylated genes (DMGs) were proximal DMGs (Fig. 5C). Transcript levels of proximal DMGs were then compared between F_1_ hybrids and *in silico* hybrids. No significant difference was observed in the expression of DMGs associated with CHG (Fig. 5D). However, the expression of hyper DMGs of CHH was significantly higher in F_1_ hybrids than *in silico* hybrids (Fig. 5D). In contrast, hypo DMGs showed the opposite trend (Fig. 5D).

In addition, we identified 9,464 DMRs in Hybrid-sh and Hybrid-yh, and 2,370, 1,289, 1,518 hyper-DMRs and 2,317, 1,256, and 714 hypo-DMRs in Hybrid-sh CG, CHG, and CHH contexts compared with Hybrid-sh (Supplementary Fig. S12B). Furthermore, in the context of differential methylation loci (DMLs), we also observed 5,323, 2,271, and 5,902 hyper-DMLs, as well as 5,448, 1,840, and 3,503 hypo-DMLs in the CG, CHG, and CHH contexts, respectively, compared with Hybrid-sh (Supplementary Fig. S12B). The difference in the number of hyper-DMRs (hyper-DMLs) and hypo-DMRs (hypo-DMLs) in the two hybrids was caused by the DMRs (DMLs) in the CHH context, which highlighted unbalanced hyper- and hypo-CHH methylation changes between the two hybrids.

TE involvement was detected in approximately 55.6% of DMRs; further analysis revealed that 21.5% and 39.1% of these DMRs were LINE and LTR (Supplementary Fig. S13A). To gain additional insight into the relationship between sRNA-mediated DNA methylation and hybridization-induced hyper- and hypo-DMRs, we analyzed changes in sRNA enrichment within hyper- and hypo-DMRs. In hyper-DMRs, we observed a significant increase in sRNA accumulation *in silico* compared with F_1_ hybrids, whereas the opposite trend was seen in hypo-DMRs (Supplementary Fig. S13B). This highlights the close relationship between DMRs and sRNA accumulation.

### DNA methylation in ACR associated with TEs in F_1_ hybrids

Previous reports indicate that DNA methylation is critical in maintaining and losing chromatin accessibility [26, 28, 29]. When comparing the DNA methylation levels of TEs, gene bodies, and ACRs, we found that ACRs had significantly higher methylation levels compared with gene bodies but lower than TEs (Kruskal-Wallis test, *P* < 2.2 × 10^−16^, Fig. 5E). Further analysis revealed that 25.6% of ACRs and 4.0% of genes were derived from TEs (Fig. 5F), indicating a substantial overlap between ACRs and TEs. This finding suggests that the increased DNA methylation levels observed in ACRs may be associated with TEs, potentially explaining the observed differences between ACRs and gene bodies.

The level of DNA methylation in ACRs varies depending on their position in the genome. ACRs with a CG context show different levels of DNA methylation, with a consistent decrease in methylation levels between TEs, distal, genic, and proximal regions (TE > distal > genic > proximal; Kruskal−Wallis, *P* < 2.2 × 10^−16^; Fig. 5G). However, in the context of CHG and CHH, there was no significant difference between the methylation levels of the genic and proximal regions (Fig. 5G). Overall, TE and distal ACR showed significantly higher methylation levels compared with genic and proximal ACRs (Fig. 5G). This suggests that TE and distal regions may require higher levels of DNA methylation for stability. In contrast, genic and proximal regions may be more sensitive to gene expression regulation, resulting in lower DNA methylation levels. Gene expression analysis also supported this observation: the expression of genes associated with ACRs lacking DNA methylation was significantly higher compared with genes associated with methylated ACRs (Fig. 5H). For example, in Hybrid-sh, an open chromatin region was observed in the promoter region of *BnaC02.PRL1*, which did not inherit the methylation sites from the parent; conversely, in Hybrid-yh, the situation was reversed (Fig. 5I). RNA-seq and RT-qPCR results showed that *BnaC02.PRL1* was not detected in Hybrid-yh, but was significantly expressed in Hybrid-sh (Student’s *t*-test, *P* < 0.05, Supplementary Fig. S13C). This indicates that DNA methylation plays a role in repressing gene expression within ACRs, and unmethylated ACRs may be more transcriptionally active.

### Genome dosage affects accessible chromatin regions in F_1_ hybrids

**Figure 6:**
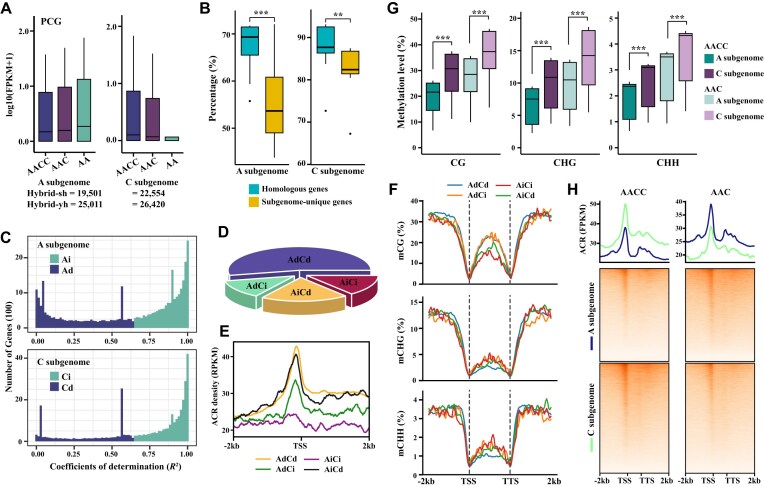
Effects of genomic imbalance on accessible chromatin regions and DNA methylation in F_1_ hybrids. (A) The expression level (top) and number (bottom) of dose-dependent genes in the A and C subgenomes. (B) Boxplots showing the proportion of dose-dependent homologous gene pairs and dose-dependent genome-unique genes in the A and C subgenomes. (C) Graphs showing the number of dose-dependent and dose-independent homologous genes in the A and C subgenomes. Pearson’s correlation was used to test and divide all homologous genes into two groups based on the coefficient of determination (*R*^2^) to compare the characteristics of dose-dependent and dose-independent homologous genes. Homologous genes with statistically significant correlations were designated as dose-dependent A (Ad) and C (Cd) genes, while those with in significant correlations were designated as dose-independent A (Ai) and C (Ci) genes. (D) Pie chart showing the percentage of AdCd, AdCi, AiCd, and AiCi duplicated genes. (E,F) Graphs showing the ACR density (E) and DNA methylation level (CG, CHG, and CHH) (F) between AdCd, AdCi, AiCd, and AiCi duplicated genes. (G,H) Diagrams showing the distribution of DNA methylation level (G) and ACR density (H) of A and C subgenomes in AACC and AAC.

Genome-wide dose-dependent and independent regulation contribute to the evolution and gene expression of plant polyploids [59, 72], and genome imbalance in AAC hybrids may lead to changes in gene expression and variation in epigenetics. We then calculated the correlation coefficient (*R*-values) between the expression of 96,992 genes and the relative doses of the genes in the A and C subgenomes. In total, 19,501–25,011 and 22,554–26,420 dose-dependent genes were identified in the A and C subgenomes, respectively (Fig. 6A). Interestingly, approximately 56% and 16% of the dose-dependent and dose-independent genes, respectively, were consistent in the two F_1_ hybrids (Supplementary Fig. S14A), indicating that dose-dependent genes are more conserved in F_1_ hybrids.

Pearson’s correlation test was used to divide all the homologous genes into two groups based on the coefficient of determination (*R*^2^) to compare the characteristics of dose-dependent and dose-independent homologous genes. Homologous genes with statistically significant correlations were designated dose-dependent A (Ad) and C (Cd) genes. In contrast, those with insignificant correlations were designated dose-independent A (Ai) and C (Ci) genes. The proportion of dose-dependent genes among the homologous genes in the A/C subgenome was higher than that of genome-unique genes (Fig. 6B). Among the four categories of homologous genes (AdCd, AdCi, AiCd, and AiCi), AdCd had the highest number (20,888), followed by AiCd (7,746). At the same time, AdCi and AiCi had the lowest number (4,927 and 5,552, respectively) (Fig. 6C,D). Chromatin stacking was observed to be highest in the ACR for AdCd, followed by AdCi, and lowest for AiCi (Fig. 6E), indicating that dose-dependent homologous genes have increased chromatin accessibility, making them more susceptible to regulation by transcriptional and regulatory factors. Higher levels of DNA methylation were found in the CG context for AdCd and AdCi compared with AiCd and AiCi, while in the CHG and CHH contexts, AdCd had the lowest methylation levels among the four categories and AiCi had the highest (Fig. 6F). In both AACC and AAC, the methylation level of the C subgenome was higher than that of the A subgenome, regardless of the dose of the C subgenome (Fig. 6G). This suggests that factors beyond genomic dosage regulate DNA methylation levels. In AACC, the ACR level of the C subgenome was higher than that of the A subgenome; however, in AAC, the ACR level of the A subgenome was significantly higher than that of the C subgenome (Fig. 6H). This demonstrates that in AAC, doubling the dosage of the A subgenome results in a significant increase in the ACR level of the A subgenome, highlighting the strong effect of genome dosage on accessible chromatin regions. Dose-dependent and dose-independent genes were identified in the A and C subgenomes. The dose-dependent genes exhibited higher chromatin openness. Notably, differences in the A subgenome were specifically located in the TSS and upstream regions, whereas differences in the C subgenome were observed more broadly across the genome (Supplementary Fig. S14B).

## Discussion

Interspecific hybridization is essential in plant breeding and genetic improvement [7, 73, 74]. In this study, we resynthesized two interspecific F_1_ hybrids by crossing different genotypes of *B. napus* and *B. rapa* to elucidate the intricate relationship between accessible chromatin regions, DNA methylation, and the expression of transgressive genes. The interplay between DNA methylation, TEs, and sRNA contributes to the dynamic landscape of ACRs during interspecific hybridization, resulting in distinct gene expression patterns across the genome.

### Accessible chromatin regions and DNA methylation differ in genome dosage effects

In comparative studies focusing on gene expression patterns within polyploid plant species, it has been observed that most genes across different subgenomes exhibit coordinated expression behavior, either in a dosage-dependent or dosage-independent manner [59, 72]. Specifically, in the resynthesized *Arabidopsis* allotetraploid, approximately 56% of the alleles showed congruent expression patterns, with a predominant dosage-dependent expression (46% TdAd) complemented by a smaller proportion of dosage-independent expression (10% TiAi) [59]. Similar trends were noted in synthetic *B. napus* and its derivatives, where 58% of A- and C-subgenome genes align in the same direction, with 40% exhibiting dosage-dependent expression (AdCd) and 18% showing dosage-independent expression (AiCi). Conversely, 42% have divergent expression patterns (22% AdCi and 20% AiCd) [72]. In our study, 66% of genes displayed coherent expression, either dosage-dependent (54% AdCd) or dosage-independent (12% AiCi). However, a significant proportion of 32% still presents contrasting expression dynamics (10% AdCi and 24% AiCd). Notably, there remains a substantial fraction of genes (ranging from 32% to 44%) that manifest discordant expression patterns between subgenomes, reflecting different genetic backgrounds between *Arabidopsis* and *Brassica* species, and also the intricate interplay between gene dosage effects and the regulatory networks that modulate gene expression in polyploid genomes. In polyploids, allele mutations may be buffered by extra gene copies, reducing negative selection and increasing survival chances for individuals with mutations in dosage-dependent genes [59]. These findings underscore the prevalence of coordinated and independent regulatory mechanisms influencing gene expression in polyploid genomes. This necessitates a nuanced understanding of the genetic and epigenetic factors that govern these patterns.

At the mechanistic level, chromatin accessibility is essential for gene transcription because it allows transcription factors and RNA polymerase to bind to DNA and initiate gene expression [13]. Changes in chromatin accessibility can directly impact gene expression levels, leading to significant dosage effects [27, 59, 60]. In other plant species (e.g., cotton, sorghum, and *Arabidopsis*), accessible chromatin regions were consistently found to be positively correlated with the expression of nearby genes, and highly expressed genes exhibited distinct peaks around their transcription start sites (TSS) [14, 16, 27]. In contrast, DNA methylation has a more indirect effect on gene expression. It can influence gene expression by inhibiting transcription factor binding and recruiting inhibitory protein complexes [75, 76]. These mechanisms may interact complexly and involve feedback regulation, making the relationship between DNA methylation and gene expression nonlinear and resulting in an insignificant dose effect. In the resynthesized *Arabidopsis* allotetraploid, CHG methylation levels were higher in the genic regions of dosage-independent alleles than dosage-dependent ones [59]. Conversely, the 5′ regions of dosage-dependent alleles exhibited greater methylation than those of dosage-independent alleles [59]. Furthermore, DNA methylation is dynamic and highly specific to different cell types [77]. Despite alterations in genome dosage, DNA methylation levels can remain relatively stable or change only in particular cell types [78, 79]. On the other hand, chromatin accessibility is also dynamic and cell-type specific, but it is more responsive to changes in genome dosage [27, 80]. For example, significantly different distributions of DNase I hypersensitive sites (DHSs) were observed between the A and D subgenomes in F_1_ cotton hybrids [27]. The genome size and polyploidy also affect the distal DHS distribution [27]. These indicate that changes in chromatin structure serve as a rapid mechanism for regulating gene expression in direct response to alterations in genome dosage.

### Interspecific hybridization triggers genome recombination leading to novel accessible chromatin regions

During plant hybridization, the unequal retention of epigenetic marks, particularly ACRs activated by single parents or novel, can significantly affect gene expression and trait development in hybrid offspring. In *Camellia sinensis*, a large number of accessible chromatin regions is observed after interspecific hybridization [61]. The “newborn DHS” (nbDHS) after cotton polyploidization suggests that these nbDHS may arise from transposable elements during or after polyploidization [27]. We found 512 novel ACRs near the transgressive genes in F_1_ hybrids, 38.7% of these novel ACRs were classified as TE-driven ACRs, suggesting that interspecific hybridization triggers genome recombination that activates ACR formation. SPA-ACRs suggest that only one parent may control the expression pattern of certain gene regions during genome recombination. The maintenance of this expression pattern may be influenced by asymmetric chromatin openness, which can lead to increased expression or silencing of specific genes in hybrid offspring [61, 81]. Notably, most SPA-ACRs are found within the gene bodies that encode the proteins, suggesting a possible relationship between the expression pattern of single-parent activation and structural features of the gene’s transcriptional activity region. In addition, the higher expression levels of genes adjacent to SPA-ACRs provide further evidence of a direct link between this expression pattern and increased gene expression. In wheat, expression of the *ph1* gene is associated with chromatin remodeling, which alters chromatin structure so that only genes from a particular parent are expressed [82].

Differences in the proportions of DHSs in distal and proximal regions have been observed during wheat polyploidization, suggesting that the distribution of DHSs varies among different genomes [12]. Our results indicate that interspecific hybridization induces changes in the distribution and abundance of chromatin regions accessible to genic and proximal regions. We found that the number of DE-ACRs in the two hybrids was highest in the proximal, followed by the genic, and lowest in the distal regions (Fig. 2G). The distribution of DE-ACRs in the proximal region may be related to the regulation of gene expression [14]. Proximal regions usually include the promoters directly regulating gene expression [12]. Therefore, the activity of these regions may change significantly in different hybrids or cell types, leading to the detection of more proximal DE-ACRs. In addition, the DNA methylation level of the distal region was considerably higher than that of the proximal region (Fig. 5G), indicating that the distal region was relatively stable.

### Accessible chromatin regions and DNA methylation jointly regulate gene expression

There is a close relationship between DNA methylation and open chromatin regions [25, 26]. DNA methylation typically occurs on CpG islands, where its primary role is suppressing gene expression. Methylated CpG islands often result in a more condensed chromatin structure, limiting the binding of transcription factors and other regulatory proteins and ultimately repressing gene expression [70, 83]. In contrast, open chromatin regions have a more flexible chromatin structure, allowing easier binding of transcription factors and regulatory proteins, thus promoting gene expression. Significant positive correlations have been observed between chromatin accessibility and gene expression levels in nearby genes in our study. We further found that the DNA methylation level of the ACR located at the proximal end of the gene is much lower than that of the TE and distal regions (Fig. 5G). This may be because the proximal ACR usually contains promoters and enhancers and is an important region for gene transcription regulation. Low levels of DNA methylation help keep these regions open, making it easier for transcription factors and other regulatory proteins to bind, thereby promoting gene expression [25, 29]. Genome-wide chromatin accessibility maps for 18 *Arabidopsis* mutants that lack CG, CHG, or CHH methylation demonstrated that DNA methylation in all three sequence contexts influences chromatin accessibility [26]. Variations in DNA methylation found in lettuce may affect chromatin accessibility, altering the expression levels of associated proximal and distal genes [29]. Similar results were observed in rice domestication [84]. The soybean *CMT* mutant, *Gmcmt*, exhibits significant hypomethylation at non-CG (CHG and CHH) DNA methylation sites, which enhanced chromatin accessibility and significantly regulated the expression of hundreds of functionally relevant genes (e.g., *Golden-Like10* (*GmGLK10*)), which further strengthen photosynthesis and unexpectedly boosted rhizobia's fixation efficiency [85]. These results suggest that DNA methylation and a delicate balance of ACRs are essential for maintaining normal gene expression patterns.

## Conclusions

**Figure 7:**
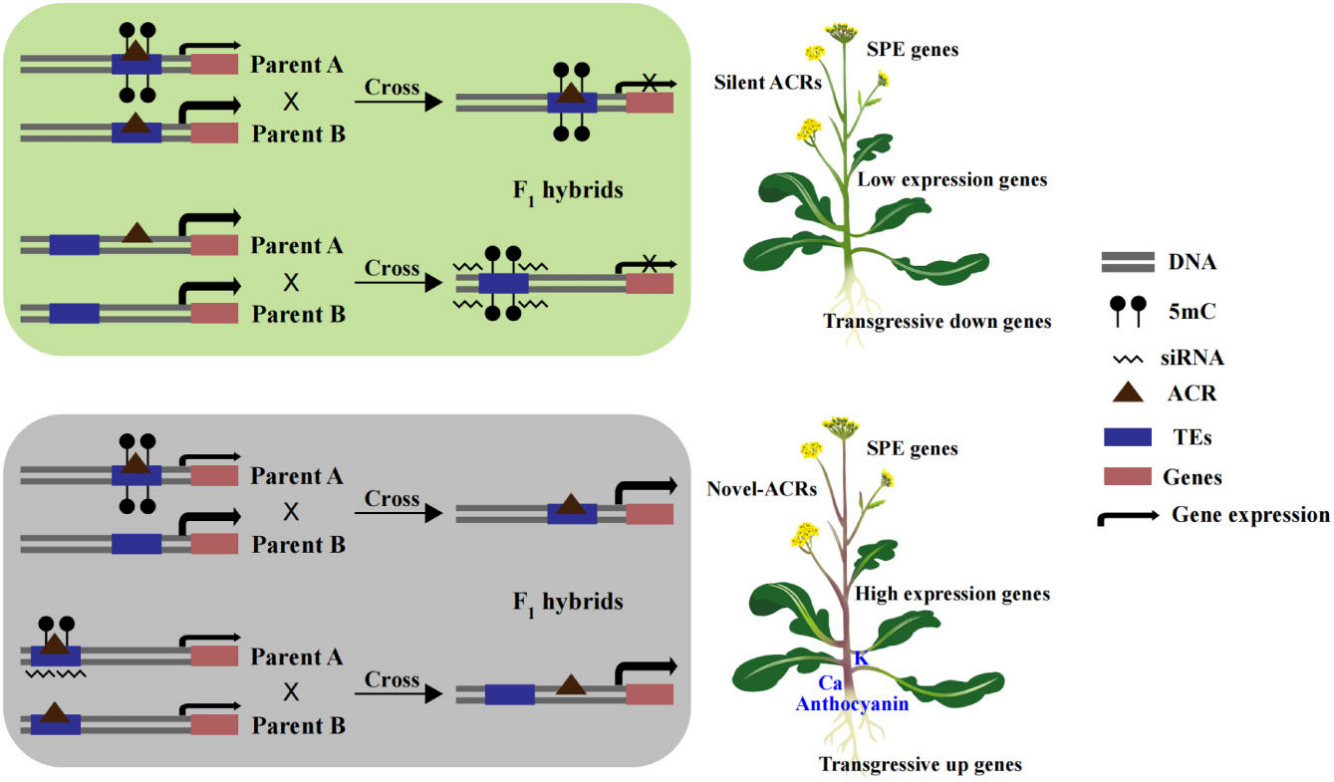
Model of hybridization-induced variation of accessible chromatin regions and DNA methylation sites in F_1_ hybrids. F_1_ hybrids inherit accessible chromatin regions (ACRs), and sRNA, and DNA methylation loci from their parents, resulting in gene transgressive and single parental expressions (SPEs). Silent ACR: ACRs were identified in parents but not in F_1_ hybrids. Novel ACR: ACRs were identified in F_1_ hybrids but not in parents. Transgressive up genes: the gene expression level of the F_1_ hybrids was higher than that of the parents. Transgressive down genes: the gene expression level of F_1_ hybrids was lower than that of the parents. SPE: genes are expressed only in one parent and F1 hybrids but not in the other.

Overall, our findings provide valuable insights into the role of accessible chromatin and DNA methylation in driving gene expression in hybrids (Fig. 7). We observed significant differences in the expression of nonadditive genes among the different F_1_ hybrids, with up-regulated transgressive genes associated with metal ion accumulation and SPE genes related to anthocyanin accumulation. This study contributes to our preliminary understanding of how accessible chromatin regions and DNA methylation regulate metabolite and ion accumulation in F_1_ hybrids.

## Supplementary Material

giaf029_Supplemental_File

giaf029_Authors_Response_To_Reviewer_Comments_Original_Submission

giaf029_GIGA-D-24-00365_Original_Submission

giaf029_GIGA-D-24-00365_Revision_1

giaf029_Reviewer_1_Report_Original_Submission Zefu Lu -- 10/14/2024

giaf029_Reviewer_1_Report_Revision_1 Zefu Lu -- 2/5/2025

giaf029_Reviewer_2_Report_Original_Submission Chaobo Tong -- 12/25/2024

giaf029_Reviewer_2_Report_Revision_1 Chaobo Tong -- 1/23/2025

## Data Availability

All the sequence data generated in this study have been deposited in the BIG data under the BioProject accession number PRJCA023096 and NCBI BioProject: PRJNA1154317. All additional supporting data are available in the *GigaScience* repository, GigaDB [86].

## Additional Files

**Supplemental materials and methods:** The details of the ATAC-seq, WGBS, RNA-seq, sRNA-seq experiments and statistical methods are described in the supplemental materials.

**Supplementary Figure S1:** Determination of primary metabolites.

**Supplementary Figure S2:** Quality control of ATAC-seq and RNA-seq datasets.

**Supplementary Figure S3:** Genome browser showing ATAC-seq signals in the 192-kb and 259-kb region in two hybrids and their parents. Tracks show normalized values of three biological replicates for each sample.

**Supplementary Figure S4:** Distribution of ACRs.

**Supplementary Figure S5:** Distribution of novel ACRs.

**Supplementary Figure S6:** Additive genes in two hybrids.

**Supplementary Figure S7:** CO enrichment of transgressive up-regulated gene in Hybrid-yh.

**Supplementary Figure S8:** Characterization of SPA-ACRs in F_1_ hybrids.

**Supplementary Figure S9:** Heatmap showing the expression levels of anthocyanin biosynthesis genes and the related transcription factors in F_1_ hybrids and their parents. The colored boxes represent the gene expression heatmap, normalized by log_2_FPKM values obtained from RNA-seq.

**Supplementary Figure S10:** Quality control of WGBS and sRNA-seq datasets.

**Supplementary Figure S11:** Differences in methylation levels between F_1_ hybrids and their parental lines.

**Supplementary Figure S12:** Characterization of DMRs and DMLs in F_1_ hybrids.

**Supplementary Figure S13:** Density of TEs and sRNAs in DMRs.

**Supplementary Figure S14:** Dose-dependent and dose-independent genes distributions in the A and C subgenomes.

**Supplementary Table S1:** Primer sequences used for RT-qPCR analyses.

**Supplementary Table S2:** Statistics of RNA-seq data and reads mapping for all samples.

**Supplementary Table S3:** Statistics of ATAC-seq data and reads mapping for all samples.

**Supplementary Table S4:** GO enrichment analysis of conservative additive genes in two hybrids.

**Supplementary Table S5:** GO enrichment analysis of nonadditively up-regulated genes in Hybrid-yh.

**Supplementary Table S6:** GO enrichment analysis of nonadditively up-regulated genes in Hybrid-sh.

**Supplementary Table S7:** GO enrichment analysis of shared down-expressed genes in two hybrids.

**Supplementary Table S8:** GO enrichment analysis of shared up-expressed genes in two hybrids.

**Supplementary Table S9:** The contents of 16 ionic components in the F_1_ hybrids and their parents.

**Supplementary Table S10:** Statistics of WGBS data and reads mapping for all samples.

**Supplementary Table S11:** Statistics of sRNA-seq data and reads mapping for all samples.

## Abbreviations

ACRs: Accessible Chromatin Regions; ATAC-seq: Assay for Transposase-Accessible Chromatin with high throughput sequencing; bp: base pairs; Brassica napus: B. napus; Brassica rapa: B. rapa; CCD4: carotenoid cleavage dioxygenase 4; CREs: cis-regulatory elements; DE-ACRs: Differentially expressed accessible chromatin regions; DEGs: differentially expressed genes; DMRs: differentially methylated regions; FDR: false discovery rate; FPKM: Fragments Per Kilobase of exon model per Million mapped fragments; GmGLK10: GOLDEN-LIKE10; GO: Gene Ontology; ICP-MS: inductively coupled plasma-mass spectrometer; LUC: luciferase; NCBI: The National Center for Biotechnology Information; PCA: principal component analysis; POT2: POTASSIUM TRANSPORTER 2; POT4: POTASSIUM TRANSPORTER 4; RNA-seq: RNA sequencing; RT-qPCR: Reverse Transcription quantitative Polymerase Chain Reaction; small RNA-seq: sRNA sequencing; SPA-ACR: Single-parent activation accessible chromatin region; SPE-M: Single parent expression-maternal; SPE-P: Single parent expression-paternal; TE: Transposable element; TSS: transcription start sites; TTS: transcription termination site; WGBS: Whole Genome Bisulfite Sequencing; ZS11: Zhongshuang 11.

## Notes

The author responsible for the distribution of materials integral to the findings presented in this article following the policy described in the Instructions for Authors is: Cheng Dai (cdai@mail.hzau.edu.cn)
