# Supplementary material for: Interspecific hybridization in Brassica species leads to changes in agronomic traits through the regulation of gene expression by chromatin accessibility and DNA methylation
Source: Gigascience. 2025 Apr 22;14:giaf029. doi: 10.1093/gigascience/giaf029 (PMC12012897; doi:10.1093/gigascience/giaf029)
Supplement: giaf029_Supplemental_File [file giaf029_supplemental_file.docx]

**Title Page**

**Interspecific hybridization in Brassica species leads to changes in agronomic traits through the regulation of gene expression by chromatin accessibility and DNA methylation**

Chengtao Quan^1,2^, Qin Zhang^1,2^, Xiaoni Zhang^1,2^, Kexin Chai^1,2^, Guoting Cheng^3^, Chaozhi Ma^1,2^, and Cheng Dai^1,2^

1 National Key Laboratory of Crop Genetic Improvement, Huazhong Agricultural University, Wuhan 430070, China

2 Hubei Hongshan Laboratory, Wuhan, 430070, China

3 College of Informatics, Huazhong Agricultural University, Wuhan 430070, China.

The author responsible for the distribution of materials integral to the findings presented in this article following the policy described in the Instructions for Authors is:

Cheng Dai (cdai@mail.hzau.edu.cn)

To whom correspondence should be addressed.

Dr. Cheng Dai

National Key Laboratory of Crop Genetic Improvement, Huazhong Agricultural University, Wuhan 430070, P.R. China

Email: cdai@mail.hzau.edu.cn

**Supplemental materials and methods**

**ATAC-seq**

For each biological replicate, the collected plant tissue was cut into small pieces with blade in 500 mL lysis buffer (15 mM Tris-HCl pH7.5, 20 mM NaCl, 80 mM KCl, 0.5 mM spermidine, 5 mM 2-mercaptoethanol and 0.2% Triton X-100). After confirming nuclear integrity, purified nuclei were resuspended in a 50 μL Tn5 transposase integration reaction and incubated at 37°C for 30 min. The Tn5 transposase-digested DNA fragments were then recovered using a MinElute PCR Purification Kit (Qiagen, Cat No./ID: 28004), and followed by purification and amplification. The purified library was then sequenced on an Illumina Novaseq platform by Novogene Gene Technology (Novogene, Beijing, China). All ATAC-seq profiles were generated from at least of three independent biological replicates.

**Whole genome bisulfite sequencing**

Genomic DNA was extracted from 10 samples using the cetyl trimethylammonium bromide (CTAB) method. A lambda DNA spike-in was utilized to correct for non-conversion rates of uracil, with 1 ng of methyl-free lambda DNA added to 1 μg genomic DNA as an internal reference for the conversion test. Bisulfite conversion of DNA was carried out using the EZ DNA Methylation Gold Kit (Zymo Research in Irvine, California, USA). The Bisulfite-Seq Library Prep Kit for Illumina (Novogene in Beijing, China) was used to construct whole genome bisulfite sequencing (WGBS) libraries, which were then sequenced on an Illumina HiSeq X10 platform at a depth of 30-fold. Two biological replicates were performed.

**RNA-seq and sRNA-seq**

Total RNA was extracted with the RNeasy Plant Mini Kit (Qiagen, Cat No./ID: 74904) according to the manufacturer's instructions. This preparation was split and used for both RNA-seq and sRNA-seq. Library construction and deep sequencing were performed using the Illumina HiSeq 4000 Platform according to the manufacturer’s protocols (Novogene, Beijing, China). Three biological replicates were performed.

**Statistics and reproducibility**

Statistical significance was determined using R (https://r-project.org). The Wilcoxon rank sum test and the χ2 test were performed using the *Wilcoxon.test* function and the *chisq.test* function, respectively, from the R package.

**
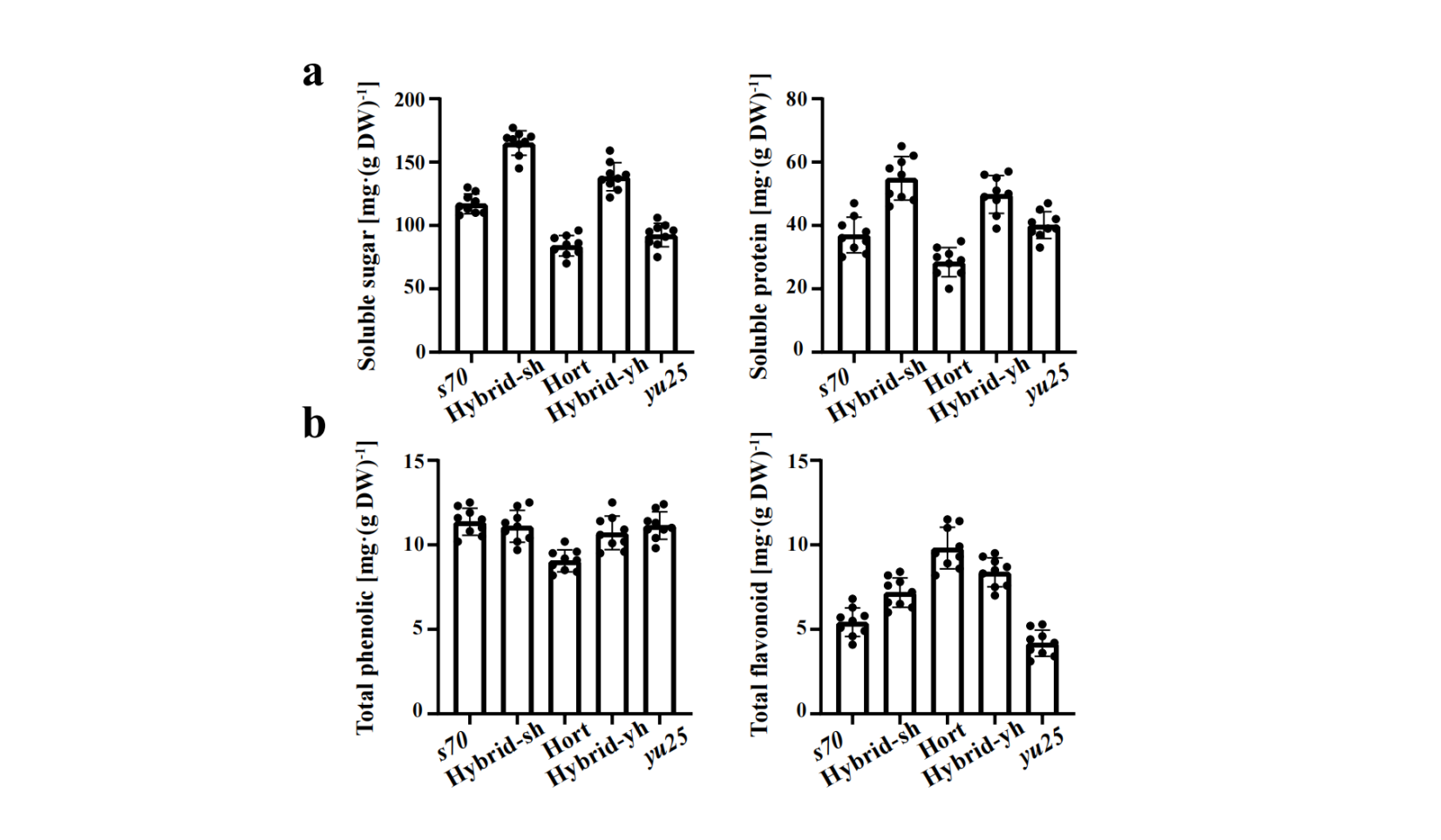
Figure S1. Determination of primary metabolites.** The bar graph showed the content of soluble sugars and proteins **(a)**, total phenols, and flavonoids **(b)** in F_1_ hybrids and their relative parents.

**
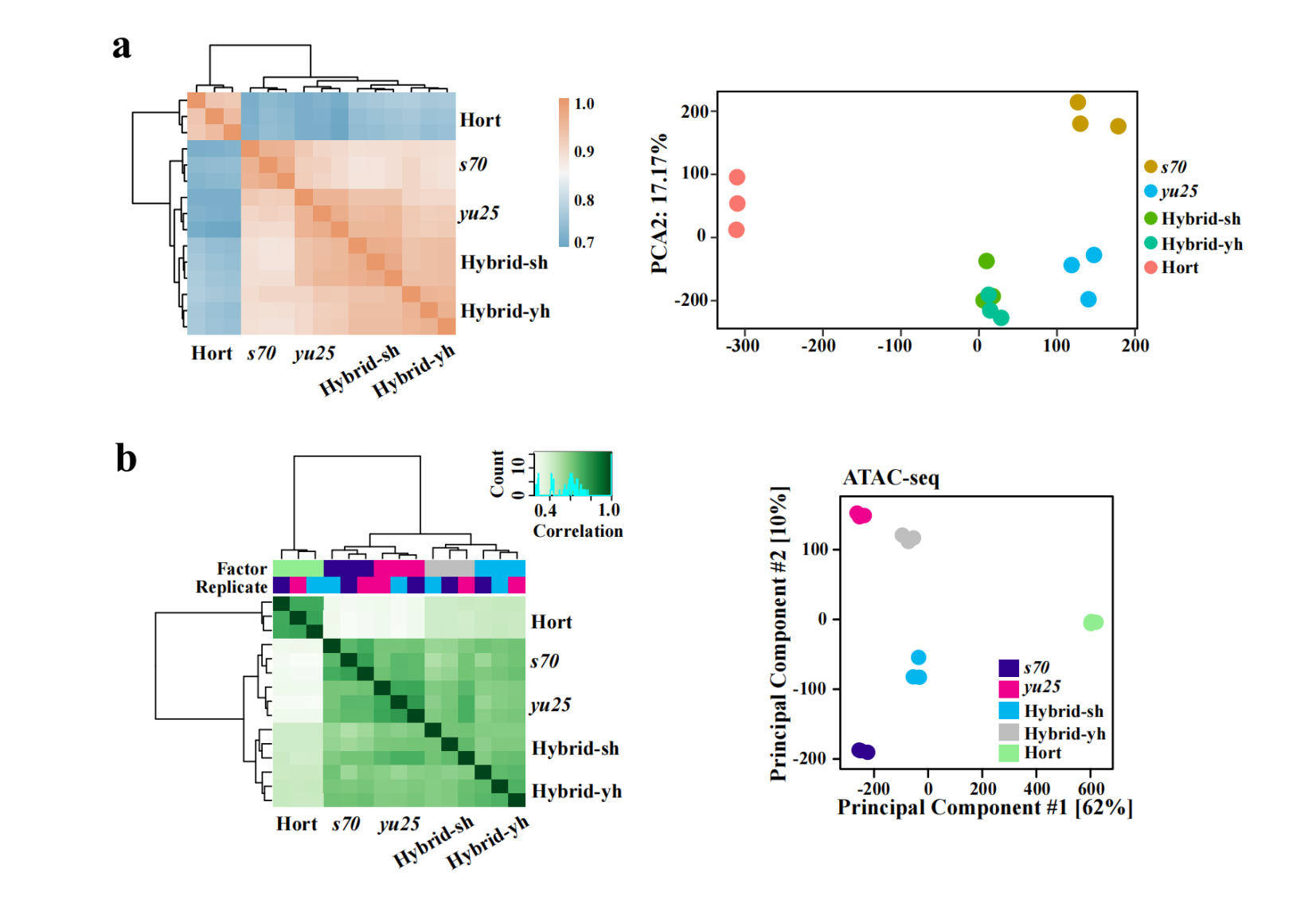
**

**Figure S2. Quality control of ATAC-seq and RNA-seq datasets. (a)** and **(b)** PCA plots (left) and Spearman's rank correlation coefficient heatmap (right) of RNA-seq **(a)** and ATAC-seq **(b)**.


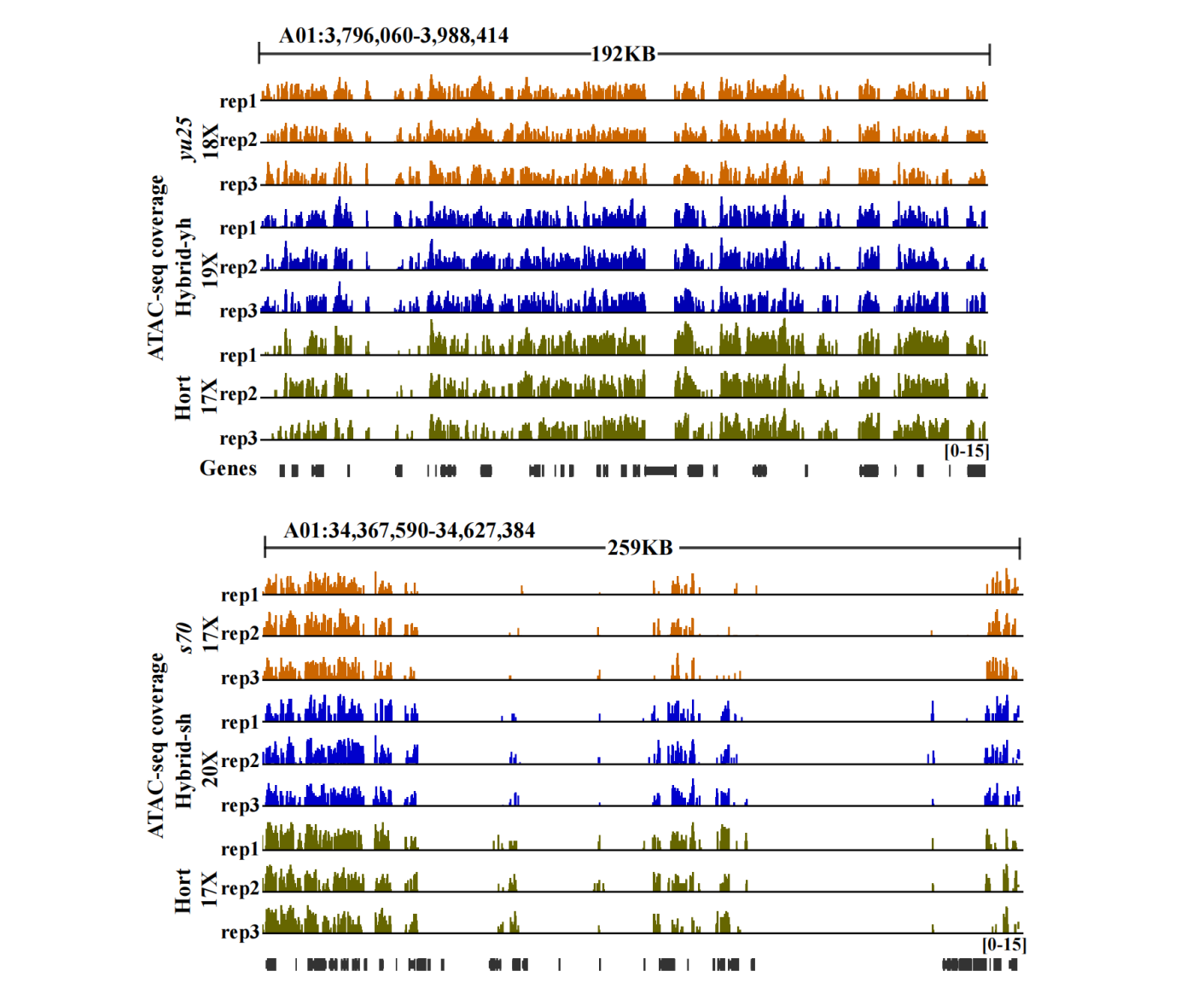


**Figure S3.** Genome browser showing ATAC-seq signals in the 192-kb and 259-kb region in two hybrids and their parents. Tracks show normalized values of three biological replicate for each sample.


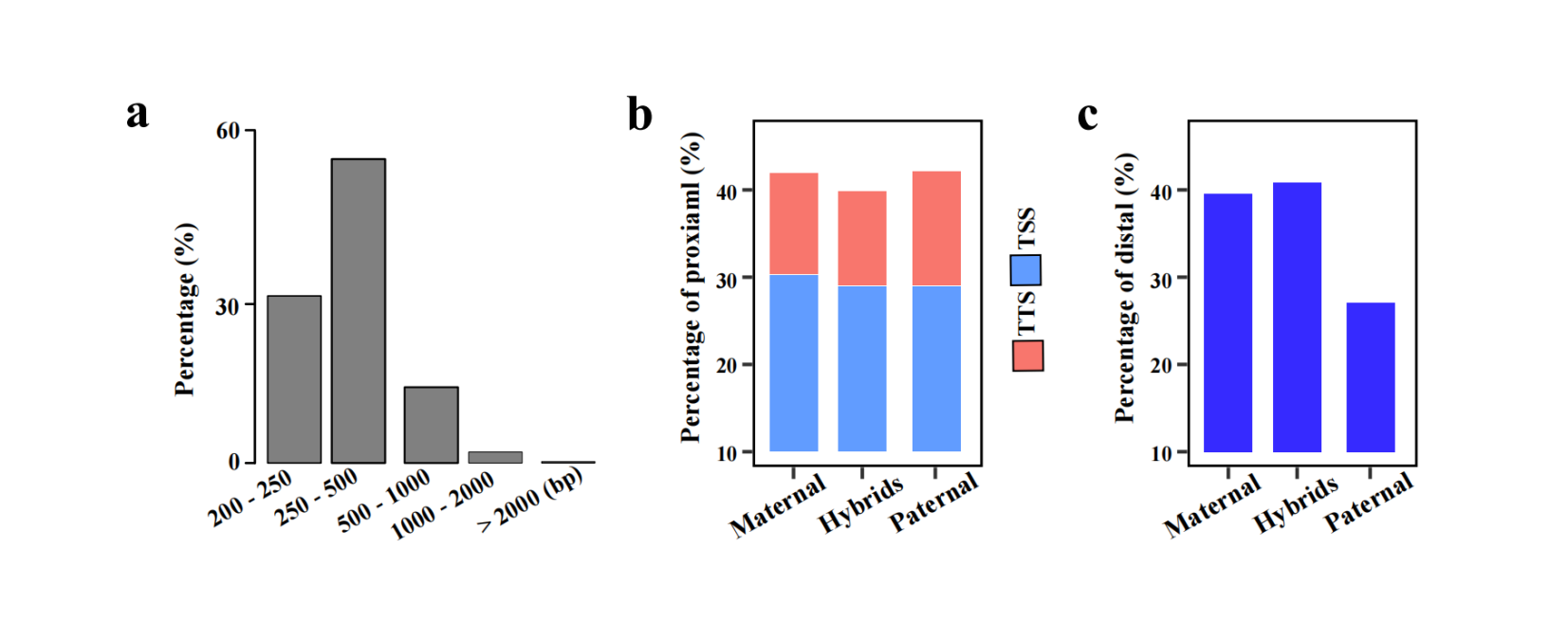


**Figure S4. Distribution of accessible chromatin regions (ACRs). (a)** The bar graph showed the percentage of ACRs with different lengths (bp). **(b)** and **(c)** The bar graph showed the percentage of proximal (including TSS and TTS) ACRs **(b)**, and distal ACRs **(c)** in F_1_ hybrids and their parents.


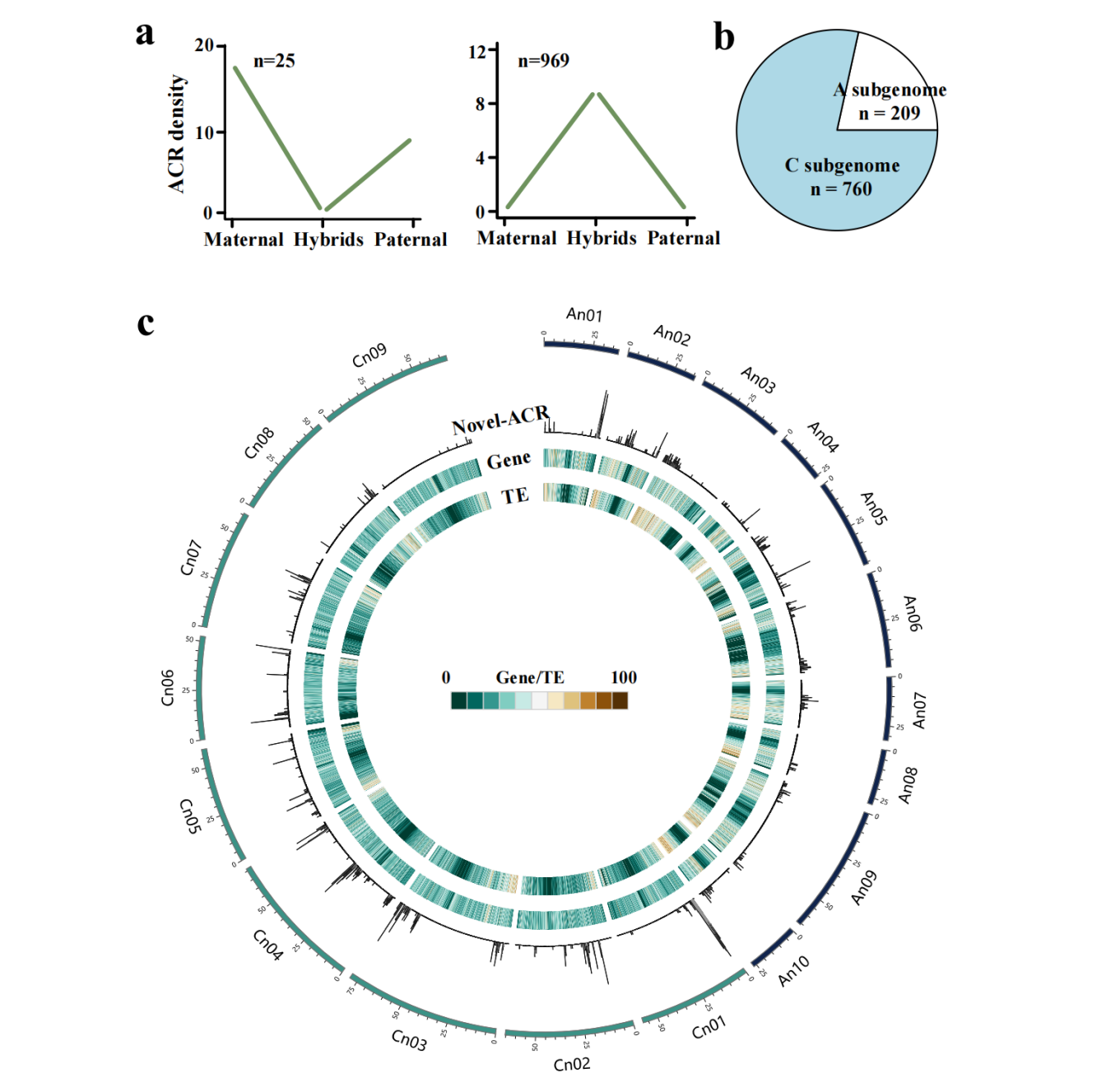


**Figure S5. Distribution of novel ACRs. (a)** The graph showed the ACR density of novel and silenced ACRs in F_1_ hybrids. The ACRs with Reads > 5 in F_1_ hybrids and Read = 0 in two parents were considered novel ACRs. The ACRs with Reads = 0 in F_1_ hybrids and reads > 5 in two parents were defined as silenced ACRs. **(b)** Number of novel ACRs in the A and C subgenomes in F_1_ hybrids. **(c)** Browser Circos plots showed the genome distribution of genes, TEs, and novel ACRs in F_1_ hybrids.


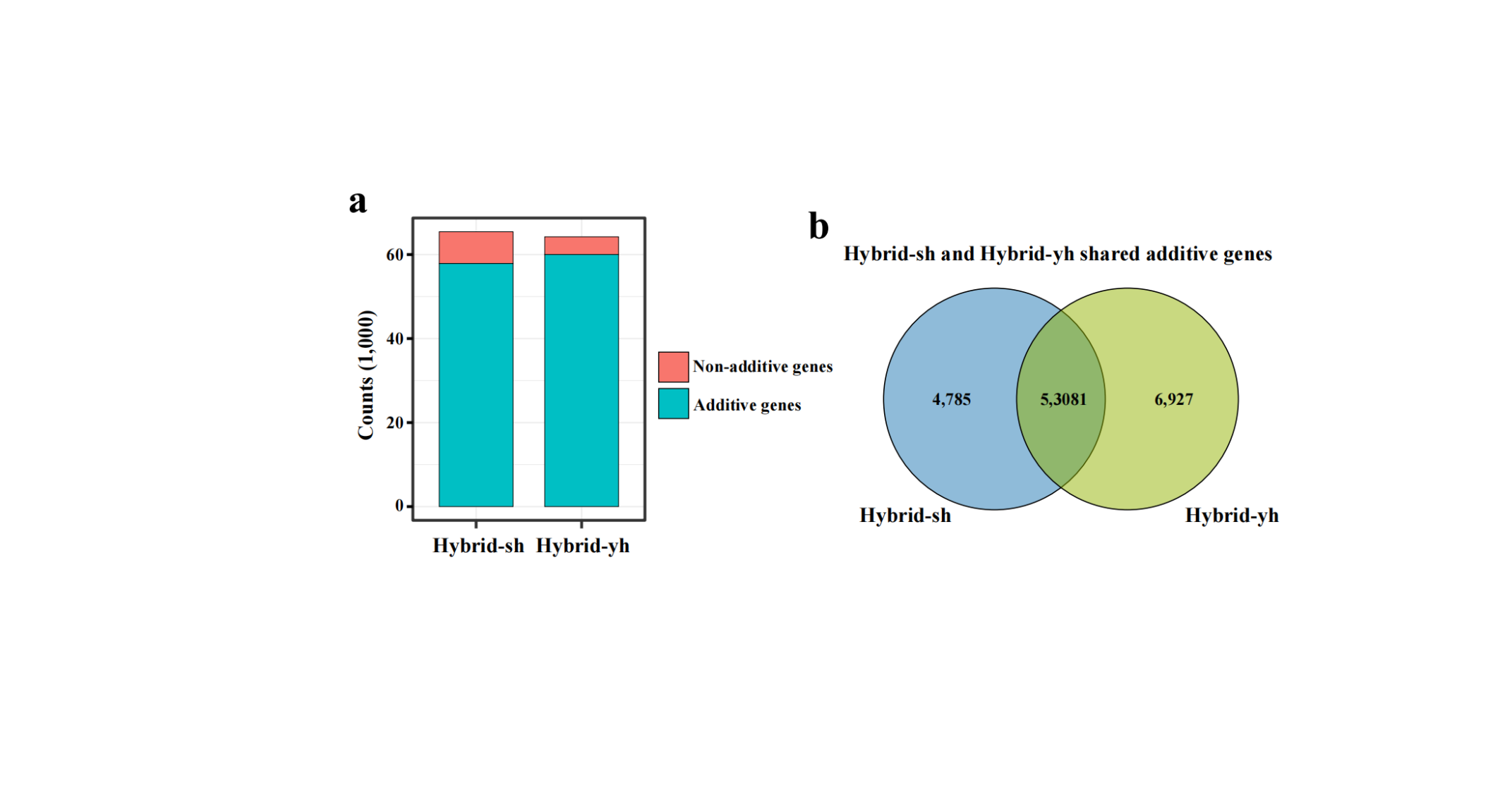


**Figure S6. Additive genes in two hybrids. (a)** The number of additive genes and non-additive genes in Hybrid-sh and Hybrid-yh. **(b)** Venn diagram showed the number of the overlapping additive genes in Hybrid-sh and Hybrid-yh.


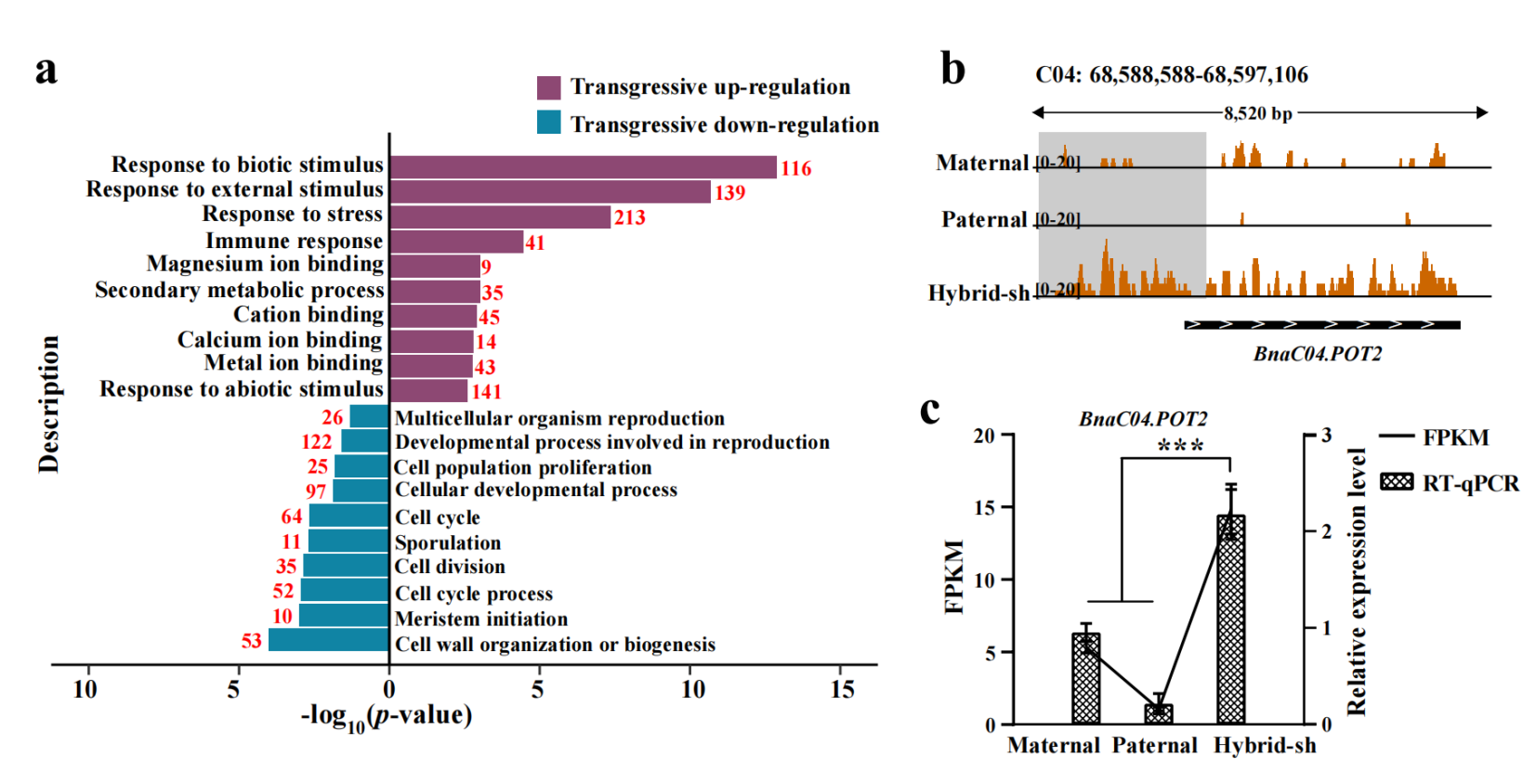


**Figure S7. GO enrichment of transgressive up-regulated genes in Hybrid-sh. (a)** GO enrichment analyses of transgressive regulation genes in Hybrid-sh. The top 10 biological processes GO terms were listed. **(b)** The genome browser showed ATAC-seq peaks around *Potassium Transporter 2* (*POT2*) in Hybrid-sh and its relative parents. **(c)** The graph showed the gene expression of *BnaC04.POT2* in Hybrid-sh and its relative parents. Error bars indicated the mean ± SD of three biological replicates. The student’s *t*-test; ****p* < 0.001.

**
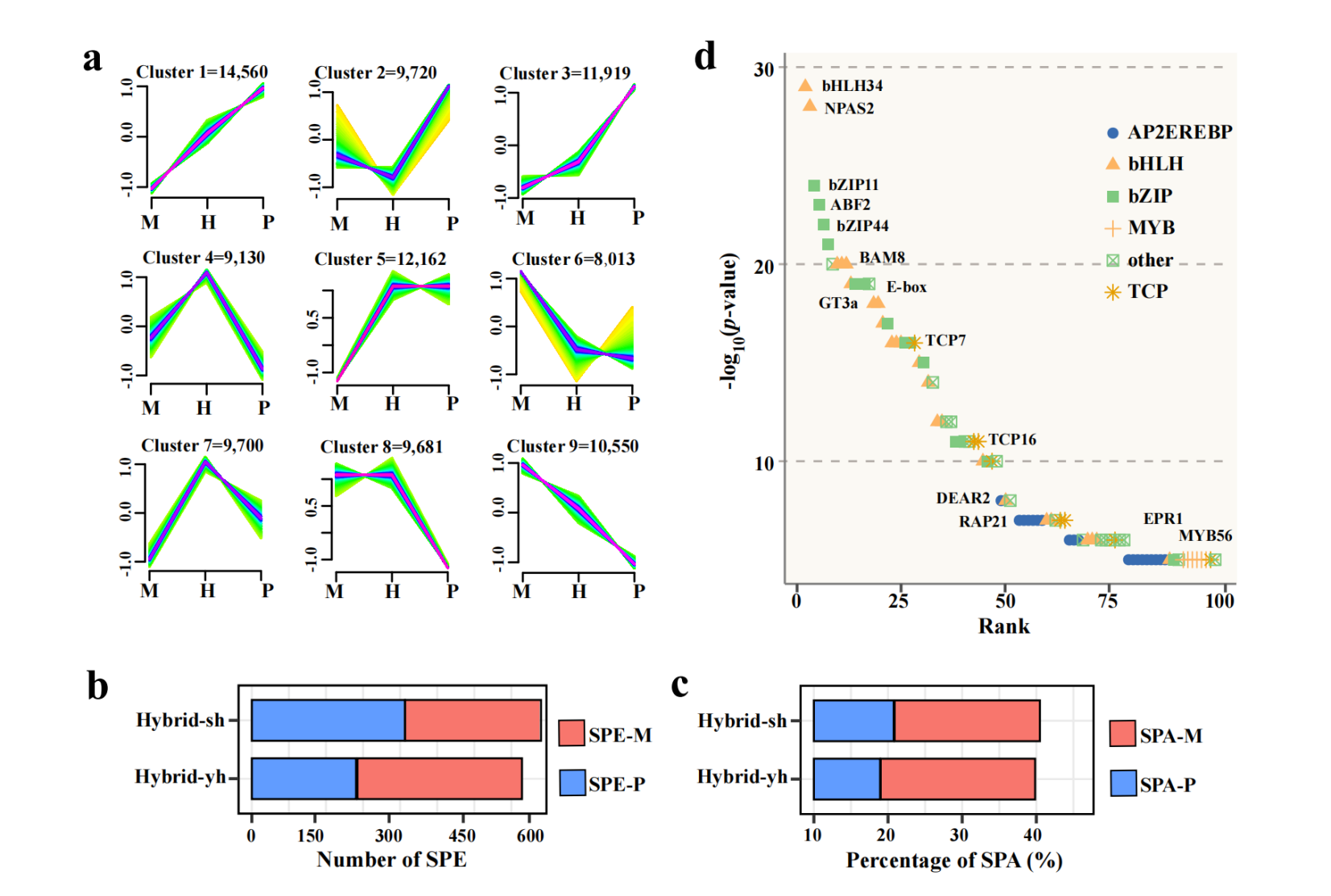
**

**Figure S8. Characterization of SPA-ACRs in F_1_ hybrids. (a)** The graphs showed the *c*-means soft clustering analysis of chromatin accessibility levels of A subgeomes in Hybrid-yh and its relative parents. **(b)** The graph showed the number of SPE-M and SPE-P genes in the A subgeomes of Hybrid-sh and Hybrid-yh. **(c)** The graph showed the percentage of SPA-M and SPA-P genes in the A subgeomes of Hybrid-sh and Hybrid-yh. **(d)** The graph showed the ranking of enriched motifs in Hybrid-sh ACRs. Colored dots represented transcription factors. The *p*-value for each subject was estimated using AME software.

**
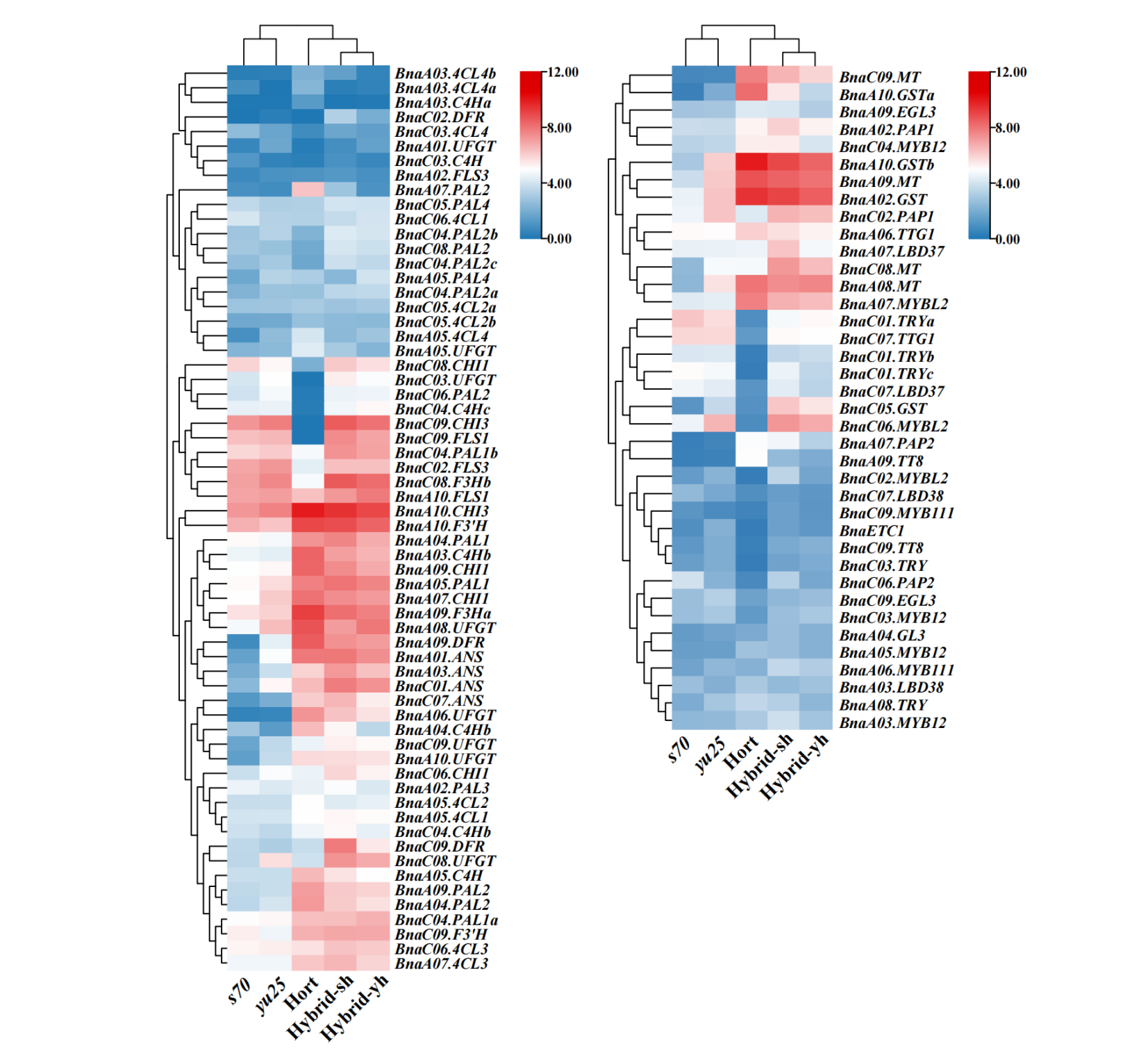
**

**Figure S9.** The heatmap showed the expression levels of anthocyanin biosynthesis genes and the related transcription factors in F_1_ hybrids and their parents. The colored boxes represent the gene expression heatmap, normalized by log_2_FPKM values obtained from RNA-seq.

**
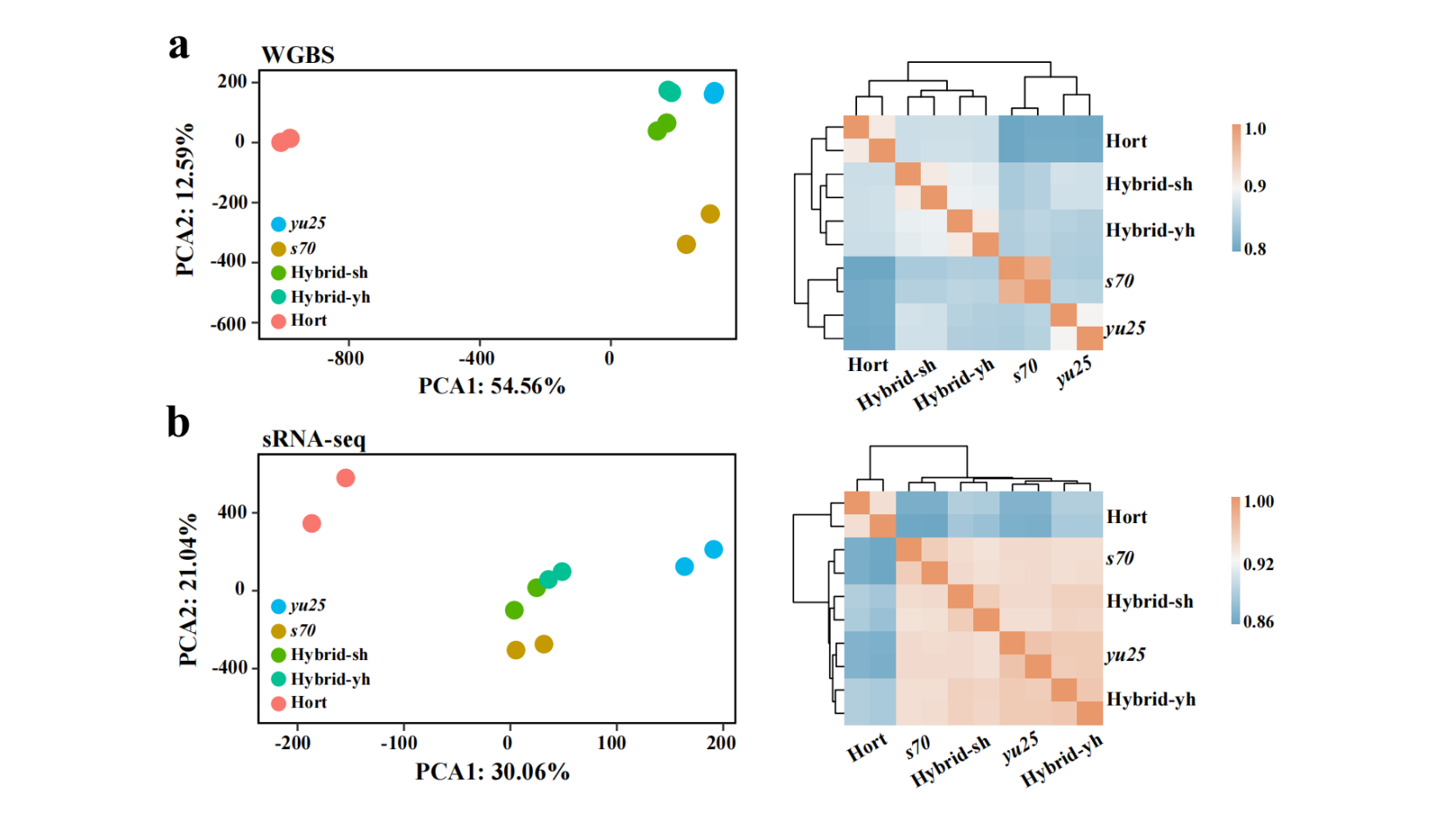
**

**Figure S10. Quality control of WGBS and sRNA-seq datasets.** The graphs showed the PCA plots (left) and Spearman's rank correlation coefficients (right) of WGBS **(a)** and sRNA-seq **(b)** datasets.


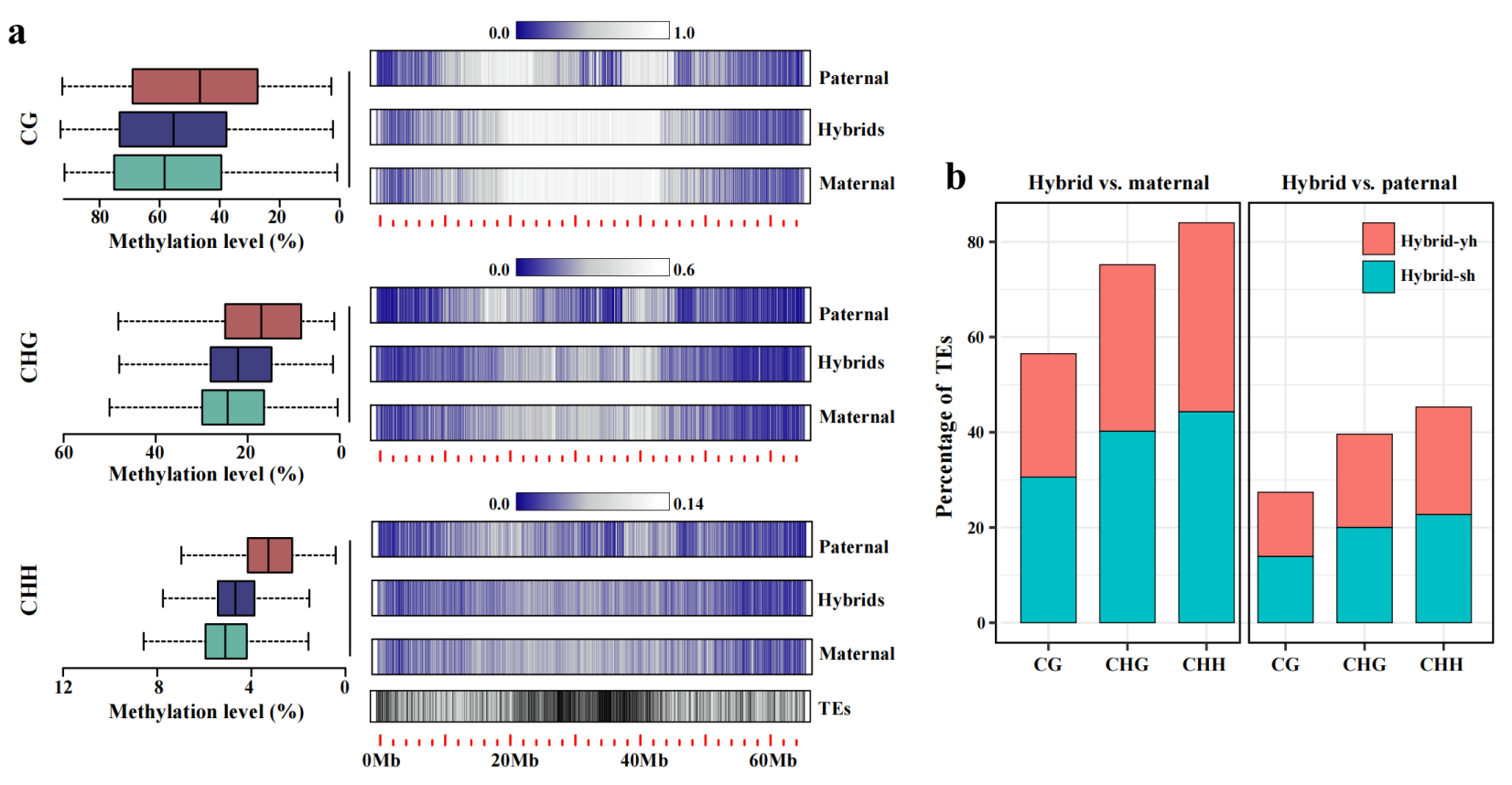
**Figure S11. Differences in methylation levels between F_1_ hybrids and their parental lines. (a)** Methylation levels of CG, CHG and CHH in F_1_ hybrids and their parental lines at the chromosome level. **(b)** Percentage of F_1_ hybrids with no significant difference in DNA methylation levels in transposable element (TE) bodies compared to maternal (left) and paternal (right) lines.

**
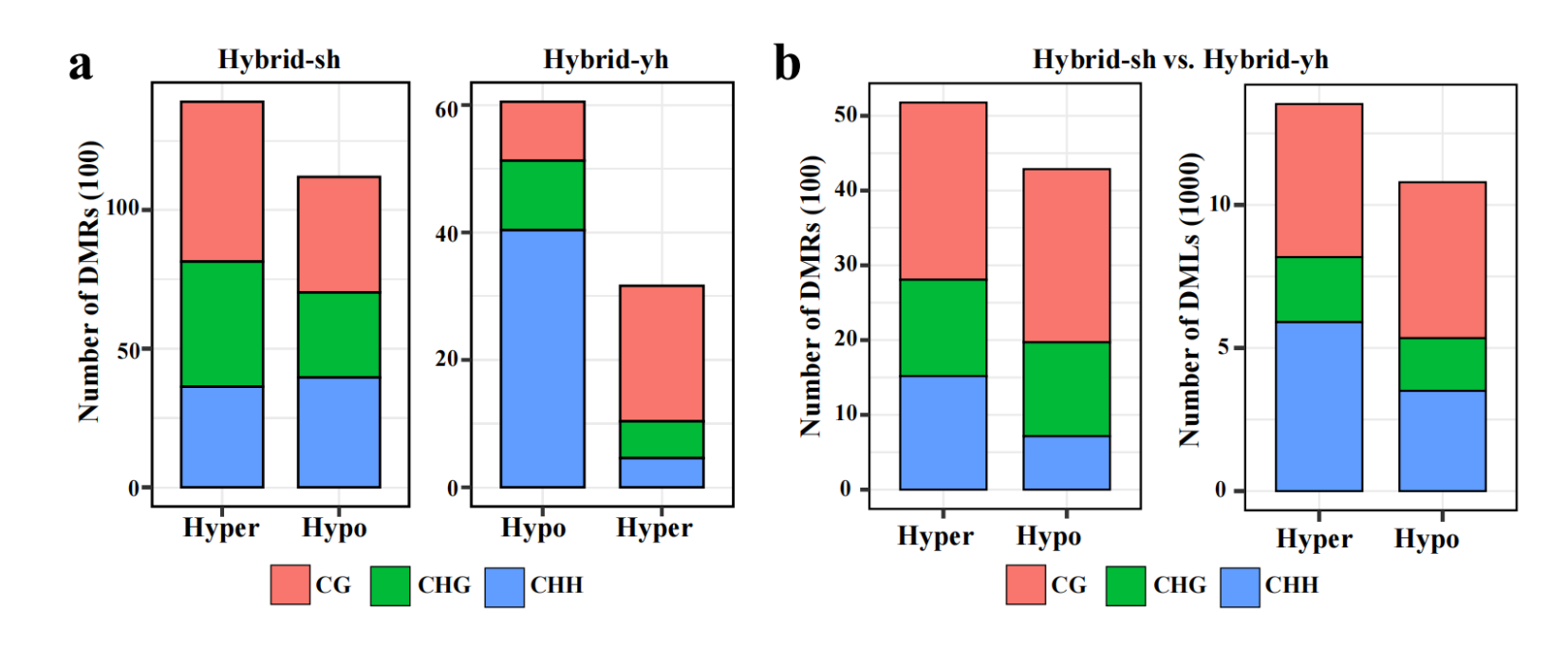
**

**Figure S12. Characterization of DMRs and DMLs in F_1_ hybrids.** **(a)** The bar graph showed the number of hyper- and hypo-DMRs in DNA methylation (CG, CHG, and CHH contexts) in Hybrid-sh and Hybrid-yh. **(b)** The number of differentially methylated regions (DMRs, right) and differential methylation locus (DMLs, left) between Hybrid-sh and Hybrid-yh.


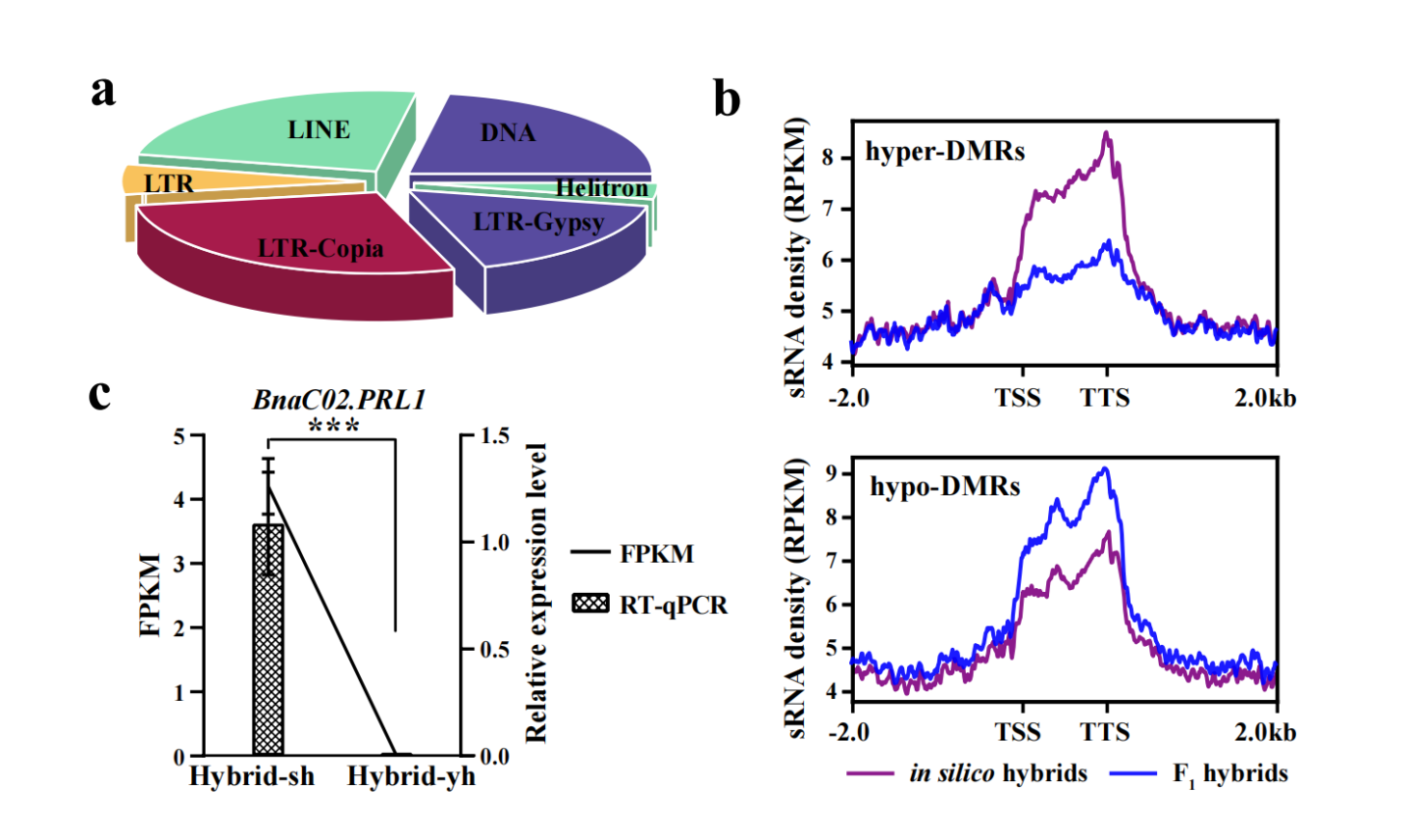


**Figure S13. The density of TEs and sRNAs in DMRs. (a)** The pie chart showed the proportion of TE-derived MDRs (TE-driven DMRs when more than 50% of the region overlaps with TEs). **(b)** The graph showed the sRNA densities of hyper-DMRs (top) and hypo-DMRs (bottom). **(c)** The graph showed the gene expression of *BnaC02.PRL1* in Hybrid-sh and Hybrid-yh. Error bars indicated the mean ± SD of three biological replicates. The student’s *t*-test; ****p* < 0.001.

**
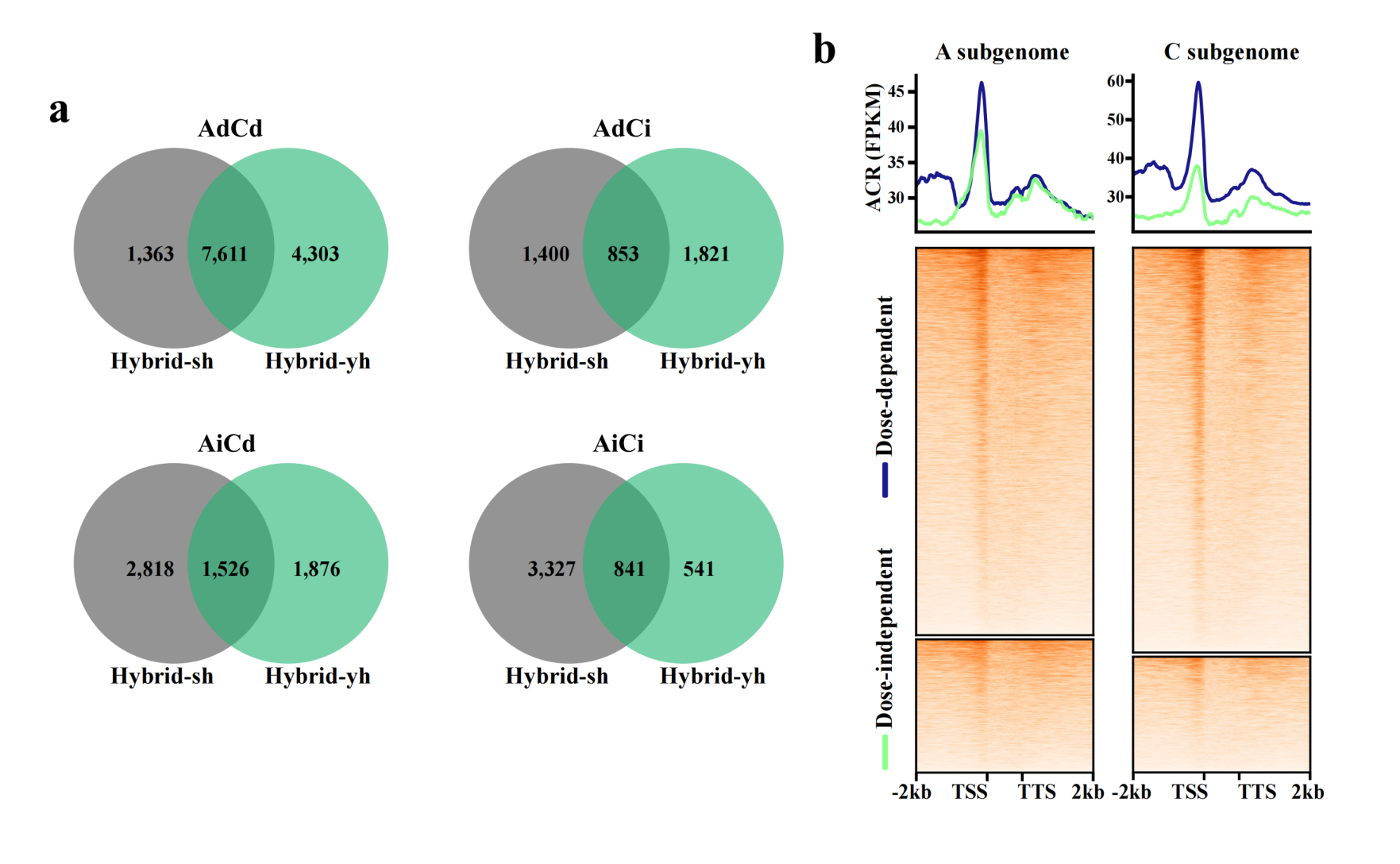
**

**Figure S14. Dose-dependent and dose-independent distributions in the A and C subgenomes. (a)** Venn diagram showed the number of the overlapping AdCd, AdCi, AiCd and AiCi genes in Hybrid-sh and Hybrid-yh. **(b)** The graph showed the dose-dependent and dose-independent ACR density distribution in the A and C subgenomes.

**Supplemental Table S1. Primer sequences used for RT-qPCR analyses.**

| **Gene_id** | **Forward primer (5'-3')** | **Reverse primer (5'-3')** |
| --- | --- | --- |
| *BnaA07.PAP2* | GCTGGTCTAAATCGGTGCAG | CGACCGGGTAATCTACCAGC |
| *BnaC02.PRL1* | GCTCTTGCATTCCCAGGTT | ACGATCCACTGCTTTTGCTAC |
| *BnaA06.UF3GT* | GGTTTTCTACGATTCCGCTGA | TTCTTCCTCACTAGAGGCGG |
| *BnaA01.POT4* | CTCAGCTTTCATGGGCGAG | AGCGTGAGGGTCCAGAAAATC |
| *BnaC04.POT2* | CAGGCAAGTCGCTGATGAG | ACAGCAGAGAACACGGAGATAG |
| *Actin7* | GGCATCACACTTTCTACAACGA | GCACAATACCAGTAGTACGTCC |

**Supplemental Table S2. Statistics of RNA-seq data and reads mapping for all samples.**

| **RNA-seq** | **Raw Reads** | **Clean Reads** | **Q30(%)** | **GC Content (%)** | **Mapped Clean reads** |
| --- | --- | --- | --- | --- | --- |
| **s70** | 20674100 | 19215459 | 93.65 | 47.31 | 1802602209 |
| **s70** | 20541951 | 19201504 | 93.82 | 47.23 | 1807821602 |
| **s70** | 21956773 | 20827026 | 93.57 | 46.45 | 1957115633 |
| **yu25** | 21721793 | 20540690 | 93.22 | 47.51 | 1937808695 |
| **yu25** | 21370948 | 20133806 | 93.05 | 47.2 | 1899423258 |
| **yu25** | 21011896 | 20546666 | 93.39 | 46.7 | 1955631670 |
| **Hybrid-sh** | 21487410 | 20941988 | 92.71 | 46.78 | 1984043943 |
| **Hybrid-sh** | 21434438 | 20589841 | 93.19 | 46.38 | 1964888527 |
| **Hybrid-sh** | 19786691 | 18711457 | 93.78 | 46.81 | 1765800197 |
| **Hybrid-yh** | 19760029 | 18592257 | 93.19 | 46.86 | 1764219267 |
| **Hybrid-yh** | 19900711 | 18869088 | 93.09 | 47.3 | 1782374052 |
| **Hybrid-yh** | 21251984 | 20003638 | 93.38 | 47.19 | 1898545283 |
| **hort** | 21862561 | 20822153 | 93.21 | 47.27 | 1850048294 |
| **hort** | 22985725 | 21883259 | 93.65 | 47.3 | 1946953553 |
| **hort** | 21643208 | 20421458 | 93.59 | 47.35 | 1810158037 |

**Supplemental Table S3. Statistics of ATAC-seq data and reads mapping for all samples.**

| **ATAC-seq** | **Raw Reads** | **Clean Reads** | **Q30(%)** | **SPOT** | **Mapped Clean reads** |
| --- | --- | --- | --- | --- | --- |
| **s70** | 46713984 | 46415584 | 94.46 | 0.28 | 41924249 |
| **s70** | 48946191 | 48635311 | 92.02 | 0.26 | 37994096 |
| **s70** | 50592226 | 50294108 | 92.2 | 0.29 | 39556686 |
| **yu25** | 51058619 | 50755263 | 92.48 | 0.28 | 38861401 |
| **yu25** | 49497213 | 49216199 | 92.46 | 0.26 | 38194516 |
| **yu25** | 48241753 | 47956390 | 92.01 | 0.28 | 37652044 |
| **Hybrid-sh** | 44186451 | 43851231 | 91.57 | 0.28 | 31213248 |
| **Hybrid-sh** | 50010924 | 49605541 | 92.27 | 0.28 | 38723274 |
| **Hybrid-sh** | 50513122 | 50120282 | 92.34 | 0.26 | 38399968 |
| **Hybrid-yh** | 45181181 | 44702269 | 92.31 | 0.25 | 33388099 |
| **Hybrid-yh** | 48940370 | 48488343 | 91.77 | 0.28 | 38499425 |
| **Hybrid-yh** | 41344897 | 41029470 | 92.07 | 0.25 | 29966350 |
| **hort** | 45155912 | 44639786 | 90.71 | 0.32 | 35038745 |
| **hort** | 48043231 | 47614365 | 91.28 | 0.32 | 36007670 |
| **hort** | 44909946 | 44517771 | 91.23 | 0.33 | 34211196 |

**Supplemental Table S4. GO enrichment analysis of conservative additive genes in two hybrids.**

| **GO_ID** | ***P*_value** | **Count** | **Description** |
| --- | --- | --- | --- |
| GO:0005488 | 1.11E-15 | 9178 | binding |
| GO:0003824 | 1.18E-05 | 10462 | catalytic activity |
| GO:0060090 | 3.39E-05 | 203 | molecular adaptor activity |
| GO:0045182 | 5.53E-05 | 113 | translation regulator activity |
| GO:0140104 | 3.66E-02 | 64 | molecular carrier activity |
| GO:0032991 | 0.00E+00 | 4951 | protein-containing complex |
| GO:0005622 | 0.00E+00 | 19927 | intracellular |
| GO:0009987 | 3.00E-15 | 19683 | cellular process |
| GO:0048511 | 1.69E-13 | 337 | rhythmic process |
| GO:0008152 | 3.83E-12 | 15897 | metabolic process |
| GO:0015976 | 1.19E-04 | 17 | carbon utilization |
| GO:0019740 | 4.46E-03 | 27 | nitrogen utilization |
| GO:0051179 | 1.24E-02 | 4417 | localization |

**Supplemental Table S5. GO enrichment analysis of non-additively up-regulated genes in Hybrid-sh.**

| **GO_ID** | ***P*_value** | **Count** | **Description** |
| --- | --- | --- | --- |
| GO:0009812 | 1.56E-04 | 23 | flavonoid metabolic process |
| GO:0009813 | 6.84E-04 | 18 | flavonoid biosynthetic process |
| GO:0051553 | 9.60E-04 | 7 | flavone biosynthetic process |
| GO:0009607 | 3.87E-14 | 176 | response to biotic stimulus |
| GO:0050896 | 5.67E-13 | 599 | response to stimulus |
| GO:0009605 | 4.64E-12 | 217 | response to external stimulus |
| GO:0009409 | 2.42E-10 | 82 | response to cold |
| GO:0009725 | 1.21E-07 | 220 | response to hormone |
| GO:0009719 | 2.18E-07 | 225 | response to endogenous stimulus |
| GO:0006629 | 8.45E-06 | 101 | lipid metabolic process |
| GO:0006631 | 3.11E-05 | 37 | fatty acid metabolic process |
| GO:0046283 | 1.02E-04 | 15 | anthocyanin-containing compound metabolic process |
| GO:0009733 | 1.29E-04 | 54 | response to auxin |
| GO:0044042 | 4.10E-04 | 29 | glucan metabolic process |
| GO:0051552 | 1.32E-03 | 7 | flavone metabolic process |

**Supplemental Table S6. GO enrichment analysis of non-additively up-regulated genes in Hybrid-yh.**

| **GO_ID** | ***P*_value** | **Count** | **Description** |
| --- | --- | --- | --- |
| GO:0042218 | 7.89E-08 | 5 | 1-aminocyclopropane-1-carboxylate biosynthetic process |
| GO:0010025 | 1.72E-05 | 6 | wax biosynthetic process |
| GO:1901570 | 3.56E-05 | 6 | fatty acid derivative biosynthetic process |
| GO:0009733 | 1.67E-03 | 15 | response to auxin |
| GO:0044255 | 2.94E-03 | 22 | cellular lipid metabolic process |
| GO:0072330 | 3.63E-03 | 11 | monocarboxylic acid biosynthetic process |
| GO:0009741 | 4.28E-03 | 7 | response to brassinosteroid |
| GO:0006629 | 4.80E-03 | 23 | lipid metabolic process |
| GO:0015698 | 8.35E-03 | 7 | inorganic anion transport |
| GO:0009812 | 1.46E-03 | 19 | flavonoid metabolic process |
| GO:0046283 | 4.14E-05 | 15 | anthocyanin biosynthesis process. |
| GO:0044042 | 3.16E-04 | 22 | glucan biosyntheti process |

**Supplemental Table S7. GO enrichment analysis of shared down-expressed genes in two hybrids.**

| **GO_ID** | ***P*_value** | **Count** | **Description** |
| --- | --- | --- | --- |
| GO:0010087 | 3.33E-16 | 54 | phloem or xylem histogenesis |
| GO:0010051 | 1.59E-09 | 27 | xylem and phloem pattern formation |
| GO:0009914 | 1.18E-08 | 34 | hormone transport |
| GO:0003018 | 2.13E-08 | 13 | vascular process in circulatory system |
| GO:0032350 | 3.67E-07 | 24 | regulation of hormone metabolic process |
| GO:0048316 | 9.91E-07 | 102 | seed development |
| GO:0042445 | 1.18E-06 | 57 | hormone metabolic process |
| GO:0010154 | 2.72E-06 | 104 | fruit development |
| GO:1905177 | 5.58E-06 | 9 | tracheary element differentiation |
| GO:0048367 | 9.76E-06 | 139 | shoot system development |
| GO:0072348 | 1.19E-05 | 14 | sulfur compound transport |
| GO:0052545 | 1.20E-05 | 15 | callose localization |
| GO:0062012 | 1.52E-05 | 28 | regulation of small molecule metabolic process |
| GO:0055085 | 2.55E-05 | 147 | transmembrane transport |
| GO:0000911 | 3.53E-05 | 17 | cytokinesis by cell plate formation |

**Supplemental Table S8. GO enrichment analysis of shared up-expressed genes in two hybrids.**

| **GO_ID** | ***P*_value** | **Count** | **Description** |
| --- | --- | --- | --- |
| GO:0010383 | 1.64E-04 | 6 | cell wall polysaccharide metabolic process |
| GO:0044036 | 3.68E-04 | 6 | cell wall macromolecule metabolic process |
| GO:0051707 | 4.51E-03 | 17 | response to other organism |
| GO:0043207 | 4.63E-03 | 17 | response to external biotic stimulus |
| GO:0009607 | 4.81E-03 | 17 | response to biotic stimulus |
| GO:0005976 | 5.42E-03 | 7 | polysaccharide metabolic process |
| GO:0009733 | 6.42E-03 | 8 | response to auxin |
| GO:0044419 | 7.07E-03 | 17 | interspecies interaction between organisms |
| GO:0044264 | 8.18E-03 | 6 | cellular polysaccharide metabolic process |
| GO:0071554 | 8.31E-03 | 9 | cell wall organization or biogenesis |
| GO:0006629 | 1.05E-02 | 12 | lipid metabolic process |
| GO:0044255 | 1.27E-02 | 11 | cellular lipid metabolic process |
| GO:0098542 | 1.43E-02 | 13 | defense response to other organism |
| GO:0009620 | 2.15E-02 | 7 | response to fungus |
| GO:0009605 | 2.64E-02 | 19 | response to external stimulus |

**Supplemental Table S9. The contents of 16 ionic components in F_1_ hybrids and their parents.**

|  | **s70** | **yu25** | **Hybrid**  **-sh** | **Hybrid**  **-yh** | **hort** | **Mean** | **SD^1^** | **CV^1^** |
| --- | --- | --- | --- | --- | --- | --- | --- | --- |
| Mg^2^ | 2.61 | 2.96 | 4.79 | 2.77 | 2.09 | 3.04 | 1.03 | 33.84 |
| P | 7.86 | 6.05 | 10.58 | 8.42 | 5.04 | 7.59 | 2.16 | 28.41 |
| K | 22.01 | 27.98 | 45.25 | 36.07 | 34.23 | 30.91 | 8.00 | 25.89 |
| Ca | 8.04 | 6.99 | 14.77 | 9.66 | 6.29 | 9.15 | 3.39 | 37.04 |
| Na | 1.88 | 1.95 | 1.27 | 1.14 | 1.67 | 1.58 | 0.36 | 22.83 |
| B^2^ | 31.22 | 39.41 | 28.57 | 25.91 | 32.47 | 31.52 | 5.09 | 16.14 |
| Se | 0.10 | 0.09 | 0.11 | 0.10 | 0.07 | 0.09 | 0.02 | 17.21 |
| Mn | 20.15 | 20.02 | 21.80 | 19.63 | 18.13 | 19.95 | 1.31 | 6.58 |
| Fe | 46.69 | 51.37 | 46.50 | 46.51 | 62.83 | 50.78 | 7.05 | 13.89 |
| Cu | 3.09 | 3.45 | 3.66 | 3.74 | 5.11 | 3.81 | 0.77 | 20.25 |
| Zn | 49.65 | 53.53 | 43.91 | 41.98 | 47.25 | 47.26 | 4.59 | 9.7 |
| Mo | 0.30 | 0.38 | 0.35 | 0.36 | 0.49 | 0.37 | 0.07 | 18.21 |
| Ba | 10.80 | 11.75 | 7.67 | 8.03 | 9.54 | 9.56 | 1.75 | 18.3 |
| As | 0.03 | 0.05 | 0.03 | 0.02 | 0.01 | 0.03 | 0.02 | 59.47 |
| Cd | 0.05 | 0.08 | 0.12 | 0.11 | 0.26 | 0.12 | 0.08 | 64.14 |
| Pb | 0.01 | 0.01 | 0.01 | 0.01 | 0.01 | 0.01 | 0.00 | 15.12 |

1. The parameters shown are: standard deviation (± SD from five replicates), Coefficient of Variation (CV, %).
2. The units of 5 macroelements (Mg, P, K, Ca, and Na) are (g·kg-1), and the units of 11 trace elements (B, Se, Mn, Fe, Cu, Zn, Mo, Ba, As, Cd, and Pb) are (mg·kg-1).

**Supplemental Table S10. Statistics of WGBS-seq data and reads mapping for all samples.**

| **WGBS** | **QC-passed reads** | **Properly paired reads** | **Average Cytosine depth** | **Bisulfite conversion (%)** | **mC/(C+T) (%)** |
| --- | --- | --- | --- | --- | --- |
| **s70** | 203755516 | 192649161 | 30.3139 | 99.58 | 10.61 |
| **s70** | 242642051 | 196800471 | 33.6186 | 99.21 | 13.29 |
| **yu25** | 203750557 | 193562163 | 30.2654 | 99.32 | 14.47 |
| **yu25** | 193436940 | 184328420 | 28.8405 | 99.45 | 12.95 |
| **Hybrid-sh** | 199423059 | 188154639 | 29.6604 | 99.26 | 12.52 |
| **Hybrid-sh** | 206212634 | 195152714 | 30.6538 | 99.38 | 10.26 |
| **Hybrid-yh** | 184643639 | 173473362 | 27.416 | 99.41 | 12.03 |
| **Hybrid-yh** | 207849273 | 197326844 | 30.8683 | 99.55 | 11.03 |
| **hort** | 193809351 | 177983477 | 28.5439 | 99.16 | 9.85 |
| **hort** | 238282559 | 219909569 | 35.0476 | 99.23 | 10.42 |

**Supplemental Table S11. Statistics of sRNA-seq data and reads mapping for all samples.**

| **sRNA-seq** | **Raw Reads** | **Clean Reads** | **Q20 (%)** | **Q30 (%)** | **Mapped Clean reads** |
| --- | --- | --- | --- | --- | --- |
| **s70** | 29806536 | 28640685 | 99.3 | 97.7 | 26041698 |
| **s70** | 28599807 | 27126278 | 99.1 | 97.1 | 26482857 |
| **yu25** | 29752067 | 28267018 | 99.1 | 96.9 | 27275545 |
| **yu25** | 30151921 | 28854786 | 98.2 | 95.1 | 27904231 |
| **Hybrid-sh** | 29158016 | 27039422 | 98.4 | 95.6 | 26166560 |
| **Hybrid-sh** | 28095774 | 27315576 | 98.6 | 95.9 | 26116061 |
| **Hybrid-yh** | 28393134 | 27749028 | 98.5 | 95.7 | 26825736 |
| **Hybrid-yh** | 28179167 | 27519684 | 98.2 | 95 | 26567866 |
| **hort** | 28700923 | 28020472 | 98.9 | 96.5 | 26911249 |
| **hort** | 28403184 | 27835112 | 99.1 | 97.1 | 26416568 |
