# Supplementary material for: Interspecific hybridization in Brassica species leads to changes in agronomic traits through the regulation of gene expression by chromatin accessibility and DNA methylation
Source: Gigascience. 2025 Apr 22;14:giaf029. doi: 10.1093/gigascience/giaf029 (PMC12012897; doi:10.1093/gigascience/giaf029)
Supplement: giaf029_GIGA-D-24-00365_Revision_1 [file giaf029_giga-d-24-00365_revision_1.pdf]

GigaScience

Interspecific hybridization in Brassica species leads to changes in agronomic traits through the regulation of gene expression by chromatin accessibility and DNA methylation

--Manuscript Draft--

**Manuscript Number:**	GIGA-D-24-00365R1
**Full Title:**	Interspecific hybridization in Brassica species leads to changes in agronomic traits through the regulation of gene expression by chromatin accessibility and DNA methylation
**Article Type:**	Research
**Funding Information:**	Science and Technology Innovation 2030-Major Project (2023ZD04068) — Dr. Cheng Dai
**Abstract:**	Interspecific hybridization is a common method in plant breeding to combine traits from different species, resulting in allopolyploidization and significant genetic and epigenetic changes. However, our understanding of genome-wide chromatin and gene expression dynamics during allopolyploidization remains limited. This study generated two Brassica allotriploid hybrids via interspecific hybridization. We investigated accessible chromatin regions (ACRs), and DNA methylation regulates gene expression after interspecific hybridization, ultimately influencing the agronomic traits of the hybrids. 234,649 ACRs were identified in the parental lines and hybrids; the hybridization process induces changes in the distribution and abundance of their accessible chromatin regions, particularly in gene regions and their proximity. Genes associated with Proximal ACRs were more highly expressed than those associated with Distal and Genic ACRs. More than half of novel ACRs drove transgressive gene expression in the hybrids, and the transgressive up-regulated genes showed significant enrichment in metal ion binding, especially magnesium ion, calcium ion, and potassium ion binding. We also identified the Bna.bZIP11 in the single-parent activation ACR (SPA-ACR), which binds to BnaA06.UF3GT to promote anthocyanin accumulation in F1 hybrids. DNA methylation plays a role in repressing gene expression, and unmethylated ACRs are more transcriptionally active. Additionally, the A-subgenome ACRs were associated with genome dosage rather than DNA methylation. The interplay among DNA methylation, TEs, and sRNA contributes to the dynamic landscape of ACRs during interspecific hybridization, resulting in distinct gene expression patterns on the genome.
**Corresponding Author:**	Cheng Dai<br>Huazhong Agricultural University College of Plant Science and Technology<br>Wuhan, Hubei CHINA
**Corresponding Author Secondary Information:**	
**Corresponding Author's Institution:**	Huazhong Agricultural University College of Plant Science and Technology
**Corresponding Author's Secondary Institution:**	
**First Author:**	Chengtao Quan
**First Author Secondary Information:**	
**Order of Authors:**	Chengtao Quan
	Qin Zhang
	Xiaoni Zhang
	Kexin Chai
	Guoting Cheng
	Chaozhi Ma

Powered by Editorial Manager® and ProduXion Manager® from Aries Systems Corporation

	Cheng Dai
**Order of Authors Secondary Information:**	
**Response to Reviewers:**	Dear GigaScience Editor,<br><br>We would like to thank you and the reviewers for your constructive feedback on our manuscript. Your insightful comments and guidelines for revision have been invaluable in improving the quality of our work. We now submit the revised manuscript titled “Interspecific hybridization in Brassica species leads to changes in agronomic traits through the regulation of gene expression by chromatin accessibility and DNA methylation (GIGA-D-24-00365)” by Quan et al. In response to the main comments and suggestions, we have made several significant improvements, providing more robust and quantitative evidence for our findings. We have also clarified our explanations and refined the language throughout.<br>1. Introduction and Discussion Revision: We have revised the background and discussion section with recent advances in accessible chromatin regions (ACRs), DNA methylation, and its role in gene regulation in species of the genus Brassica or other plants.<br>2. Data availability: We calculate the sequencing depth of ATAC-seq and demonstrate that the data has good reproducibility and high quality through IGV visualization.<br>3. Revision of Results: The Results section has been updated to include the differences in DNA methylation and ACR that arise from interspecific hybridization in the two hybrids. Additionally, we analyzed the distribution and shifts of ACRs in both the A and C subgenomes. The discussion also addresses the reasons for these observed differences.<br>In summary, we have clarified the results in the manuscript and discussed the relevant findings to address the important concerns and criticisms raised by the reviewers and the editor. Specific responses to each reviewer are also provided below. Major changes and improvements in the revised manuscript are highlighted in blue. We hope our revised manuscript can now be considered for publication in GigaScience.<br>Thank you for considering our submission.<br>With best wishes<br>Cheng Dai<br><br><br>Reviewer reports:<br><br>Reviewer #1: The manuscript presents a wealth of data on chromatin accessibility and DNA methylation during polyploidization, offering fresh insights into these processes in the context of interspecific hybridization. While the study provides valuable observations, several key issues must be addressed to strengthen the conclusions.<br>Major Points:<br>1. Data Quality Concerns: In Fig 1B, the Hort dataset appears to be of lower quality compared to the other datasets, which may affect some conclusions. Similarly, Figure 2d shows low enrichment levels, indicating potential issues with data quality. This could impact conclusions regarding ACR distribution and differences before and after allopolyploidization. The authors should carefully reconsider the conclusions drawn, especially regarding ACR calling.<br>Response: Thanks for your suggestions! The ATAC-seq peaks were enriched in the following samples: 192,786 in s70, 155,900 in yu25, 137,384 in Hybrid-sh, 135,728 in Hybrid-yh, and 82,149 in Hort (new Fig. 1b). The maternal lines (s70 and yu25) are the allotetraploid Brassica napus (AACC, 2n=38), the paternal line (Hort) is the diploid Brassica rapa (AA, 2n=20), and the F1 hybrid is an allotriploid hybrid (Hybrid-sh and Hybrid-yh; AAC, 2n=29). The ACR peaks in Hort were less than maternal lines and F1 hybrids. This could be due to the Hort lacking the C subgenome. We then compared the ACR peaks in different subgenomes in F1 hybrids and parental lines. The result showed that 71,312, 56,146, 70,413, 68,466, and 82,149 ACR peaks were identified in the A subgenome of s70, yu25, Hybrid-sh, Hybrid-yh, and Hort, respectively (new Fig. 1b). However, in the C subgenome, 121,474, 99,754, 66,971, and 67,262 ACR peaks were identified in s70, yu25, Hybrid-sh, and Hybrid-yh (new Fig. 1b), indicating that the ACR densities in the C-subgenome are suppressed by hybridization. We have revised the results by including the ACR peaks in different subgenomes. Please find the relative data in new Figure 1b, and the results in lines 212-223. To minimize the impact on the data from the reduction of ACR peaks caused by the deletion of the C

subgenome in Hort, we analyzed the distribution of ACRs and identified novel ACRs following interspecific hybridization by separately comparing the A and C subgenomes. For single-parent activation ACRs, we concentrated solely on the peaks associated with the A subgenome.
There are two main categories within non-additive genes: expression level dominance (ELD) and transgressive (Yoo et al. 2014). Based on gene expression level, the transgressive genes could be further categorized as transgressive up-regulated genes and transgressive down-regulated genes (Yoo et al. 2014). We have identified 3,163 and 645 transgressive expression genes in Hybrid-sh and Hybrid-yh, respectively (new Fig. 3c). In Hybrid-yh, the number of down-regulated genes (464) was significantly higher than the number of up-regulated genes (181) (new Fig. 3c). In comparison, no difference was observed between the number of up-regulated (1,503) and down-regulated (1,660) transgressive genes in Hybrid-sh (new Fig. 3c). The ACR densities of transgressive up-regulated genes in F1 hybrids were higher than parental lines (new Fig. 3d). In contrast, ACR densities of transgressive down-regulated genes in F1 hybrids were significantly lower than parental lines (new Fig. 3d). These results indicate that a significant correlation between the expression of transgressive genes and ACRs. We have revised Figure 3 and the results in lines 319-327.

2. Read Distribution in Parent Tracks: In Fig 2G, the two parent tracks seem to have no detectable reads. The authors should verify the sequencing depth and confirm whether it is sufficient for reliable data interpretation.
Response: Thanks for your suggestions! We have verified the average sequencing depth of ATAC-seq by samtools (depth -b). The results showed that the average sequencing depth of chromatin accessibility regions for s70, yu25, Hybrid-sh, Hybrid-yh, and Hort were 17, 18, 20, 19, and 17X values, respectively (new Fig.S3). The genome browser results showed that consistent ACR peaks were identified in three biological replicates of the parental line and F1 hybrids (new Fig.S3). We identified 969 novel ACRs and 25 silent ACRs in F1 hybrids (new Fig. S5a). Among them, novel ACRs in the A and C subgenomes were 209 and 760 (new Fig. S5b), respectively, indicating that the formation of novel ACRs in the C subgenome was more significant than in the A subgenome. For instance, in a window with a range of 7,657 bp, a novel ACR was identified in Hybrid-sh (new Fig. 3f). These results indicate that interspecific hybridization may lead to the activation of ACR in unopened regions (Wang et al. 2023; Han et al. 2022). We have revised Figures S3, S5, and Figure 3, and the results are in lines 212-215, 270-285, and 295-347.

3. Distribution and Shift of ACRs in the A and C Subgenomes: The authors conclude that interspecific hybridization induces changes in ACR distribution and abundance, especially in gene and proximal regions. However, it would be crucial to clarify how these changes differ between the A and C subgenomes. Are these changes biased toward one subgenome, or are they converging? The authors should compare their findings with studies such as the referenced paper on cotton hybrids (Ref. 36) and discuss the similarities and differences.
Response: Thanks for your suggestions! The parental lines s70, yu25, and Hort exhibited 23,770, 18,715, and 27,382 ACRs in the A subgenomes (new Fig. 2b). In contrast, 40,755 and 33,220 ACRs were identified in the C subgenomes of s70 and yu25, respectively (new Fig. 2b), showing the divergent distribution of ACRs between the A subgenome and the C subgenome. Significant differences in ACR distribution between subgenomes A and D were also found in G. hirsutum (AADD) (Han et al. 2022). We further analyzed whether there is immediate convergence in the distribution of different types of ACRs across subgenomes in interspecific hybrids. We further analyzed whether there is immediate convergence in the distribution of different types of ACRs (genic, proximal, and distal ACRs) across subgenomes in interspecific hybrids. There was no difference in genic ACR between the F1 hybrid and its parental A subgenome; however, significant differences were observed between proximal and distal ACRs (new Fig. 2b). This suggests that genic ACRs in the A subgenome converge immediately following interspecific hybridization. Notably, in the A subgenome, proximal ACRs were most abundant in the maternal line, followed by F1 hybrids, while the paternal line exhibited the least abundance (new Fig. 2b); in contrast, distal ACRs show the opposite trend (paternal > F1 hybrids > maternal) (new Fig. 2b). However, no significant differences were found in the proportions of the genic, proximal, and distal ACR in the C subgenome (new Fig. 2b), suggesting that the different types of ACRs converge immediately upon the halving of the C subgenome

following interspecific hybridization. We have revised the new Figure 2; the results are in lines 248-268.

Minor Points:
1. The chromosome number diagram in Figure 1a lacks a scale bar, which should be included for reference.
Response: Corrected! Please find the new Fig. 1a

2. In Fig 2F, it would be useful to show the proportions of various TE classes across the genome for better context.
Response: Corrected! Please find the new Fig. 2d

3. The order of Hort distribution in Figure 2b should be made consistent for better comparison.
Response: Corrected! Please find the new Fig. 2a

4. The number of additively expressed genes is mentioned, but further explanation is needed regarding how these genes were identified and their biological significance.
Response: Thanks for your suggestions! To identify additive and nonadditive gene expression, we constructed independently in silico 'hybrids' by combining the RNA-seq data from sequenced parental individuals in a 1:1 ratio for the B. napus (s70 and yu25) and B. rapa (Hort) datasets. This ratio reflects the respective 1:2 genomic contribution of the parents to the F1 hybrids. The differentially expressed genes (DEGs) were identified by comparing the expression levels of genes between the hybrids and in silico hybrids, using the criteria of an adjusted p-value < 0.05 and a $|\log 2 \text{ fold change}| \geq 1.5$. The results showed that the majority of expressed genes exhibited additive expression patterns in both Hybrid-sh (82.9%) and Hybrid-yh (90.1%), and more than 86% of the additive genes were conserved in the two hybrids (new Fig. 3a). GO enrichment analysis of conservative additive genes revealed enrichment for cellular process, rhythmic process, metabolic process, carbon-nitrogen utilization (new Table S4). This suggests that conserved additive genes are necessary for normal plant growth and development. We have revised the materials & methods and results sections in lines 164-171 and 295-318, respectively.

5. Clarify whether the enriched genes in Hybrid-yh are non-additively up-regulated, as the connection to gene regulation is unclear.
Response: Thanks for your suggestions! We identified 7,567 and 4,129 non-additively genes in Hybrid-sh and Hybrid-yh, respectively. In Hybrid-sh, there were 2,508 non-additively up-regulated genes and 5,058 non-additively down-regulated genes. In contrast, Hybrid-yh exhibited 482 non-additively up-regulated genes and 3,646 non-additively down-regulated genes. The non-additively up-regulated genes in Hybrid-sh were mainly associated with responses to biotic and abiotic stimuli, immune responses, and metabolic processes (new Table S5). Conversely, non-additively up-regulated genes in Hybrid-yh were enriched in ion transport, response to hormones, and metabolic processes (new Table S6). GO enrichment of non-additively up-regulated genes in F1 hybrids suggests an enhanced capacity for metabolite production and improved tolerance to environmental conditions. We have revised the new Figure 3; the results are in lines 294-318.

6. Replace "enrichment" with "GO enrichment" to ensure clarity in the text.
Response: Corrected!

7. The section title needs revision, as much of the content focuses on gene expression rather than chromatin changes.
Response: Thanks for your suggestions! We have revised the section title to “Nonadditive gene expression in F1 hybrids”.

8. The authors should cite relevant literature supporting the impact of LTRs and LINEs on gene regulation.
Response: Thanks for your suggestions! LTRs contribute to the silencing of active elements, affecting transposition potential and siRNA targeting levels and ultimately influencing the function of the element in the genome (Bousios et al. 2016). LTRs and LINE TEs in the < 2 kb promoter region of the gene decreased gene body chromatin accessibility and gene expression (Jordan et al. 2020). We have cited the relative

research papers.

9. Provide more information on the distribution of novel ACRs and their overlap with TEs. It would be important to discuss whether this overlap influences gene expression.
Response: Thanks for your suggestions! It is a great idea to analyze the association between novel ACRs and their overlap with TEs. We identified 969 novel ACRs and 25 silent ACRs in F1 hybrids (new Fig. S5a). Among them, novel ACRs in the A and C subgenomes were 209 and 760, respectively (new Fig. S5b), indicating that the formation of novel ACRs in the C subgenome was more significant compared to the A subgenome. The distribution of novel ACRs revealed that the proportions of ACRs in the genic, proximal, and distal were 35.7%, 32.4%, and 32.9%, respectively (new Fig. 2c). Furthermore, the chromosomal distribution of novel ACRs was mainly located at the telomeres, distal to the centromere (new Fig. S5c). 375 (38.7%) of these novel ACRs were classified as TE-driven ACRs, with LTR/Gypsy-type retrotransposons playing a prominent role (30.4%) compared to other types of TEs (new Fig. 2d). We found that gene expression in the proximal regions of non-TE-mediated ACRs was significantly higher than that of TE-mediated ACRs (new Fig. 2e). Transposable element (TE)-mediated ACRs may exhibit lower gene expression levels due to the influence of methylation on both the TE and its adjacent sequences. In contrast, non-TE-mediated ACRs are typically unaffected by transposable elements; consequently, DNA methylation levels in these regions may be reduced, thereby facilitating the expression of genes in neighboring areas. We have revised the results and discussions in lines 270-285.

10. The conclusion that hybrids tend to inherit specific sequences from the parent with higher methylation needs further explanation. How was this determined?
Response: Thanks for your suggestions! At the chromosomal level, a bias in the total methylation levels of CG, CHG, and CHH towards the hypermethylated parent was observed (new Fig. S11a). Further, the methylation levels of transposable element (TE) bodies in F1 hybrids and their parental lines across the whole genome were analyzed. We identified 25.9-30.6%, 35.0-40.2%, and 39.7-44.3% TEs in F1 hybrids CG, CHG, and CHH contexts, respectively, which were not significantly different from the maternal line (s70 and yu25) (new Fig. S11b). Compared with the paternal line (Hort), the proportions of TE no differences in CG, CHG, and CHH contexts that were observed in F1 hybrids were 13.5-13.9%, 19.6-20.0%, and 22.6-22.7% (new Fig. S11b). This indicates that the F1 hybrid tended to inherit higher methylation levels from the parent with higher methylation. We have revised the new Figure S11; the results are in lines 409-418.

11. The threshold used for dose-dependent and independent gene identification lacks a clear explanation. Please provide details on how these thresholds were determined.
Response: Thanks for your suggestions! The identification of dosage-dependent and independent genes was followed in the previous study (Shi et al. 2015). Briefly, Pearson correlation tests, employing the Benjamini and Hochberg false discovery rate (FDR) method, were conducted to assess the relationship between gene expression and genotype dosage for the A-subgenome (1/2 : 2/3 : 1) and C-subgenome (1/2 : 1/3 : 0) genes. The Pearson correlation and multiple test corrections were performed using the adjustment method in R. Genes exhibiting a significant correlation between expression and dosage ($R2 > 0.64$, $FDR < 0.05$) were classified as dosage-dependent. In contrast, those with no significant correlation were deemed dosage-independent. For instance, the Pearson correlation coefficient for genomic dosage (1/2 : 2/3 : 1) and gene expression values (1.72, 1.96, 2.54) of the gene BnaA01G0002000ZS is 0.99, with an R2 of 0.99, indicating that this gene is dosage-dependent. We have also revised the materials and methods section in lines 172-182.

Overall, this study is rich in data and offers valuable insights into epigenetic regulation during hybridization. However, addressing these issues will help to clarify the findings and improve the manuscript's impact.
Reviewer #2: This study investigates the construction of two hybrids through the crossing of two Brassica napus cultivars (s70, AsAsCsCs; yu25, AyAyCyCy, allotetraploid) with a Brassica rapa cultivar (B. campestris L. ssp. chinensis var. purpuria Hort.; AhAh, diploid). The research systematically explores accessible chromatin regions (ACRs), DNA methylation, and gene expression. Notable differences in the expression of non-additive genes and up-regulated transgressive

genes associated with metal ion accumulation, as well as SPE genes linked to anthocyanin accumulation, were observed. Furthermore, the study reveals the role of ACRs and DNA methylation in regulating gene expression, including the impact of dosage effects. Before the article is accepted, the following revisions are recommended

1. The introduction is overly brief and lacks sufficient background information. It should include recent progress on accessible chromatin regions (ACRs), DNA methylation, and their role in gene regulation, especially in Brassica species or other crops. A clearer description of the research objectives and their potential implications would enhance the introduction.
Response: Thanks for your suggestions! We have revised the background section.

2. The differential expression of non-additive genes between Hybrid-sh and Hybrid-yh is reported, with insights from GO-enrichment analysis. However, the authors should focus more on the shared gene expression patterns and provide a more in-depth discussion of how interspecies hybridization induces gene expression changes via ACRs and DNA methylation.
Response: Thanks for your suggestions! The 2,530 identical non-additive expressed genes (2,313 down-regulated and 217 up-regulated) were isolated in both Hybrid-sh and Hybrid-yh (new Fig. 3b). These down-regulated genes were associated with primary meristem tissue development, organic acid transmembrane transport, phloem transport, and auxin polar transport by GO enrichment analysis (new Table S7). In contrast, the up-regulated genes were enriched with response to external biotic stimulus, lipid metabolic process, and defense response pathways (new Table S8). We have revised the results in lines 312-318 and updated the discussion in lines 597-614.

3. The differences in DNA methylation and ACR patterns between the two hybrids (Hybrid-sh and Hybrid-yh) induced by interspecies hybridization need to be more clearly presented in the figures and text. Additionally, the authors should offer a detailed discussion of the reasons behind these differences.
Response: Thank you for your suggestions! We have analyzed differentially expressed accessible chromatin regions (DE-ACRs) in the two hybrids, identifying 1,478 DE-ACRs in Hybrid-yh compared to Hybrid-sh. Among these, 766 were differentially up-regulated ACRs, while 712 were differentially down-regulated (new Fig. 2f). Notably, the number of DE-ACRs in the C-subgenome (855) was significantly higher than that in the A-subgenome (593), illustrating a positive correlation between the number of DE-ACRs and genome size (new Fig. 2f). We further found that proximal DE-ACRs were the most numerous, followed by genic DE-ACRs, with distal DE-ACRs being the least prevalent. This indicates an uneven distribution of DE-ACRs across the genome (new Fig. 2g). Additionally, we identified 9,464 differentially methylated regions (DMRs) between Hybrid-sh and Hybrid-yh. In the context of Hybrid-sh, we recorded 2,370, 1,289, and 1,518 hyper-DMRs, along with 2,317, 1,256, and 714 hypo-DMRs in the CG, CHG, and CHH contexts, respectively (new Fig. S12b). Furthermore, regarding differential methylation loci (DMLs), we observed 5,323, 2,271, and 5,902 hyper-DMLs, as well as 5,448, 1,840, and 3,503 hypo-DMLs in the CG, CHG, and CHH contexts respectively, when compared to Hybrid-sh (new Fig. S12b). The differences in the number of hyper-DMRs (hyper-DMLs) and hypo-DMRs (hypo-DMLs) between the two hybrids stemmed from the DMRs (DMLs) in the CHH context. This data highlights the unbalanced changes in the levels of hyper- and hypo-CHH methylation between the two hybrids. We have updated the results in lines 286-293 and 437-444, as well as the discussion in lines 580-591.

4. In the section on "Accessible Chromatin Regions and DNA Methylation Differ in Genome Dosage Effects," the authors should provide a more thorough discussion in relation to previous studies on Brassica napus or other polyploid crops. This would help contextualize the findings and demonstrate how they align with or differ from existing knowledge.
Response: Thank you for your suggestions! In comparative studies examining gene expression patterns within polyploid plant species, it has been observed that most genes across different subgenomes exhibit coordinated expression, either in a dosage-dependent or dosage-independent manner (Shi et al. 2015; Tan et al. 2016). In the resynthesized allotetraploid Arabidopsis, approximately 56% of the alleles displayed congruent dosage expression patterns (Shi et al. 2015). Similar trends were noted in

	synthetic Brassica napus and its derivatives, where 58% of the A and C subgenome genes dosage expressed in the same direction (Tan et al. 2016). Our study on homologous genes from the A and C subgenomes in a related context further supports these findings, revealing that 66% of the genes exhibited coherent dosage expression, with 54% showing dosage-dependent behavior (AdCd) and 12% exhibiting dosage-independent behavior (AiCi) (new Fig. 6d). These results highlight the prevalence of both coordinated and independent regulatory mechanisms that influence gene expression in polyploid genomes. This underscores the importance of a nuanced understanding of the genetic and epigenetic factors that govern these expression patterns. We have updated the discussion in lines 540-560.<br><br>5. The discussion section currently lacks sufficient detail and does not adequately compare the results with previous studies. It would benefit from a deeper analysis of the novel insights provided by this study, as well as a discussion of any differences between the two hybrids.<br>Response: Thanks for your suggestions! We have revised the whole discussion section.<br><br>References<br>Shi X, Zhang C, Ko DK, Chen ZJ. Genome-wide dosage-dependent and -independent regulation contributes to gene expression and evolutionary novelty in plant polyploids. Mol Biol Evol 2015;32(9): 2351-66.<br>Tan C, Pan Q, Cui C, Xiang Y, Ge X, Li Z. Genome-Wide Gene/Genome Dosage Imbalance Regulates Gene Expressions in Synthetic Brassica napus and Derivatives (AC, AAC, CCA, CCAA). Front Plant Sci 2016;7:1432.<br>Han M, Sun Q, Zhou J, Qiu H, Guo J, Lu L, et al. Insertion of a solo LTR retrotransposon associates with spur mutations in 'Red Delicious' apple (Malus × domestica). Plant Cell Rep 2017;36(9):1375-1385.<br>Wang P, Gu M, Yu X, Shao S, Du J, Wang Y, et al. Allele-specific expression and chromatin accessibility contribute to heterosis in tea plants (Camellia sinensis). Plant J 2023;112(5):1194-1211.<br>Bousios A, Diez CM, Takuno S, Bystry V, Darzentas N, Gaut BS. A role for palindromic structures in the cis-region of maize Sirevirus LTRs in transposable element evolution and host epigenetic response. Genome Res 2016;26(2):226-37.<br>Jordan KW, He F, de Soto MF, Akhunova A, Akhunov E. Differential chromatin accessibility landscape reveals structural and functional features of the allopolyploid wheat chromosomes. Genome Biol 2020;21(1):176.<br>Han J, Lopez-Arredondo D, Yu G, Wang Y, Wang B, Wall SB, et al. Genome-wide chromatin accessibility evolution during polyploidization in cotton. Proc Natl Acad Sci U S A 2022;119(44):e2209743119.<br>Yoo MJ, Liu X, Pires JC, Soltis PS, Soltis DE. Nonadditive gene expression in polyploids. Annu Rev Genet 2014;48: 485-517.
**Additional Information:**	
**Question**	**Response**
Are you submitting this manuscript to a special series or article collection?	No
**Experimental design and statistics**<br><br>Full details of the experimental design and statistical methods used should be given in the Methods section, as detailed in our Minimum Standards Reporting Checklist. Information essential to interpreting the data presented should be made available in the figure legends.	Yes

Have you included all the information requested in your manuscript?	
**Resources**<br><br>A description of all resources used, including antibodies, cell lines, animals and software tools, with enough information to allow them to be uniquely identified, should be included in the Methods section. Authors are strongly encouraged to cite Research Resource Identifiers (RRIDs) for antibodies, model organisms and tools, where possible.<br><br>Have you included the information requested as detailed in our Minimum Standards Reporting Checklist?	Yes
**Availability of data and materials**<br><br>All datasets and code on which the conclusions of the paper rely must be either included in your submission or deposited in publicly available repositories (where available and ethically appropriate), referencing such data using a unique identifier in the references and in the "Availability of Data and Materials" section of your manuscript.<br><br>Have you have met the above requirement as detailed in our Minimum Standards Reporting Checklist?	Yes

Powered by Editorial Manager® and ProduXion Manager® from Aries Systems Corporation

Manuscript Click here to access/download;Manuscript;Mauscript-GigaScience_Mar 3.docx

Title Page

Interspecific hybridization in Brassica species leads to changes in agronomic traits through the regulation of gene expression by chromatin accessibility and DNA methylation

Chengtao Quan[1,2], Qin Zhang[1,2], Xiaoni Zhang[1,2], Kexin Chai[1,2], Guoting Cheng[3], Chaozhi Ma[1,2], and Cheng Dai[1,2]

1 National Key Laboratory of Crop Genetic Improvement, Huazhong Agricultural University, Wuhan 430070, China

2 Hubei Hongshan Laboratory, Wuhan, 430070, China

3 College of Informatics, Huazhong Agricultural University, Wuhan 430070, China.

The author responsible for the distribution of materials integral to the findings presented in this article following the policy described in the Instructions for Authors is:

Cheng Dai (cdai@mail.hzau.edu.cn)

To whom correspondence should be addressed.

Dr. Cheng Dai

National Key Laboratory of Crop Genetic Improvement, Huazhong Agricultural University, Wuhan 430070, P.R. China

Email: cdai@mail.hzau.edu.cn

ORCIDs: Cheng Dai [0000-0002-4853-8278]

Abstract

Interspecific hybridization is a common method in plant breeding to combine traits from different species, resulting in allopolyploidization and significant genetic and epigenetic changes. However, our understanding of genome-wide chromatin and gene expression dynamics during allopolyploidization remains limited. This study generated two *Brassica* allotriploid hybrids via interspecific hybridization. We investigated accessible chromatin regions (ACRs), and DNA methylation regulates gene expression after interspecific hybridization, ultimately influencing the agronomic traits of the hybrids. 234,649 ACRs were identified in the parental lines and hybrids; the hybridization process induces changes in the distribution and abundance of their accessible chromatin regions, particularly in gene regions and their proximity. Genes associated with Proximal ACRs were more highly expressed than those associated with Distal and Genic ACRs. More than half of novel ACRs drove transgressive gene expression in the hybrids, and the transgressive up-regulated genes showed significant enrichment in metal ion binding, especially magnesium ion, calcium ion, and potassium ion binding. We also identified the *Bna.bZIP11* in the single-parent activation ACR (SPA-ACR), which binds to *BnaA06.UF3GT* to promote anthocyanin accumulation in F_1 hybrids. DNA methylation plays a role in repressing gene expression, and unmethylated ACRs are more transcriptionally active. Additionally, the A-subgenome ACRs were associated with genome dosage rather than DNA methylation. The interplay among DNA methylation, TEs, and sRNA contributes to the dynamic landscape of ACRs during interspecific hybridization, resulting in distinct gene expression patterns on the genome.

Keywords: chromatin accessibility; hybridization; DNA methylation; transposable elements; transgressive

Background

Interspecific hybridization is an essential tool in plant breeding and genetic improvement. It allows the incorporation of desirable traits from different species into a single organism, improving crop quality and productivity [1-4]. This process has revolutionized crop breeding and contributed significantly to the global food supply [5,6]. A major advantage of interspecific hybridization is the generation of hybrids with superior vigor compared to their parents. This vigor arises from interactions between alleles at multiple loci, epistatic interactions, and possibly complementation of deleterious mutations [7]. Emerging evidence suggests epigenetic factors are crucial for hybrid vigor [2,8]. However, the genetic mechanisms underlying interspecific hybridization are complex and poorly understood.

Accessible chromatin regions (ACRs) are typically nucleosome-free or loosely bound to nucleosomes, making them more susceptible to binding by regulatory proteins that affect gene expression. In plant genomes, chromatin's open state directly influences gene expression regulation during essential biological processes such as cell differentiation, growth, and development [9-11]. Pinpointing ACRs is critical to understanding *cis*-regulatory elements (CREs) in the genome, which form the intricate transcriptional regulatory networks that control gene expression [12]. For instance, auxin has been shown to quickly rewire the totipotency network involved in somatic embryogenesis in *Arabidopsis* by altering chromatin accessibility [13]. Further investigation shows that the B3-type transcription factor LEC2 promotes the formation of somatic embryos by directly activating the early embryonic patterning genes *WOX2* and *WOX3* [13]. In *Arabidopsis*, the long-day condition induces a greater number of ACRs in the leaf epidermis and vascular companion cell; additionally, compared to the leaf epidermis cell, more ACRs were identified in the vascular companion cells, which are situated further away from the gene region [14]. ACRs at different locations in the genome can be categorized as genic, proximal, and distal [10]. Genic ACRs are usually located within genes, such as exons and introns, which affect the conduct of transcription and the processing of mRNA [15]. Therefore, the maintenance and monitoring of genic ACRs is essential to maintain the integrity and stability of the genome [16]. Proximal ACRs are typically located near critical promoter regions that bind transcription factors and other regulatory proteins, directly influencing the expression of nearby genes [12-13]. Several binding sites for flowering-related transcription factors were identified in proximal ACRs induced by long-day in *Arabidopsis*, and *TREHALOSE PHOSPHATASE/SYNTHASE 9* (*TPS9*)

was identified as a flowering activator [14]. Distal ACRs contain long-range acting regulatory elements, such as enhancers or silencers, essential for regulating complex gene expression patterns [14]. Despite their long physical distance from target genes, they can interact with the promoter regions of genes through multiple mechanisms (e.g., chromatin looping and folding) to influence gene expression [17,18]. For instance, distal ACRs can be modified by H3K56 acetylation (H3K56ac), which may function as enhancers, while H3K27 trimethylation (H3K27me3) may act as repressors [19]. Changes in these ACRs can result in cell-specific alterations in expression patterns and biological functions [13,14,19]. In summary, ACRs provide valuable insights into how the physical structure of chromatin affects gene expression. Thus, unraveling these ACRs and their association with gene expression profiles may help to elucidate the complex regulatory network behind hybrid vigor.

DNA methylation is a vital process of epigenetic modification in which methyl groups are added to the DNA molecule. It is critical in regulating gene expression without affecting the genetic sequence [20,21]. Most DNA methylation patterns in plants are generally stable, but DNA methylation in CHG and CHH contexts can be altered following interspecific hybridization [22]. Dynamic DNA methylation variation was observed during the development of hybrid rice, with many of the DMRs (differentially methylated regions) of the parental species retained in hybrids, but only a few of the DMRs exhibited non-additive variation, and these were not significantly correlated with changes in gene expression [23]. Changes in DNA methylation levels in *Brassica napus* do not adequately explain subgenomic dominance [24]. However, the interaction between DNA methylation and accessible chromatin regions may be the key to differential gene expression [25,26]. In *Osmanthus fragrans*, the expression of *CCD4*, a key gene for ionone synthesis, was correlated with the gene's promoter region exhibiting different methylation levels and chromatin accessibility [25]. Genome-wide chromatin accessibility profiles in 18 deletion mutants of *Arabidopsis* with CG, CHG, or CHH DNA methylation revealed that DNA methylation affects chromatin accessibility in all three sequence contexts [26]. Most chromatin accessibility regions are hypomethylated, and acclimation-induced changes in DNA methylation can influence the expression of proximal and distal genes [27-29].

Brassica napus (NCBI:txid3708; AACC; 2n = 4x = 38) is generated by hybridizing from *Brassica rapa* (AA; 2n = 2x = 20) and *Brassica oleracea* (CC; 2n = 2x = 18) [30]. DNA methylation patterns and ACRs in *B. napus*, shaped by breeding and natural selection, are linked to its adaptive and

agronomic traits [31-34]. Methylation changes between the globular embryo and mature green stages and leaves show that most promoter methylation is established early in seed development and remains stable [31]. DNA methylation also plays a key role in enhancing hybrid vigor in early seed and seedling growth [32]. It helps reduce transposable elements activity during hybridization by affecting DNA methylation levels through non-additively expressed siRNA clusters [33]. Furthermore, the C subgenome has been found to have greater chromatin accessibility than the A subgenome, mainly due to the unique genes in the C subgenome, not shared genes [34]. This indicates a complex relationship between epigenetic regulation and the traits of *B. napus*.

Interspecific hybridization between *Brassica napus* and *Brassica rapa* effectively broadens the genetic base of *B. napus*. This method takes advantage of the significant potential of natural triploids in polyploid breeding [35,36]. Additionally, these allotriploid hybrids provide a unique opportunity to explore the effects of polyploidization on global gene expression, allowing for precise comparisons between F_1 hybrids and their diploid or tetraploid progenitors [37-39]. Previous research on interspecific hybrids of *B. napus* and *B. rapa* has primarily focused on genomic structural variations and differences in gene expression [35,36,40-43]. However, the potential mechanisms by which interspecific hybridization induces differences in gene expression leading to phenotypic changes in hybrids have not been extensively explored. We obtained a series of allotriploid hybrids (AAC, 2n = 29) by crossing *B. rapa* with *B. napus*. Two F_1 hybrids exhibiting distinct characteristic biases were selected for RNA-seq, sRNA-seq, ATAC-seq, and WGBS to comprehensively evaluate the relationship between phenotypic changes induced by gene expression differences after interspecific hybridization and DNA methylation and accessible chromatin regions. We reveal that novel ACRs drove the transgressive up-regulated genes in hybrids and that transgressive up-regulated genes were significantly enriched for metal ion binding. Additionally, DNA methylation plays a role in repressing gene expression within ACRs, and unmethylated ACRs are more transcriptionally active. This information can be used to develop breeding programs to improve crop performance.

Materials and Methods

Plant material

Two *Brassica napus* cultivars (s*70*, $A_sA_sC_sC_s$; *yu25*, $A_yA_yC_yC_y$, allotetraploid) were selected as the maternal parent, and *Brassica rapa* cultivar (*B. campestris L. ssp. chinensis var. purpuria* Hort.; A_hA_h, diploid) were selected as the paternal parent. The F_1 allotriploid hybrids were generated by crossing between two different *B. napus* and the *B. rapa* species. All plant materials were grown under the same field conditions located at the Huazhong Agricultural University (30°28′N, 114°21′W). For sampling, the plant materials were collected at 10.00 – 11.00 am in December (average 8°C). Stem epidermal tissue below the top of the fifth true leaf was collected and immediately frozen in liquid nitrogen. These samples were then used for subsequent ATAC-seq, WGBS, sRNA-seq, and RNA-seq library construction and for determining ions and metabolites.

ATAC-seq analysis

The low-quality reads and adapters from the raw ATAC-seq data were filtered and removed using Trimmomatic (RRID:SCR_011848) [44]. The clean data were aligned to the *B. napus* (Zhongshuang 11, *ZS11*) reference genome by Bowtie2_v2.5.2 (RRID:SCR_016368) [45,46]. The mapped reads in sam format were converted to bam format using SAMtools_v1.9 (RRID:SCR_005227) [47]. Subsequently, MACS2_v2.2 (RRID:SCR_013291) peak calling software was used to identify ATAC-seq peaks [48]. The overlapping peaks over 50 bp in the biological replicates were considered ACRs. The genomic distribution of ACRs and associated genes was confirmed using the ChIPseeker tool (RRID:SCR_021322) [49]. Differential binding events were identified using the DiffBind_v6.0 package [50]. The motifs of the ACRs were identified using HOMER (RRID:SCR_010881) [51]. The term "silent ACR" refers to ACRs identified in parents (reads ≥ 5) but not in F_1 hybrids (reads = 0). On the other hand, the term "novel ACR" refers to ACRs identified in F_1 hybrids (reads ≥ 5) but not in parents (reads = 0).

WGBS analysis

We then used BatMeth2-align with default parameters to map the filtered WGBS reads to the *B. napus* (ZS11) genome [45]. The sequences covering five or more cytosine sites were set as valid methylation sites. Finally, BatMeth2-Meth2BigWig was used to generate BigWig files to identify and visualize differentially methylated regions (DMRs) in IGV [52]. Only cytosine regions with adjusted *p*-values < 0.05 and DNA methylation differences more significant than 0.3, 0.2, and 0.1 (for CG, CHG, and

CHH, respectively) were considered DMRs.

RNA-seq analysis

Trimmomatic was used to remove barcode adaptors, and low-quality reads [44]. The filtered reads were aligned to the *B. napus* (ZS11) reference genome using HISAT2_v2.2.0 with default parameters [53]. The uniquely mapped reads were filtered using SAMtools_v1.9 [47]. Counting and normalizing transcripts per million mapped reads (FPKM) were performed on BAM files using StringTie_v2.1.4 [54]. Genes with FPKM > 1 were defined as expressed genes. Genes with an adjusted p-value < 0.05 found by DESeq2 and a $|\log_2\text{fold change}| \geq 2$ were assigned as differentially expressed [55].

sRNA-seq analysis

The raw sequencing reads were trimmed using cutadapt (RRID:SCR_011841) v3.1 to remove adapters. Subsequently, sRNAs between 18 and 30 nt in length were selected and mapped to the *B. napus* (ZS11) genome and defined into sRNA clusters using Shortstack_v3.8.4 (RRID:SCR_010834) [56]. sRNA-mapped reads were normalized to the total cleaned reads for further analysis.

Elemental mass spectrometry analysis

The concentrations of mineral elements in the stem and epidermis were measured using an inductively coupled plasma-mass spectrometer (ICP-MS) (Perkin Elmer, NexION 300D, Shropshire, UK). The replicate samples for each line were pooled and then subjected to oven-drying at 80 °C for a minimum of 72 h, followed by grinding. Approximately 0.2 g of the dried ground powder was placed in a PTFE digestion tube with 6 mL of concentrated nitric acid, and the tube was tightly sealed and processed in a closed vessel acid digestion microwave (MARSXpress; CEM Corporation, Matthews, NC, USA). After digestion, each digested sample was diluted to 10 mL with deionized water and elemental analysis was performed using an ICP-MS in the standard mode, monitoring 15 elements.

Measurement of metabolites

The measurement of soluble sugars, soluble proteins, total phenolics, total flavonoids, total anthocyanins, and proanthocyanidins in the stem epidermal tissue was performed as described in previous studies [57]. All samples were quantified in triplicate in three independent biological replicates.

Electrophoretic mobility shift assay

PCR amplified the full-length CDS of *BnaA03.bZIP11* and *BnaC03.bZIP11* and then cloned into the pGEX4T-2 (GST) to express the BnaA03.bZIP11 and BnaC03.bZIP11 protein in the *Escherichia coli* DE3 strain in the presence of 0.5 mM IPTG at 28 °C for 12 h. The recombinant BnaA03.bZIP11 and BnaC03.bZIP11 protein was purified using GST 4FF (Pre-Packed Gravity Column) (Sangon Biotech, C600911, Shanghai, China). For the electrophoretic mobility shift assay (EMSA), the Cy5-labeled probes and recombinant proteins were mixed in an EMSA/Gel-Shift binding buffer (Beyotime, GS005, China) at 25 °C for 20 min in the presence or absence of unlabeled competitor DNA. The reaction mixture was then electrophoresed on 6% non-denaturing polyacrylamide gels under ice-water conditions.

Dual-luciferase assay

The full length of *Bna.bZIP11* was amplified by PCR and inserted into the *pGreenII-62SK* for transient overexpression. The 2-kb promoter sequence of *BnaA06.UF3GT* was also amplified by PCR and inserted into the *pGreenII 0800-LUC* vector using a ClonExpress II One Step Cloning Kit (Vazyme, C112, China). According to previously described methods, all relevant effector and reporter constructs were transformed into Arabidopsis mesophyll protoplasts [58]. The dual luciferase assay was then conducted according to the instructions from the Dual-Luciferase® Reporter Assay System (Promega, E1910, WI, USA). The data was expressed as the ratio of firefly to ranila luciferase activity (Fluc/Rluc). Each data point was based on at least three replicates, and three independent experiments were performed for each experiment.

Functional enrichment analysis

Gene function descriptions were obtained from the *B. napus* (*ZS11*) reference genome. The GO enrichment analysis was performed using agriGO2, and terms with an FDR < 0.05 were considered significant.

Identification of additive genes

To identify additive and nonadditive gene expression, we constructed independently *in silico* 'hybrids' by combining the RNA-seq data from sequenced parental individuals in a 1:1 ratio for the *B. napus*

(*s70* and *yu25*) and *B. rapa* (Hort) datasets. This ratio reflects the respective 1:2 genomic contribution of the parents to the F_1 hybrids. The differentially expressed genes (DEGs) were identified by comparing the expression levels of genes between the hybrids and *in silico* hybrids, using the criteria of an adjusted p-value < 0.05 and a $|\log_2 \text{fold change}| \geq 1.5$. These genes are indicated as additive if shown to be no differential, and vice versa for non-additive genes.

Identification of dosage-dependent and independent genes

The identification of dosage-dependent and independent genes was followed in the previous study [59]. Briefly, Pearson correlation tests, employing the Benjamini and Hochberg false discovery rate (FDR) method, were conducted to assess the relationship between gene expression and genotype dosage for the A-subgenome (1/2:2/3:1) and C-subgenome (1/2:1/3:0) genes. The Pearson correlation and multiple test corrections were performed using the adjustment method in R. Genes exhibiting a significant correlation between expression and dosage ($R^2 > 0.64$, FDR < 0.05) were classified as dosage-dependent. In contrast, those with no significant correlation were deemed dosage-independent. For instance, the Pearson correlation coefficient for genomic dosage (1/2:2/3:1) and gene expression values (1.72, 1.96, 2.54) of the gene *BnaA01G0002000ZS* is 0.99, with an R^2 of 0.99, indicating that this gene is dosage-dependent.

RT-qPCR assay

A quantitative reverse transcription PCR (RT-qPCR) assay was performed according to a previous report [41]. The melting curve of RT-qPCR was analyzed to ensure the presence of only one peak. The expression level of each gene was calculated using the $2^{-\Delta\Delta CT}$ method. All analyses were performed at least three times. The *BnaActin7* gene (XM_013858992) was used as an internal control. All primers were listed in Table S1.

Results

Resynthesized allotriploid *B. napus* - *B. rapa* hybrids and phenotypic characterization.

We previously generated two allotriploid hybrids of *Brassica* species by crossing two *B. napus* inbred lines (s*70*, $A_sA_sC_sC_s$; *yu25*, $A_yA_yC_yC_y$) with a *B. rapa* (Hort, A_hA_h) (see Materials and Methods). The

two *B. napus* inbred lines had green stems, while the *B. rapa* had red stems (Fig. 1a). The resulting allotriploid hybrids, designated Hybrid-sh (*s70* × Hort, $A_sA_hC_s$) and Hybrid-yh (*yu25* × Hort, $A_yA_hC_y$), had 29 chromosomes and exhibited red color on stem, similar to the *B. rapa* (Fig. 1a). Interestingly, Hybrid-sh had architectural features similar to *s70*, while Hybrid-yh showed characteristics between the two parentals (Fig. 1a). Additionally, the soluble protein and sugar contents in the two allotriploid F_1 hybrids were approximately 141-168 mg/g and 51-56 mg/g higher, respectively, compared to their respective parental lines (Fig. S1a). The total phenolic content in the allotriploid hybrids was similar to that of the maternal lines and exceeded that of the paternal line (Student's *t*-test, $p < 0.05$; Fig. S1a). The total flavonoid content in the allotriploid F_1 hybrids was approximately 7.2-8.1 mg/g, which was higher than that of the maternal line but lower than that of the paternal line (Fig. S1b). Notably, the flavonoid content of Hybrid-yh was higher than Hybrid-sh's (Student's *t*-test, $p < 0.05$, Fig. S1b).

Genome-wide identification of ACRs in F_1 hybrids and their respective parentals.

We conducted RNA-seq and ATAC-seq of stem epidermis from two allotriploid hybrids (Hybrid-sh and Hybrid-yh) and their corresponding parental lines to investigate the genome-wide transcriptional dynamics during allotriploid hybridization. The RNA-seq generated an average of 20 million clean reads, with 93.4% successfully mapped to the reference genomes (Table S2). Meanwhile, ATAC-seq produced an average of 47 million clean reads per replicate (Table S3). Spearman's rank correlation and principal component analysis (PCA) revealed high-quality RNA-seq and ATAC-seq datasets (Fig. S2a, S2b). The average sequencing depth of accessibility chromatin regions for *s70*, *yu25*, Hybrid-sh, Hybrid-yh, and Hort were quantified, resulting in 17, 18, 20, 19, and 17X values, respectively (Fig. S3). The genome browser results showed that consistent ACR peaks were identified in three biological replicates of the parental line and F_1 hybrids (Fig. S3). The ATAC-seq peaks were enriched in the following samples: 192,786 in *s70*, 155,900 in *yu25*, 137,384 in Hybrid-sh, 135,728 in Hybrid-yh, and 82,149 in Hort (Fig. 1b). The ACR peaks in Hort were less than maternal lines and F_1 hybrids, which may be due to the Hort lacks the C subgenome. We then compared the ACR peaks in different subgenomes in F_1 hybrids and parental lines. The result showed that 71,312, 56,146, 70,413, 68,466, and 82,149 ACR peaks were identified in the A subgenome of *s70*, *yu25*, Hybrid-sh, Hybrid-yh, and Hort, respectively (Fig. 1b). However, in the C subgenome, 121,474, 99,754, 66,971, and 67,262 ACR peaks were identified in *s70*, *yu25*, Hybrid-sh, and Hybrid-yh (Fig. 1b), indicating that the ACR

densities in the C-subgenome are suppressed by hybridization. The ACRs were significantly enriched at gene transcription start sites (TSS) (Fig. 1c). The majority of ACRs were between 200 and 500 base pairs (bp) in length, with the highest enrichment observed at 250 bp (Fig. S4a). Overall, the ATAC-seq data provide a comprehensive and reliable overview of ACRs in allotriploid hybrids and their parental plants.

The distribution of ACRs on the genome was categorized based on their proximity to genes, which were classified as genic (overlapping with a gene), proximal (within 2 kb of a gene), and distal (more than 2 kb away from any gene). Our analysis revealed a positive correlation between gene expression levels and the presence of ACRs in the TSS region (R=0.32, Kruskal-Wallis test, $p < 2.2e\text{-}16$) (Fig. 1d). Conversely, gene expression showed a negative correlation with the distance of TSS region ACRs (R=-0.29, Kruskal-Wallis test, $p < 2.2e\text{-}16$) (Fig. 1d). On average, genes associated with proximal (including TSS and TTS) ACRs were more highly expressed than the genes related to distal and genic ACRs (Fig. 1e). In contrast to distal and genic ACRs, proximal ACR played a predominant role in regulating gene expression, suggesting that the positioning of ACRs is closely related to their transcriptional regulation, which is consistent with previous studies [16,27,60].

ACRs show different convergent distributions after interspecific hybridization.

Interspecific hybridization may lead to the activation of ACR in unopened regions [27,61]. After examining the variation in the distribution of ACRs within genes and across the genomes of parents and hybrids, we found that more than 40% of the ACRs in the F_1 hybrids were located in distal regions (Fig. 2a). In comparison, only 28% were distributed distally in the paternal line (Fig. 2a). After hybridization, there was a 2.8% decrease in Proximal ACRs, with 39.7% in F_1 hybrids compared to 42.5% in the parents (Fig. S4b). Conversely, Distal ACRs showed a 6.7% increase in F_1 hybrids compared to parental ACRs (Fig. S4c), confirming a distinct ACR distribution between parents and F_1 hybrids. These results suggest that the hybridization process induces changes in the distribution and abundance of accessible chromatin regions, particularly in gene regions and their proximity.

The parental lines *s70*, *yu25*, and Hort exhibited 23,770, 18,715, and 27,382 ACRs in the A subgenomes (Fig. 2b). In contrast, 40,755 and 33,220 ACRs were identified in the C subgenomes of *s70* and *yu25*, respectively (Fig. 2b), showing the divergent distribution of ACRs between the A

subgenome and the C subgenome. 23,417 and 22,822 ACRs were identified in the A subgenomes of the Hybrid-sh and Hybrid-yh (Fig. 2b). In comparison, 22,372 and 22,281 ACRs were detected in the C subgenomes of Hybrid-sh and Hybrid-yh, respectively (Fig. 2b). We found no significant difference in the number of ACRs in the A subgenome in F_1 hybrids and their parents (Fig. 2b), indicating a convergent distribution of ACRs in the A subgenome in interspecific hybridization. The number of ACRs in the C subgenome in F_1 hybrids was about half of that in the maternal lines (Fig. 2b), demonstrating that the number of ACRs was positively correlated with the subgenomic dosage. We further analyzed whether there is immediate convergence in the distribution of different types of ACRs (genic, proximal, and distal ACRs) across subgenomes in interspecific hybrids. There was no difference in genic ACR between the F_1 hybrid and its parental A subgenome; however, significant differences were observed between proximal and distal ACRs (Fig. 2b). This suggests that genic ACRs in the A subgenome converge immediately following interspecific hybridization. Notably, in the A subgenome, proximal ACRs were most abundant in the maternal line, followed by F_1 hybrids, while the paternal line exhibited the least abundance (Fig. 2b); in contrast, distal ACRs show the opposite trend (paternal > F_1 hybrids > maternal) (Fig. 2b). However, no significant differences were found in the proportions of the genic, proximal, and distal ACR in the C subgenome (Fig. 2b), suggesting that the different types of ACRs converge immediately upon the halving of the C subgenome following interspecific hybridization.

Identification of novel ACRs in F_1 hybrids.

We identified 969 novel ACRs and 25 silent ACRs in F_1 hybrids (Fig. S5a), following the criteria ACRs with reads > 5 in F_1 hybrids and read = 0 in their parental lines were defined as novel ACRs; the ACRs with reads = 0 in F_1 hybrids and reads > 5 in their parental lines were defined as silenced ACRs [27]. Among them, novel ACRs in the A and C subgenomes were 209 and 760, respectively (Fig. S5b), indicating that the formation of novel ACRs in the C subgenome was more than in the A subgenome. The distribution of novel ACRs revealed that the proportions of ACRs in the genic, proximal, and distal were 35.7%, 32.4%, and 32.9%, respectively (Fig. 2c). Additionally, the distribution of novel ACRs was primarily observed at the telomeres, situated distally to the centromere (Fig. S5c). Of these novel ACRs, 375 (38.7%) were classified as TE-driven ACRs, with LTR/Gypsy-type retrotransposons playing a significant role, accounting for 30.4% compared to other types of transposable elements (Fig.

2d). We found that gene expression in the proximal regions of non-TE-mediated ACRs was significantly higher than that of TE-mediated ACRs (Fig. 2e). Transposable element (TE)-mediated ACRs may exhibit lower gene expression levels due to the influence of methylation on both the TE and its adjacent sequences [12,63]. In contrast, non-TE-mediated ACRs are typically unaffected by transposable elements; consequently, DNA methylation levels in these regions may be reduced, thereby facilitating the expression of genes in neighboring areas.

Differentially expressed accessible chromatin regions (DE-ACRs) were then compared between the two hybrids. 1,478 DE-ACRs were identified in Hybrid-yh compared to Hybrid-sh (Fig. 2f). The number of differentially up-regulated ACRs and differentially down-regulated ACRs were 766 and 712, respectively (Fig. 2f). Interestingly, in the F_1 hybrids, the number of DE-ACRs in C-subgenome (855) was significantly higher than that in A-subgenome (593) (Fig. 2f), demonstrating a positive correlation between the number of DE-ACRs and genome size. The proximal DE-ACRs were more than genic and distal DE-ACRs (Fig. 2g). These results suggest that the distribution of DE-ACRs was uneven across the genome in F_1 hybrids.

Non-additive gene expression in F_1 hybrids.

Previous studies have emphasized the potential role of non-additive genes in resynthesized plant materials [41,64]. Gene expression quantification was then performed to examine allotriploid hybrids' and their parents' pre-existing gene expression levels. This involved comparing *in silico* hybrids (where the RNA-seq data combined maternal and paternal in a 1:1 ratio) with the F_1 hybrids. The results showed that most expressed genes exhibited additive expression patterns in Hybrid-sh (82.9%) and Hybrid-yh (90.1%) (Fig. S6a). More than 86% of the additive genes were conserved in the two hybrids (Fig. S6b). GO enrichment analysis of conservative additive genes revealed enrichment for cellular, rhythmic, metabolic, and carbon-nitrogen utilization (Table S4). This suggests that conserved additive genes are necessary for normal plant growth and development. However, a total of 7,565 and 4,217 genes showed non-additive expression in Hybrid-sh and Hybrid-yh, respectively (Fig. 3a). In Hybrid-sh, there were 2,508 non-additively up-regulated genes and 5,058 non-additively down-regulated genes (Fig. 3a). In contrast, Hybrid-yh exhibited 482 non-additively up-regulated genes and 3,646 non-additively down-regulated genes (Fig. 3a). The non-additively up-regulated genes in Hybrid-sh were mainly associated with responses to biotic and abiotic stimuli, immune responses, and metabolic

processes (Table S5). Conversely, non-additively up-regulated genes in Hybrid-yh were enriched in ion transport, response to hormones, and metabolic processes (Table S6). This suggests that GO enrichment of non-additively up-regulated genes in F_1 hybrids suggests an enhanced capacity for metabolite production and improved tolerance to environmental conditions. Furthermore, 2,530 identical non-additive expressed genes (2,313 down-regulated and 217 up-regulated) were isolated in both Hybrid-sh and Hybrid-yh (Fig. 3b). These down-regulated genes were associated with primary meristem tissue development, organic acid transmembrane transport, phloem transport, and auxin polar transport by GO enrichment analysis (Table S7). In contrast, the up-regulated genes were enriched with response to external biotic stimulus, lipid metabolic process, and defense response pathways (Table S8).

There are two main categories within non-additive genes: expression level dominance (ELD) and transgressive [64]. Based on gene expression level, the transgressive genes could be further categorized as transgressive up-regulated genes and transgressive down-regulated genes [64]. Approximately 3,163 and 645 transgressive expression genes were identified in Hybrid-sh and Hybrid-yh, respectively (Fig. 3c). In Hybrid-yh, the number of down-regulated genes (464) was significantly higher than the number of up-regulated genes (181) (Fig. 3c). In comparison, no difference was observed between the number of up-regulated (1,503) and down-regulated (1,660) transgressive genes in Hybrid-sh (Fig. 3c). These results suggest that the expression levels of genes in Hybrid-yh show relative stability in the hybrid offspring, with a greater likelihood of inheriting the expression pattern from the parent.

Previous studies have demonstrated a significant positive correlation between ACRs and gene expression [17,27]. In F_1 hybrids, chromatin accessibility was found to be higher than in the parents for transgressively up-regulated genes (Hybrids > Maternal = Paternal) (Fig. 3d). Conversely, for transgressive down-regulated genes, the chromatin accessibility was significantly lower in F_1 hybrids than in the parents (Paternal > Maternal > Hybrids) (Fig. 3d). Notably, more than half of the novel ACRs were found to target transgressive up-regulated genes (Fig. 3e). The transgressive up-regulated genes in Hybrid-sh showed significant GO enrichment in metal ion binding, especially magnesium ion, calcium ion, and potassium ion binding, as well as secondary metabolic processes (Fig. S7a). In particular, two genes, *POT4* (*POTASSIUM TRANSPORTER 4*) and *POT2* (*POTASSIUM TRANSPORTER 2*), which are involved in the regulation of potassium ion uptake and transport in

plants [65,66], were identified. Two novel ACRs associated with *BnaC04.POT2* and *BnaA01.POT4* were identified in their promoters (Fig. 3f, Fig. S7b). The expression of *BnaC04.POT2* and *BnaA01.POT4* was significantly higher in Hybrid-sh than the parental lines (Fig. 3g, Fig. S7c). As expected, Hybrid-sh has a significantly higher potassium content than the parental lines (Fig. 3h). Previous studies have shown that the loss of the *bZIP48* gene function in rice results in increased sensitivity to zinc deficiency, while the *bZIP44* gene is involved in the plant's response to cadmium tolerance [58,67]. Notably, the bZIP transcription factor binding *cis*-element, ACGT, was identified in the novel ACR of *BnaA01.POT4* promoter region (Fig. 3f). Based on these findings, we hypothesize that *bZIP48* and *bZIP44* may interact with *BnaA01.POT4*, thereby playing a role in potassium ion transport.

To investigate the differences in mineral element content between two F_1 hybrids and their parents, we analyzed 16 mineral elements, including five major elements (e.g., Mg) and eight trace elements (e.g., B) (Table S9). The results showed that the major elements (e.g., Ca) were significantly higher in F_1 hybrids than their parents. However, the trace elements (e.g., B) showed additive effects, and other elements such as Fe, Zn, Cu, and Mo showed no significant differences between F_1 hybrids and their parents. These results suggest that genetic factors regulate the mineral element content in F_1 hybrids and are not solely determined by the additive effects of the parent plants.

Bna.bZIP11 in SPA-ACR regulates the expression of _BnaA06.UF3GT_ to promote the accumulation of anthocyanins.

The *c*-means clustering method was then used to classify the differential peaks according to chromatin accessibility levels in F_1 hybrids and their respective parents, resulting in nine clusters labeled C1 to C9 (Fig. 4a, Fig. S8a). For ACRs, the ATAC-seq peaks were detected in only one parent, while they were not detected in the other parent, termed single-parent activation ACRs (SPA-ACRs). These SPA-ACRs could be further categorized into two patterns: SPA-M ACRs, where the ACRs were detected in the maternal and F_1 hybrids but not in the paternal, and SPA-P ACRs, where the ACRs were detected in the paternal and F_1 hybrids but not in the maternal. Since the Hort lacks the C-subgenome, it's reasonable to expect a limited number of ATAC-seq peaks in the Hort materials compared to those in the maternal (AACC) and F_1 hybrid (AAC) genomes. We then focused the analysis only on peaks corresponding to the A-subgenomes. A total of 8,125, 9,681, and 22,885 SPA-ACRs were identified in

the A_s (A-subgenome of *s70*, *B. napus*), A_y (A-subgenome of *yu25*, *B. napus*), and A_h (A genome of *B. rapa*) subgenomes (Fig. 4a, Fig. S8a), which were represented in cluster C5 and cluster C8, respectively (Fig. 4a, Fig. S8a). The SPA-ACR can lead to the emergence of single parental expression (SPE), a gene expressed in only one parent but is silent in the other parent after interspecific hybridization [68]. On average, 336 genes expressed in the F_1 hybrids were exclusively expressed in the maternal parents (SPE-M, A_s and A_y), and 277 genes expressed in the F_1 hybrids were exclusively expressed in the paternal parent (SPE-P) in the A genome (A_h) (Fig. S8b). Approximately 20% of the SPE genes were correlated with SPA-ACR (Fig. S8c).

The datasets were then prioritized based on the occurrence of Proximal SPA-P ACRs and SPE-P genes. Notably, the top 10 ranked in the SPA-P genes showed a significant presence of genes associated with anthocyanin synthesis (Fig. S8d). For example, we found that the chromatin accessibility of *BnaA06.UF3GT*, which encodes the flavonoid 3-O-glucosyltransferase (UF3GT) enzyme [69], was more significant in the hybrids and the paternal promoter region compared to the maternal promoter region (Fig. 4b). The expression of the *BnaA06.UF3GT* was significant in F_1 hybrids and paternal individuals, while no expression was detected in the maternal plant (Fig. 4c). Analysis using the HOMER algorithm showed that the *BnaA06.UF3GT* gene might be regulated by the *BnabZIP11* transcription factor (Fig. 4b). To investigate this, a luciferase (LUC) gene driven by a ~2 kb promoter of *BnaA06.UF3GT* was used as a reporter, and three *BnabZIP11* genes were driven by the *CaMV 35S* promoter (Fig. 4d). Co-transformation of the vector containing *BnaA03.bZIP11*, *BnaC03.bZIP11*, and *BnaC01.bZIP11* with the *pBnaA06.UF3TG-LUC* construct significantly increased LUC/REN activities by 4.95, 3.81, and 3.76-fold, respectively, compared to the expression of *pBnaA06.UF3TG-LUC* alone (Fig. 4d). Further analysis revealed the binding of the BnabZIP11 transcription factor to the G-box elements upstream of *BnaA06.UF3GT* (Fig. 4e), suggesting that *BnabZIP11* may directly bind to the G-box elements upstream of the *BnaA06.UF3GT* promoter and thereby positively regulate the expression of *BnaA06.UF3TG*. The expression patterns of genes involved in anthocyanin synthesis and related transcription factors were also highly expressed in the F_1 hybrids and the paternal parent (Fig. 4f, Fig. S9). This finding suggests a possible reason for the high accumulation of total anthocyanins and phenolics in the paternal and hybrid plants (Fig. S1b). The activation of ACRs by a single parent after interspecific hybrid genome recombination is positively associated with gene

regulation, which may contribute to the non-additive phenotypic traits observed in resynthesized F_1 hybrids. The interaction between the activated ACRs and gene expression patterns may lead to altered gene regulatory networks, resulting in unique phenotypic traits in hybrids that differ from those of either parent.

Hybridization-induced DNA methylation related to parents in F_1 hybrids.

The process of hybridization often results in significant reprogramming of global DNA methylation. This can be considered an "epigenetic shock" resulting from the fusion of different epigenomes from the parental plants [22,70,71]. Therefore, we evaluated the total methylation levels in hybrids and compared them with the parental methylation levels. The sequencing depth of WGBS was approximately 30-fold genome coverage, and the bisulfite conversion was greater than 99% in all samples (Table S10). The sRNA-seq data generated an average of 20 million clean reads per replicate, of which 93.4% were mapped to the reference genome (Table S11). PCA plots and Spearman's rank correlation coefficients generated from the sRNA-seq and WGBS libraries showed clear clustering patterns and reproducibility across biological replicates (Fig. S10a, Fig. S10b). At the chromosomal level, a bias in the total methylation levels of CG, CHG, and CHH towards the hypermethylated parent was observed (Fig. S11a). Further, the methylation levels of TE bodies in F_1 hybrids and their parental lines across the whole genome were analyzed. We identified 25.9-30.6%, 35.0-40.2%, and 39.7-44.3% methylated TEs in the CG, CHG, and CHH contexts of F_1 hybrids, respectively (Fig. S11b). These results were not significantly different from those of the maternal line (*s70* and *yu25*) (Fig. S11b). In comparison to the paternal line (Hort), the observed proportions of TEs in F_1 hybrids were 13.5-13.9% in the CG context, 19.6-20.0% in the CHG context, and 22.6-22.7% in the CHH context (Fig. S11b). This indicates that the F_1 hybrids tended to inherit higher methylation levels from the parent with higher methylation.

To investigate the changes in DNA methylation in F_1 hybrids after interspecific hybridization, two *in silico* hybrids (*in silico*-sh and *in silico*-yh) were first constructed by combining maternal and paternal WGBS data in a 1:1 ratio. All DNA methylation levels (CG, CHG, and CHH) of Hybrid-sh were higher than those of *in silico*-sh, while no significant difference in the methylation levels of CG and CHG was found between Hybrid-yh and *in silico*-yh (Fig. 5a). However, the methylation level of CHH was lower in Hybrid-yh than *in silico*-yh (Fig. 5a). Compared to *in silico* hybrids, 24,693 and

9,214 DMRs (Differentially Methylated Regions) were identified in Hybrid-sh and Hybrid-yh, respectively (Fig. 5b). There were fewer hypo-DMRs (9,701) compared to hyper-DMRs (13,492) in Hybrid-sh (Fig. S12a). 9,214 DMRs were found in Hybrid-yh, and nearly half of the DMRs (49%) were CHH-DMRs (Fig. S12a). Compared to Proximal and Distal DMRs, a more significant increase in Genic DMRs was identified for CG methylation in both Hybrid-sh and Hybrid-yh (Fig. 5b). For CHG methylation, the distribution of DMRs was as follows: distal > proximal > genic (Fig. 5b). The distribution of DMRs at gene locations suggests that the context of DNA methylation influences this distribution. More than 68% of the differentially methylated genes (DMGs) were Proximal DMGs (Fig. 5c). Transcript levels of proximal DMGs were then compared between F_1 hybrids and *in silico* hybrids. No significant difference was observed in the expression of DMGs associated with CHG (Fig. 5d). However, the expression of hyper DMGs of CHH was significantly higher in F_1 hybrids than *in silico* hybrids (Fig. 5d). In contrast, hypo DMGs showed the opposite trend (Fig. 5d).

In addition, we identified 9,464 differentially methylated regions (DMRs) in Hybrid-sh and Hybrid-yh, and 2,370, 1,289, 1,518 hyper-DMRs, 2,317, 1,256, and 714 hypo-DMRs in Hybrid-sh CG, CHG, and CHH contexts compared to Hybrid-sh (Fig. S12b). Furthermore, in the context of differential methylation loci (DMLs), we also observed 5,323, 2,271, and 5,902 hyper-DMLs, as well as 5,448, 1,840, and 3,503 hypo-DMLs in the CG, CHG, and CHH contexts, respectively, when compared to Hybrid-sh (Fig. S12b). The difference in the number of hyper-DMRs (hyper-DMLs) and hypo-DMRs (hypo-DMLs) in the two hybrids was caused by the DMRs (DMLs) in the CHH context, which highlighted unbalanced Hyper- and Hypo-CHH methylation changes between the two hybrids.

TE involvement was detected in approximately 55.6% of DMRs; further analysis revealed that 21.5% and 39.1% of these DMRs were LINE and LTR (Fig. S13a). To gain additional insight into the relationship between sRNA-mediated DNA methylation and hybridization-induced hyper- and hypo-DMRs, we analyzed changes in sRNA enrichment within hyper- and hypo-DMRs. In hyper-DMRs, we observed a significant increase in sRNA accumulation *in silico* compared to F_1 hybrids, whereas the opposite trend was seen in hypo-DMRs (Fig. S13b). This highlights the close relationship between DMRs and sRNA accumulation.

DNA methylation in ACR associated with TEs in F_1 hybrids.

Previous reports indicate that DNA methylation is critical in maintaining and losing chromatin accessibility [26,28,29]. When comparing the DNA methylation levels of TEs, gene bodies, and ACRs, we found that ACRs had significantly higher methylation levels compared to gene bodies but lower than TEs (Kruskal-Wallis test, $p < 2.2e\text{-}16$, Fig. 5e). Further analysis revealed that 25.6% of ACRs and 4.0% of genes were derived from TEs (Fig. 5f), indicating a substantial overlap between ACRs and TEs. This finding suggests that the increased DNA methylation levels observed in ACRs may be associated with TEs, potentially explaining the observed differences between ACRs and gene bodies.

The level of DNA methylation in ACRs varies depending on their position in the genome. ACRs with a CG context show different levels of DNA methylation, with a consistent decrease in methylation levels between TEs, Distal, Genic, and Proximal regions (TE > Distal > Genic > Proximal; Kruskal−Wallis, $p < 2.2e{-}16$; Fig. 5g). However, in the context of CHG and CHH, there was no significant difference between the methylation levels of the Genic and Proximal regions (Fig. 5g). Overall, TE and Distal ACR showed significantly higher methylation levels compared to Genic and Proximal ACRs (Fig. 5g). This suggests that TE and distal regions may require higher levels of DNA methylation for stability. In contrast, genic and proximal regions may be more sensitive to gene expression regulation, resulting in lower DNA methylation levels. Gene expression analysis also supported this observation, as the expression of genes associated with ACRs lacking DNA methylation was significantly higher compared to genes associated with methylated ACRs (Fig. 5h). For example, in Hybrid-sh, an open chromatin region was observed in the promoter region of *BnaC02.PRL1*, which did not inherit the methylation sites from the parent; conversely, in Hybrid-yh, the situation was reversed (Fig. 5i). RNA-seq and RT-qPCR results showed that *BnaC02.PRL1* was not detected in Hybrid-yh, but was significantly expressed in Hybrid-sh (Student's *t*-test, $p < 0.05$, Fig. S13c). This indicates that DNA methylation plays a role in repressing gene expression within ACRs, and unmethylated ACRs may be more transcriptionally active.

Genome dosage affects accessible chromatin regions in F_1 hybrids.

Genome-wide dose-dependent and independent regulation contribute to the evolution and gene expression of plant polyploids [59,72], and genome imbalance in AAC hybrids may lead to changes in gene expression and variation in epigenetics. We then calculated the correlation coefficient (*R*-values) between the expression of 96,992 genes and the relative doses of the genes in the A and C subgenomes.

19,501-25,011 and 22,554-26,420 dose-dependent genes were identified in the A and C-subgenomes, respectively (Fig. 6a). Interestingly, approximately 56% and 16% of the dose-dependent and dose-independent genes, respectively, were consistent in the two F_1 hybrids (Fig. S14a), indicating that dose-dependent genes are more conserved in F_1 hybrids.

Pearson's correlation test was used to divide all homologous genes into two groups based on the coefficient of determination (R^2) to compare the characteristics of dose-dependent and dose-independent homologous genes. Homologous genes with statistically significant correlations were designated dose-dependent A (Ad) and C (Cd) genes. In contrast, those with insignificant correlations were designated dose-independent A (Ai) and C (Ci) genes. The proportion of dose-dependent genes among the homologous genes in the A/C-subgenome was higher than that of genome-unique genes (Fig. 6b). Among the four categories of homologous genes (AdCd, AdCi, AiCd, and AiCi), AdCd had the highest number (20,888), followed by AiCd (7,746). At the same time, AdCi and AiCi had the lowest number (4,927 and 5,552, respectively) (Fig. 6c, Fig. 6d). Chromatin stacking was observed to be highest in the ACR for AdCd, followed by AdCi, and lowest for AiCi (Fig. 6e), indicating that dose-dependent homologous genes have increased chromatin accessibility, making them more susceptible to regulation by transcriptional and regulatory factors. Higher levels of DNA methylation were found in the CG context for AdCd and AdCi compared to AiCd and AiCi, while in the CHG and CHH contexts, AdCd had the lowest methylation levels among the four categories and AiCi had the highest (Fig. 6f). This suggests that different DNA methylation modifications may regulate different gene categories. In both AACC and AAC, the methylation level of the C-subgenome was higher than that of the A-subgenome, regardless of whether the dose of the C-subgenome (Fig. 6g). This suggests that factors beyond genomic dosage regulate DNA methylation levels. In AACC, the ACR level of the C-subgenome was higher than that of the A-subgenome; however, in AAC, the ACR level of the A-subgenome was significantly higher than that of the C-subgenome (Fig. 6h). This demonstrates that in AAC, doubling the dosage of the A-subgenome results in a significant increase in the ACR level of the A-subgenome, highlighting the strong effect of genome dosage on accessible chromatin regions. Dose-dependent and dose-independent genes were identified in the A- and C-subgenomes. The dose-dependent genes exhibited higher chromatin openness. Notably, differences in the A-subgenome were specifically located in the TSS and upstream regions, whereas differences in the C-subgenome were

observed more broadly across the genome (Fig. S14b).

Discussion

Interspecific hybridization is essential in plant breeding and genetic improvement [7,73,74]. In this study, we resynthesized two interspecific F_1 hybrids by crossing different genotypes of *B. napus* and *B. rapa* to elucidate the intricate relationship between accessible chromatin regions, DNA methylation, and the expression of transgressive genes. The interplay between DNA methylation, TEs, and sRNA contributes to the dynamic landscape of ACRs during interspecific hybridization, resulting in distinct gene expression patterns across the genome.

Accessible chromatin regions and DNA methylation differ in genome dosage effects.

In comparative studies focusing on gene expression patterns within polyploid plant species, it has been observed that most genes across different subgenomes exhibit coordinated expression behavior, either in a dosage-dependent or dosage-independent manner [59,72]. Specifically, in the resynthesized *Arabidopsis* allotetraploid, approximately 56% of the alleles showed congruent expression patterns, with a predominant dosage-dependent expression (46% TdAd) complemented by a smaller proportion of dosage-independent expression (10% TiAi) [59]. Similar trends were noted in synthetic *Brassica napus* and its derivatives, where 58% of A and C-subgenome genes align in the same direction, with 40% exhibiting dosage-dependent expression (AdCd) and 18% showing dosage-independent expression (AiCi). Conversely, 42% have divergent expression patterns (22% AdCi and 20% AiCd) [72]. In our study, 66% of genes displayed coherent expression, either dosage-dependent (54% AdCd) or dosage-independent (12% AiCi). However, a significant proportion of 32% still presents contrasting expression dynamics (10% AdCi and 24% AiCd). Notably, there remains a substantial fraction of genes (ranging from 32% to 44%) that manifest discordant expression patterns between subgenomes, reflecting different genetic backgrounds between *Arabidopsis* and *Brassica* species, and also the intricate interplay between gene dosage effects and the regulatory networks that modulate gene expression in polyploid genomes. In polyploids, allele mutations may be buffered by extra gene copies, reducing negative selection and increasing survival chances for individuals with mutations in dosage-dependent genes [59]. These findings underscore the prevalence of coordinated and independent regulatory mechanisms influencing gene expression in polyploid genomes. This necessitates a nuanced

understanding of the genetic and epigenetic factors that govern these patterns.

At the mechanistic level, chromatin accessibility is essential for gene transcription as it allows transcription factors and RNA polymerase to bind to DNA and initiate gene expression [13]. Changes in chromatin accessibility can directly impact gene expression levels, leading to significant dosage effects [27,59,60]. In other plant species (e.g., cotton, sorghum, and *Arabidopsis*), accessible chromatin regions were consistently found to be positively correlated with the expression of nearby genes, and highly expressed genes exhibited distinct peaks around their transcription start sites (TSS) [14,16,27]. In contrast, DNA methylation has a more indirect effect on gene expression. It can influence gene expression by inhibiting transcription factor binding and recruiting inhibitory protein complexes [75,76]. These mechanisms may interact complexly and involve feedback regulation, making the relationship between DNA methylation and gene expression non-linear and resulting in an insignificant dose effect. In the resynthesized *Arabidopsis* allotetraploid, CHG methylation levels were higher in the genic regions of dosage-independent alleles than dosage-dependent ones [59]. Conversely, the 5' regions of dosage-dependent alleles exhibited greater methylation than those of dosage-independent alleles [59]. Furthermore, DNA methylation is dynamic and highly specific to different cell types [77]. Despite alterations in genome dosage, DNA methylation levels can remain relatively stable or change only in particular cell types [78,79]. On the other hand, chromatin accessibility is also dynamic and cell-type specific, but it is more responsive to changes in genome dosage [27,80]. For example, significantly different distributions of DHSs were observed between the A and D subgenomes in F_1 cotton hybrids [27]. The genome size and polyploidy also affect the distal DHS distribution [27]. These indicate that changes in chromatin structure serve as a quick mechanism for regulating gene expression in direct response to alterations in genome dosage.

Interspecific hybridization triggers genome recombination leading to novel accessible chromatin regions.

During plant hybridization, the unequal retention of epigenetic marks, particularly accessibility chromatin regions (ACRs) activated by single parents or novel, can significantly affect gene expression and trait development in hybrid offspring. In *Camellia sinensis*, a large number of accessible chromatin regions is observed after interspecific hybridization [61]. The 'newborn DHS' (nbDHS) after cotton polyploidization suggests that these nbDHS may arise from transposable elements during or after

polyploidization [27]. We found 512 novel ACRs near the transgressive genes in F_1 hybrids, 38.7% of these novel ACRs were classified as TE-driven ACRs, suggesting that interspecific hybridization triggers genome recombination that activates ACR formation. SPA-ACRs suggest that only one parent may control the expression pattern of certain gene regions during genome recombination. The maintenance of this expression pattern may be influenced by asymmetric chromatin openness, which can lead to increased expression or silencing of specific genes in hybrid offspring [61,81]. Notably, most SPA-ACRs are found within the gene bodies that encode the proteins, suggesting a possible relationship between the expression pattern of single-parent activation and structural features of the gene's transcriptional activity region. In addition, the higher expression levels of genes adjacent to SPA-ACRs provide further evidence of a direct link between this expression pattern and increased gene expression. In wheat, expression of the *ph1* gene is associated with chromatin remodeling, which alters chromatin structure so that only genes from a particular parent are expressed [82].

Differences in the proportions of DNase I hypersensitive sites (DHS) in distal and proximal regions have been observed during wheat polyploidization, suggesting that the distribution of DHS varies among different genomes [12]. Our results indicate that interspecific hybridization induces changes in the distribution and abundance of chromatin regions accessible to genic and proximal regions. We found that the number of differentially expressed ACRs (DE-ACRs) in the two hybrids was highest in the proximal, followed by the genic, and lowest in the distal DE-ACRs (Fig. 2g). The distribution of DE-ACRs in the proximal may be related to the regulation of gene expression [14]. Proximal regions usually include the promoters directly regulating gene expression [12]. Therefore, the activity of these regions may change significantly in different hybrids or cell types, leading to the detection of more proximal DE-ACRs. In addition, the DNA methylation level of the distal region was considerably higher than that of the proximal region (Fig. 5g), indicating that the distal region was relatively stable.

Accessible chromatin regions and DNA methylation jointly regulate gene expression.

There is a close relationship between DNA methylation and open chromatin regions [25,26]. DNA methylation typically occurs on CpG islands, where its primary role is suppressing gene expression. Methylated CpG islands often result in a more condensed chromatin structure, limiting the binding of transcription factors and other regulatory proteins and ultimately repressing gene expression [70,83].

In contrast, open chromatin regions have a more flexible chromatin structure, allowing easier binding of transcription factors and regulatory proteins, thus promoting gene expression. Significant positive correlations have been observed between chromatin accessibility and gene expression levels in nearby genes in our study. We further found that the DNA methylation level of the ACR located at the proximal end of the gene is much lower than that of the TE and distal regions (Fig. 5g). This may be because the proximal ACR usually contains promoters and enhancers and is an important region for gene transcription regulation. Low levels of DNA methylation help keep these regions open, making it easier for transcription factors and other regulatory proteins to bind, thereby promoting gene expression [25,29]. Genome-wide chromatin accessibility maps for 18 *Arabidopsis* mutants that lack CG, CHG, or CHH methylation demonstrated that DNA methylation in all three sequence contexts influences chromatin accessibility [26]. Variations in DNA methylation found in lettuce may affect chromatin accessibility, altering the expression levels of associated proximal and distal genes [29]. Similar results were observed in rice domestication [84]. The soybean *CMT* mutant, *Gmcmt*, exhibits significant hypomethylation at non-CG (CHG and CHH) DNA methylation sites, which enhanced chromatin accessibility and significantly regulated the expression of hundreds of functionally relevant genes (e.g., *GOLDEN-LIKE10* (*GmGLK10*)), which further strengthen photosynthesis and unexpectedly boosted rhizobia's fixation efficiency [85]. These results suggest that DNA methylation and ACR's delicate balance are essential for maintaining normal gene expression patterns.

Conclusions

Overall, our findings provide valuable insights into the role of accessible chromatin and DNA methylation in driving gene expression in hybrids (Fig. 7). We observed significant differences in the expression of non-additive genes among the different F_1 hybrids, with up-regulated transgressive genes associated with metal ion accumulation and SPE genes related to anthocyanin accumulation. This study contributes to our preliminary understanding of how accessible chromatin regions and DNA methylation regulate metabolite and ion accumulation in F_1 hybrids.

Ethical statement

This article does not contain any studies with human or animal subjects.

Declaration of competing interest

The authors declare that they have no known competing financial interests or personal relationships that could have appeared to influence the work reported in this paper.

Acknowledgments

We thank the National Key Laboratory of Crop Genetic Improvement of Huazhong Agricultural University for providing the bioinformatics computing platform, Dr. Shengwei Dou from Shandong Agricultural University for the experimental support, and Novogene provides sequencing services. This work was supported by the Science and Technology Innovation 2030-Major Project (2023ZD04068) to Cheng Dai and the National Natural Science Foundation of China (No. 32172070) to Chaozhi Ma.

Competing interests

The authors have no conflicts of interest to declare.

Author contributions

D.C., Q.C., and M.C. conceived the original idea. **Z.Q., C.K.,** and **Z.X.** performed experiments; **Q.C.** and **C.G.** designed the work and analyzed the data; **Q.C.** and **D.C.** wrote and discussed the paper with all authors.

Data availability

All sequence data generated in this study have been deposited in the BIG data under the BioProject accession number PRJCA023096 and NCBI BioProject: PRJNA1154317. All additional supporting data are available in the *GigaScience* repository, GigaDB [86].

References

1. Baack EJ, Rieseberg LH. A genomic view of introgression and hybrid speciation. Curr Opin Genet Dev 2007;17(6): 513-8.
2. Chen ZJ. Genomic and epigenetic insights into the molecular bases of heterosis. Nat Rev Genet 2013;14(7): 471-82.
3. Doebley JF, Gaut BS, Smith BD. The molecular genetics of crop domestication. Cell 2006;127(7): 1309-21.
4. Hochholdinger F, Hoecker N. Towards the molecular basis of heterosis. Trends Plant Sci 2007;12(9): 427-32.
5. Li Z, Zhu A, Song Q, Chen HY, Harmon FG, Chen ZJ. Temporal regulation of the metabolome and proteome in

photosynthetic and photorespiratory pathways contributes to maize heterosis. Plant Cell 2020;32(12): 3706-22.

6. Zhang C, Yang Z, Tang D, Zhu Y, Wang P, Li D, et al. Genome design of hybrid potato. Cell 2021;184(15): 3873-83.

7. Liu W, Zhang Y, He H, He G, Deng XW. From hybrid genomes to heterotic trait output: Challenges and opportunities. Curr Opin Plant Biol 2022;66: 102193.

8. Groszmann M, Greaves IK, Fujimoto R, Peacock WJ, Dennis ES. The role of epigenetics in hybrid vigor. Trends in Genetics 2013;29(12): 684-90.

9. Brown K, Takawira LT, O'Neill MM, Mizrachi E, Myburg AA, Hussey SG. Identification and functional evaluation of accessible chromatin associated with wood formation in Eucalyptus grandis. New Phytol 2019;223(4):1937-1951

10. Maher KA, Bajic M, Kajala K, Reynoso M, Pauluzzi G, West DA, et al. Profiling of Accessible Chromatin Regions across Multiple Plant Species and Cell Types Reveals Common Gene Regulatory Principles and New Control Modules. Plant Cell 2018;30(1):15-36.

11. Potter KC, Wang J, Schaller GE, Kieber JJ. Cytokinin modulates context-dependent chromatin accessibility through the type-B response regulators. Nat Plants 2018;4(12):1102-1111.

12. Jordan KW, He F, de Soto MF, Akhunova A, Akhunov E. Differential chromatin accessibility landscape reveals structural and functional features of the allopolyploid wheat chromosomes. Genome Biol 2020;21(1): 176.

13. Wang FX, Shang GD, Wu LY, Xu ZG, Zhao XY, Wang JW. Chromatin Accessibility Dynamics and a Hierarchical Transcriptional Regulatory Network Structure for Plant Somatic Embryogenesis. Dev Cell 2020;54(6):742-757.

14. Tian H, Li Y, Wang C, Xu X, Zhang Y, Zeb Q, et al. Photoperiod-responsive changes in chromatin accessibility in phloem companion and epidermis cells of Arabidopsis leaves. Plant Cell 2021;33(3): 475-91.

15. Klemm SL, Shipony Z, Greenleaf WJ. Chromatin accessibility and the regulatory epigenome. Nat Rev Genet 2019;20(4):207-220.

16. Zhou C, Yuan Z, Ma X, Yang H, Wang P, Zheng L, et al. Accessible chromatin regions and their functional interrelations with gene transcription and epigenetic modifications in sorghum genome. Plant Commun 2020;2(1):100140.

17. Zhang Y, Chen G, Deng L, Gao B, Yang J, Ding C, et al. Integrated 3D genome, epigenome and transcriptome analyses reveal transcriptional coordination of circadian rhythm in rice. Nucleic Acids Res 2023;51(17):9001-9018.

18. Ricci WA, Lu Z, Ji L, Marand AP, Ethridge CL, Murphy NG, et al. Widespread long-range cis-regulatory elements in the maize genome. Nat Plants 2019;5(12):1237-1249.

19. Lu Z, Marand AP, Ricci WA, Ethridge CL, Zhang X, Schmitz RJ. The prevalence, evolution and chromatin signatures of plant regulatory elements. Nat Plants 2019;5(12):1250-1259.

20. Mathieu O, Reinders J, Čaikovski M, Smathajitt C, Paszkowski J. Transgenerational stability of the Arabidopsis epigenome is coordinated by CG methylation. Cell 2007;130(5): 851-62.

21. Shirai K, Sato MP, Nishi R, Seki M, Suzuki Y, Hanada K. Positive selective sweeps of epigenetic mutations regulating specialized metabolites in plants. Genome Res 2021;31(6): 1060-1068.

22. Zhu W, Hu B, Becker C, Doğan ES, Berendzen KW, Weigel D, et al. Altered chromatin compaction and histone methylation drive non-additive gene expression in an interspecific Arabidopsis hybrid. Genome Biol 2017;18(1):157.

23. Ma X, Xing F, Jia Q, Zhang Q, Hu T, Wu B, et al. Parental variation in CHG methylation is associated with allelic-specific expression in elite hybrid rice. Plant Physiol 2021;186(2):1025-1041.

24. Zhang K, Zhang L, Cui Y, Yang Y, Wu J, Liang J, et al. The lack of negative association between TE load and subgenome dominance in synthesized Brassica allotetraploids. Proc Natl Acad Sci U S A 2023;120(42):e2305208120.

25. Han Y, Lu M, Yue S, Li K, Dong M, Liu L, et al. Comparative methylomics and chromatin accessibility analysis in Osmanthus fragrans uncovers regulation of genic transcription and mechanisms of key floral scent production. Hortic Res 2022;9:uhac096.

26. Zhong Z, Feng S, Duttke SH, Potok ME, Zhang Y, Gallego-Bartolomé J, et al. DNA methylation-linked chromatin accessibility affects genomic architecture in Arabidopsis. Proc Natl Acad Sci U S A 2021;118(5):e2023347118.
27. Han J, Lopez-Arredondo D, Yu G, Wang Y, Wang B, Wall SB, et al. Genome-wide chromatin accessibility analysis unveils open chromatin convergent evolution during polyploidization in cotton. Proc Natl Acad Sci U S A 2022;119(44):e2209743119.
28. Crisp PA, Marand AP, Noshay JM, Zhou P, Lu Z, Schmitz RJ, et al. Stable unmethylated DNA demarcates expressed genes and their cis-regulatory space in plant genomes. Proc Natl Acad Sci U S A 2020;117:23991-24000.
29. Cao S, Sawettalake N, Li P, Fan S, Shen L. DNA methylation variations underlie lettuce domestication and divergence. Genome Biol 2024;25:158.
30. Song K, Lu P, Tang K, Osborn TC. Rapid genome change in synthetic polyploids of Brassica and its implications for polyploid evolution. Proc Natl Acad Sci U S A 1995 ;92(17):7719-23.
31. Ziegler DJ, Khan D, Pulgar-Vidal N, Parkin IAP, Robinson SJ, Belmonte MF. Genomic asymmetry of the Brassica napus seed: epigenetic contributions of DNA methylation and small RNAs to subgenome bias. Plant J 2023;115(3):690-708.
32. Orantes-Bonilla M, Wang H, Lee HT, Golicz AA, Hu D, Li W, et al. Transgressive and parental dominant gene expression and cytosine methylation during seed development in Brassica napus hybrids. Theor Appl Genet 2023;136(5):113.
33. Shen Y, Sun S, Hua S, Shen E, Ye CY, Cai D, et al. Analysis of transcriptional and epigenetic changes in hybrid vigor of allopolyploid Brassica napus uncovers key roles for small RNAs. Plant J 2017;91(5):874-893.
34. Li Z, Li M, Wang J. Asymmetric subgenomic chromatin architecture impacts on gene expression in resynthesized and natural allopolyploid Brassica napus. Commun Biol 2022;5(1):762.
35. Leflon M, Grandont L, Eber F, Huteau V, Coriton O, Chelysheva L, et al. Crossovers get a boost in Brassica allotriploid and allotetraploid hybrids. Plant Cell 2010;22(7):2253-64.
36. Cao Y, Zhao K, Xu J, Wu L, Hao F, Sun M, et al. Genome balance and dosage effect drive allopolyploid formation in Brassica. Proc Natl Acad Sci U S A 2023;120(14):e2217672120.
37. Chalhoub B, Denoeud F, Liu S, Parkin IA, Tang H, Wang X, et al. Early allopolyploid evolution in the post-Neolithic Brassica napus oilseed genome. Science 2014;345(6199):950-3.
38. Lu K, Wei L, Li X, Wang Y, Wu J, Liu M, et al. Whole-genome resequencing reveals Brassica napus origin and genetic loci involved in its improvement. Nat Commun 2019;10(1):1154.
39. Cai X, Chang L, Zhang T, Chen H, Zhang L, Lin R, et al. Impacts of allopolyploidization and structural variation on intraspecific diversification in Brassica rapa. Genome Biol 2021;22(1):166.
40. Zhang L, Zou J, Li S, Wang B, Raboanatahiry N, Li M. Characterization and expression profiles of miRNAs in the triploid hybrids of Brassica napus and Brassica rapa. BMC Genomics 2019;20(1):649
41. Quan C, Chen G, Li S, Jia Z, Yu P, Tu J, et al. Transcriptome shock in interspecific F1 allotriploid hybrids between Brassica species. J Exp Bot 2022;73(8): 2336-53.
42. Qian W, Meng J, Li M, Frauen M, Sass O, Noack J, et al. Introgression of genomic components from Chinese Brassica rapa contributes to widening the genetic diversity in rapeseed (B. napus L.), with emphasis on the evolution of Chinese rapeseed. Theor Appl Genet 2006;113(1):49-54
43. Zou J, Fu D, Gong H, Qian W, Xia W, Pires JC, et al. De novo genetic variation associated with retrotransposon activation, genomic rearrangements and trait variation in a recombinant inbred line population of Brassica napus derived from interspecific hybridization with Brassica rapa. Plant J 2011;68(2):212-24.
44. Bolger AM, Lohse M, Usadel B. Trimmomatic: a flexible trimmer for Illumina sequence data. Bioinformatics 2014;30(15): 2114-20.
45. Song JM, Guan Z, Hu J, Guo C, Yang Z, Wang S, et al. Eight high-quality genomes reveal pan-genome

architecture and ecotype differentiation of Brassica napus. Nat Plants 2020;6(1):34-45.
46. Langmead B, Salzberg SL. Fast gapped-read alignment with Bowtie 2. Nat Methods 2012;9(4): 357-9.
47. Danecek P, Bonfield JK, Liddle J, Marshall J, Ohan V, Pollard MO, et al. Twelve years of SAMtools and BCFtools. Gigascience. 2021,10(2):giab008. doi: 10.1093/gigascience/giab008
48. Zhang Y, Liu T, Meyer CA, Eeckhoute J, Johnson DS, Bernstein BE, et al. Model-based Analysis of ChIP-Seq (MACS). Genome Biol 2008;9(9): R137.
49. Yu G, Wang LG, He QY. ChIPseeker: an R/Bioconductor package for ChIP peak annotation, comparison and visualization. Bioinformatics 2015;31(14): 2382-3.
50. Ross-Innes CS, Stark R, Teschendorff AE, Holmes KA, Ali HR, Dunning MJ, et al. Differential oestrogen receptor binding is associated with clinical outcome in breast cancer. Nature 2012;481(7384): 389-93.
51. Heinz S, Benner C, Spann N, Bertolino E, Lin YC, Laslo P, et al. Simple combinations of lineage-determining transcription factors prime cis-regulatory elements required for macrophage and B cell identities. Mol cell 2010;38(4): 576-89.
52. Zhou Q, Lim JQ, Sung WK, Li G. An integrated package for bisulfite DNA methylation data analysis with indel-sensitive mapping. Bmc Bioinformatics 2019;20(1): 47.
53. Kim D, Landmead B, Salzberg SL. HISAT: a fast spliced aligner with low memory requirements. Nat Methods 2015;12(4): 357-60.
54. Pertea M, Pertea GM, Antonescu CM, Chang TC, Mendell JT, Salzberg SL. StringTie enables improved reconstruction of a transcriptome from RNA-seq reads. Nat Biotechnol 2015;33(3): 290-5.
55. Love MI, Huber W, Anders S. Moderated estimation of fold change and dispersion for RNA-seq data with DESeq2. Genome Biol 2014;15(12):550.
56. Johnson NR, Yeoh JM, Coruh C, Axtell MJ. Improved placement of multi-mapping small RNAs. G3 (Bethesda) 2016;6(7): 2103-11.
57. Gao Q, Luo H, Li Y, Liu Z, Kang C. Genetic modulation of RAP alters fruit coloration in both wild and cultivated strawberry. Plant Biotechnol J 2020;18(7):1550-1561.
58. Wu Q, Meng YT, Feng ZH, Shen RF, Zhu XF. The endo-beta mannase MAN7 contributes to cadmium tolerance by modulating root cell wall binding capacity in Arabidopsis thaliana. J Integr Plant Biol 2023;65(7): 1670-86.
59. Shi X, Zhang C, Ko DK, Chen ZJ. Genome-Wide Dosage-Dependent and -Independent Regulation Contributes to Gene Expression and Evolutionary Novelty in Plant Polyploids. Mol Biol Evol 2015;32(9):2351-66.
60. Lu FH, McKenzie N, Gardiner LJ, Luo MC, Hall A, Bevan MW. Reduced chromatin accessibility underlies gene expression differences in homologous chromosome arms of diploid Aegilops tauschii and hexaploid wheat. GigaScience 2020;9(6): giaa070.
61. Wang P, Gu M, Yu X, Shao S, Du J, Wang Y, et al. Allele-specific expression and chromatin accessibility contribute to heterosis in tea plants (Camellia sinensis). Plant J 2022;112(5):1194-1211.
62. Zhang Y, Li Z, Liu J, Zhang Y, Ye L, Peng Y, et al. Transposable elements orchestrate subgenome-convergent and -divergent transcription in common wheat. Nat Commun 2022;13(1):6940.
63. Bousios A, Diez CM, Takuno S, Bystry V, Darzentas N, Gaut BS. A role for palindromic structures in the cis-region of maize Sirevirus LTRs in transposable element evolution and host epigenetic response. Genome Res 2016;26(2):226-37.
64. Yoo MJ, Liu X, Pires JC, Soltis PS, Soltis DE. Nonadditive gene expression in polyploids. Annu Rev Genet 2014;48: 485-517.
65. Elumalai RP, Nagpal P, Reed JW. A mutation in the Arabidopsis KT2/KUP2 potassium transporter gene affects shoot cell expansion. Plant Cell 2002;14(1):119-31.
66. Ahn SJ, Shin R, Schachtman DP. Expression of KT/KUP genes in Arabidopsis and the role of root hairs in K+

uptake. Plant Physiol 2004;134(3):1135-45.
67. Hu S, Du B, Mu G, Jiang Z, Li H, Song Y, et al. The transcription factor OsbZIP48 governs rice responses to zinc deficiency. Plant Cell Environ 2024;47(5): 1526-42.
68. Baldauf JA, Liu M, Vedder L, Yu P, Piepho HP, Schoof H, et al. Single-parent expression complementation contributes to phenotypic heterosis in maize hybrids. Plant Physiol. 2022;189(3):1625-1638.
69. Chen WH, Hsu CY, Cheng HY, Chang H, Chen HH, Ger MJ. Downregulation of putative UDP-glucose: flavonoid 3-O-glucosyltransferase gene alters flower coloring in Phalaenopsis. Plant Cell Rep 2011;30(6): 1007-17.
70. Wang H, Su B, Butts IAE, Dunham RA, Wang X. Chromosome-level assembly and annotation of the blue catfish Ictalurus furcatus, an aquaculture species for hybrid catfish reproduction, epigenetics, and heterosis studies. Gigascience 2022;11:giac070.
71. Sinha P, Singh VK, Saxena RK, Kale SM, Li Y, Garg V, et al. 2020. Genome-wide analysis of epigenetic and transcriptional changes associated with heterosis in pigeonpea. Plant Biotechnol J 2020;18(8):1697-710.
72. Tan C, Pan Q, Cui C, Xiang Y, Ge X, Li Z. Genome-Wide Gene/Genome Dosage Imbalance Regulates Gene Expressions in Synthetic Brassica napus and Derivatives (AC, AAC, CCA, CCAA). Front Plant Sci 2016;7:1432.
73. Zhang L, He J, He H, Wu J, Li M. Genome-wide unbalanced expression bias and expression level dominance toward Brassica oleracea in artificially synthesized intergeneric hybrids of Raphanobrassica. Hortic Res 2021;8(1):246.
74. Liu J, Li M, Zhang Q, Wei X, Huang X. Exploring the molecular basis of heterosis for plant breeding. J Integr Plant Biol 2020;62(3):287-298.
75. Schübeler D. Function and information content of DNA methylation. Nature. 2015;517(7534):321-6.
76. Sun Z, Wang Y, Song Z, Zhang H, Wang Y, Liu K, et al. DNA methylation in transposable elements buffers the connection between three-dimensional chromatin organization and gene transcription upon rice genome duplication. J Adv Res 2022;42:41-53.
77. Ziller MJ, Gu H, Müller F, Donaghey J, Tsai LT, Kohlbacher O, et al. Charting a dynamic DNA methylation landscape of the human genome. Nature 2013;500(7463):477-81.
78. Weinberg-Shukron A, Ben-Yair R, Takahashi N, Dunjić M, Shtrikman A, Edwards CA, Ferguson-Smith AC, Stelzer Y. Balanced gene dosage control rather than parental origin underpins genomic imprinting. Nat Commun 2022;13(1):4391.
79. Liu Q, Ma X, Li X, Zhang X, Zhou S, Xiong L, et al. Paternal DNA methylation is remodeled to maternal levels in rice zygote. Nat Commun 2023;14(1):6571.
80. Wu J, Huang B, Chen H, Yin Q, Liu Y, Xiang Y, et al. The landscape of accessible chromatin in mammalian preimplantation embryos. Nature 2016;534(7609):652-7.
81. Zhang Q, Guan P, Zhao L, Ma M, Xie L, Li Y, et al. Asymmetric epigenome maps of subgenomes reveal imbalanced transcription and distinct evolutionary trends in Brassica napus. Mol Plant 2021;14(4):604-19.
82. Majka M, Janáková E, Jakobson I, Järve K, Cápal P, Korchanová Z, et al. The chromatin determinants and Ph1 gene effect at wheat sites with contrasting recombination frequency. J Adv Res 2023;53: 75-85.
83. Bell JSK, Vertino PM. Orphan CpG islands define a novel class of highly active enhancers. Epigenetics. 2017;12(6):449-464.
84. Cao S, Chen K, Lu K, Chen S, Zhang X, Shen C, et al. Asymmetric variation in DNA methylation during domestication and de-domestication of rice. Plant Cell 2023;35(9):3429-3443.
85. Fang C, Yang M, Tang Y, Zhang L, Zhao H, Ni H, et al. Dynamics of cis-regulatory sequences and transcriptional divergence of duplicated genes in soybean. Proc Natl Acad Sci U S A 2023;120(44):e2303836120.
86. Quan C, Zhang Q, Zhang X, Chai K, Cheng G, Ma C, et al. Supporting data for "Interspecific hybridization in Brassica species leads to changes in agronomic traits through the regulation of gene expression by chromatin

accessibility and DNA methylation" GigaScience Database. 2025. https://doi.org/10.5524/102668.

Supporting Information

Supplemental materials and methods

The details of ATAC-seq, WGBS, RNA-seq, sRNA-seq experiment, and statistics methods were described in supplemental materials.

Supplemental figure legends

Figure S1. Determination of primary metabolites.

Figure S2. Quality control of ATAC-seq and RNA-seq datasets.

Figure S3. Genome browser showing ATAC-seq signals in the 192-kb and 259-kb region in two hybrids and their parents. Tracks show normalized values of three biological replicates for each sample.

Figure S4. Distribution of accessible chromatin regions (ACRs).

Figure S5. Distribution of novel ACRs.

Figure S6. Additive genes in two hybrids.

Figure S7. GO enrichment of transgressive up-regulated genes in Hybrid-sh.

Figure S8. Characterization of SPA-ACRs in F_1 hybrids.

Figure S9. The heatmap showed the expression levels of anthocyanin biosynthesis genes and the related transcription factors in F_1 hybrids and their parents. The colored boxes represent the gene expression heatmap, normalized by log_2FPKM values obtained from RNA-seq.

Figure S10. Quality control of WGBS and sRNA-seq datasets.

Figure S11. Differences in methylation levels between F_1 hybrids and their parental lines.

Figure S12. Characterization of DMRs and DMLs in F_1 hybrids.

Figure S13. Density of TEs and sRNAs in DMRs.

Figure S14. Dose-dependent and dose-independent distributions in the A and C subgenomes.

Supplemental Tables

Table S1. Primer sequences used for RT-qPCR analyses.

Table S2. Statistics of RNA-seq data and reads mapping for all samples.

Table S3. Statistics of ATAC-seq data and reads mapping for all samples.

Table S4. GO enrichment analysis of conservative additive genes in two hybrids.

Table S5. GO enrichment analysis of non-additively up-regulated genes in Hybrid-sh.

Table S6. GO enrichment analysis of non-additively up-regulated genes in Hybrid-yh.

Table S7. GO enrichment analysis of shared down-expressed genes in two hybrids.

Table S8. GO enrichment analysis of shared up-expressed genes in two hybrids.

Table S9. The contents of 16 ionic components in the F_1 hybrids and their parents.

Table S10. Statistics of WGBS data and reads mapping for all samples.

Table S11. Statistics of sRNA-seq data and reads mapping for all samples.

Figure legends

Figure 1. Overview of chromatin accessibility in the F_1 hybrids and their parental.

(a) The images showed the phenotypes of the allotriploid F_1 hybrids and their parents at the flowering stage (scale bars=3 cm) and the typical chromosome number (Scale bars = 10 μm) of the allotriploid *Brassica* hybrids. **(b)** Chromatin accessibility around genes in the F_1 hybrids (Hybrid-sh and Hybrid-yh), and their relative parents (maternal parents: *s70* and *yu25*; paternal parent: Hort). **(c)** The bar graph showed the proportion of ACR regions in the genome of F_1 hybrids and their relative parents. **(d)** Comparison of gene expression and ACR intensity (left) and ACR distance (right). **(e)** Expression levels of genes associated with different types of ACRs. Boxplots showed the median (horizontal line). The upper and lower quartiles were the boundaries of the boxplots. The outlier was the data point outside the whiskers of the box plot. The Kruskal-Wallis test calculated the *p*-value (**$p < 0.01$; ***$p < 0.001$).

Figure 2. Distribution of ACR in F_1 hybrids and their parental lines.

(a) The graph showed the proportion of ACR regions in Genic (overlapping with a gene), Proximal [within 2 kb upstream or downstream of a gene, including transcription start site (TSS) and transcription termination site (TTS)], and Distal (more than 2 kb away from any gene) in the F1 hybrids and their parents. **(b)** Genomic distribution of ACRs in the two hybrids and their parents. The percentage of ACRs categorized as genic, proximal, and distal within different subgenomes (A and C). **(c)** The percentage of novel ACRs in genic, proximal, and distal. **(d)** The pie chart showed the proportion of TE-derived novel ACRs (TE-driven ACRs when more than 50% of the region overlaps with TEs). **(e)** Boxplots showed the expression levels of genes associated with non-TE-mediated ACRs and TE-mediated ACRs. The Kruskal-Wallis test calculated the *p*-value (**$p < 0.01$). **(f)** The MA-plot showed differentially expressed ACRs (DE-ACRs) between Hybrid-sh and Hybrid-yh. **(g)** Pie charts showing the distribution of DE-ACRs in genic, proximal, and distal.

Figure 3. Non-additive genes in F_1 hybrids.

(a) The MA-plot showed differentially expressed genes (DEGs) between the F_1 hybrids and the mid-parent value (MPV). **(b)** Venn diagram showing the overlap of Hybrid-yh nonadditive down-regulated genes, nonadditive up-regulated genes, Hybrid-sh nonadditive down-regulated genes, nonadditive up-

regulated genes. **(c)** The scatterplot showed the distribution of differential expression levels of Hybrid-sh (left) and Hybrid-yh (right) versus maternal (*x*-axis) and paternal (*y*-axis) parents. **(d)** The graph showed the ACR densities of transgressive up-regulated (left) and down-regulated (right) genes. **(e)** Venn diagram showed an overlap of novel ACRs and transgressive up-regulated genes. **(f)** The genome browser showed ATAC-seq peaks around *Potassium Transporter 4* (*POT4*) in the parents and Hybrid-sh, and the enrichment of the *bZIP44* and *bZIP48* motif (ACGT) in the promoter region of *POT4*. **(g)** and **(h)**, the bar graph showed the expression level of *BnaA01.POT4* **(g)**, and potassium levels in Hybrid-sh and its relative parents **(h)**. Error bars indicated the mean ± SD of three biological replicates. In **(h)** and **(i)**, the student's *t*-test was used to calculate significance where ** and *** indicated $p < 0.01$ and $p < 0.001$, respectively.

Figure 4. A-subgenome SPA-ACR drives bZIP11 to promote UF3GT expression in paternal and Hybrid-sh.

(a) The graphs showed the *c*-means soft clustering analysis of chromatin accessibility levels of A-subgenomes in the Hybrid-sh and its relative parents. **(b)** The genome browser showed the ATAC-seq peaks around *BnaA06.UF3GT* in the parents and Hybrid-sh, and the bZIP11 motif (ACGT) enrichment in the promoter region of *BnaA06.UF3GT*. **(c)** The bar graph showed the expression level of *BnaA06.UF3GT* in the Hybrid-sh and its relative parents. **(d)** Schematic diagrams of the effector and reporter constructs for the dual-luciferase transcriptional activity assay. The bar graph showed the LUC/REN activities of Arabidopsis protoplasts after co-transformation with the (p*BnaA06.UF3GT:LUC*) and reporter constructs (p*35S* (control), and p*35S:BnaA03.bZIP11* (*BnaC03.bZIP11* and *BnaC01.bZIP11*)). **(e)** The EMSA results showed that BnaA03.bZIP11 and BnaC03.bZIP11 could directly bind to the *BnaA06.UF3GT* promoter. Increasing amounts (25- and 50-fold) of the unlabeled DNA fragments were added as competitors. The red arrow indicated the shift bands. Red letters indicate the mutated G-box cis-element within the *BnaA06.UF3GT* promoter. An asterisk indicates that the mutated cold competitor probes were added to the panel. **(f)** Anthocyanin biosynthesis pathway. The colored boxes were the gene expression heatmap from RNA-seq, normalized by $\log_2$FPKM (calculation method). Genes responsible for Leucocyanidin, Cyanidin, and Cyanidin-3-glucoside steps were shown in red. BnaPAL, phenylalanine ammonialyase; BnaC4H, cinnamic acid 4-hydroxylase; Bna4CL, 4-coumarate: coenzyme A ligase; BnaCHS, chalcone synthase;

BnaCHI, chalcone isomerase; BnaF3H, flavanone 3-hydroxylase; BnaF3′H, flavonoid 3′-hydroxylase; BnaDFR, dihydroflavonol-4-reductase; BnaANS, anthocyanidin synthase; BnaUF3GT, UDP glucose-flavonoid 3-O-glucosyltransferase. The colored boxes are the heatmap of gene expression from RNA-seq normalized by log_2FPKM. In **(h)** and **(i)**, error bars indicated the mean ± SD of three biological replicates. Student's *t*-test was used to calculate significance, with *** indicating $p < 0.001$.

Figure 5. Divergence of DNA methylation landscape between parents and hybrids.

(a) The boxplots showed the total DNA methylation level (CG, CHG, and CHH) of F_1 hybrids (Hybrid-sh (left) and Hybrid-yh (right)) and *in silico* hybrids (Wilcoxon rank-sum test; $^{**}p < 0.01$; $^{***}p < 0.001$; ns, no significant difference). **(b)** The bar graph showed the number of DMRs (Differentially Methylated Regions) between F_1 hybrids and *in silico* hybrids of 200 bp bins. Different colors indicate the distribution of DMRs in the distal (red), genic (green), and proximal (blue) regions. **(c)** The bar graph showed the number of genic (left) and proximal (right) DMGs (Differentially Methylated Genes) in the Hybrid-sh and Hybrid-yh. **(d)** Boxplots showed the expression levels of hyper- and hypo-DMGs in F_1 hybrids and *in silico* hybrids with methylation sites in proximal regions. The *y*-axis represented the gene expression level log_{10}(FPKM+1) (Wilcoxon rank-sum test; $^{**}p < 0.01$; $^{***}p < 0.001$; ns, no significant difference). **(e)** The boxplots showed the methylation level (CG, CHG, and CHH) across genes, TEs, and ACRs. **(f)** The pie chart showed the proportion of TE-derived and non-TE-derived ACRs (top) and genes (bottom). The TE-derived ACR was defined as having more than 50% of the region overlapping with TEs. **(g)** The image showed the methylation level (CG, CHG, and CHH) of TE ACRs, Distal ACRs, Genic ACRs, and Proximal ACRs. **(h)** The boxplots showed the expression level of genes that associated with methylated and non-methylated ACRs. **(i)** The genome browser showed DNA methylation loci around *BnaC02.PRL1* in F_1 hybrids (Hybrid-sh and Hybrid-yh) and their relative parents. In **(e)** and **(h)**, The upper and lower quartiles were boundaries of the boxplots. The Kruskal-Wallis test calculated the *p*-value ($^{**}p < 0.01$; $^{***}p < 0.001$).

Figure 6. Effects of genomic imbalance on accessible chromatin regions and DNA methylation in F_1 hybrids.

(a) The expression level (top) and number (bottom) of dose-dependent genes in the A and C-subgenomes. **(b)** The boxplots showed the proportion of dose-dependent homologous gene pairs and dose-dependent genome-unique genes in the A and C-subgenomes. **(c)** The graph showed the number

of dose-dependent and dose-independent homologous genes in the A and C-subgenomes. Pearson's correlation was used to test and divide all homologous genes into two groups based on the coefficient of determination (R^2) to compare the characteristics of dose-dependent and dose-independent homologous genes. Homologous genes with statistically significant correlations were designated as dose-dependent A (Ad) and C (Cd) genes, while those with in significant correlations were designated as dose-independent A (Ai) and C (Ci) genes. **(d)** The pie chart showed the percentage of AdCd, AdCi, AiCd, and AiCi duplicated genes. **(e)** and **(f)**, the images showed the ACR density **(e)** and DNA methylation level (CG, CHG, and CHH) **(f)** between AdCd, AdCi, AiCd, and AiCi duplicated genes. **(g)** and **(h)**, the images showed the distribution of DNA methylation level **(g)** and ACR density **(h)** of A and C-subgenomes in AACC and AAC.

Figure 7. Model of hybridization-induced variation of accessible chromatin regions and DNA methylation sites in F_1 hybrids.

F_1 hybrids inherit accessible chromatin regions (ACRs), sRNA, and DNA methylation loci from their parents, resulting in gene transgressive and single parental expressions (SPE). Silent ACR: ACRs were identified in parents but not in F_1 hybrids. Novel ACR: ACRs were identified in F_1 hybrids but not in parents. Transgressive up genes: The gene expression level of the F_1 hybrids was higher than that of the parents. Transgressive down genes: The gene expression level of F_1 hybrids was lower than that of the parents. SPE: Genes are expressed only in one parent and F1 hybrids but not in the other.

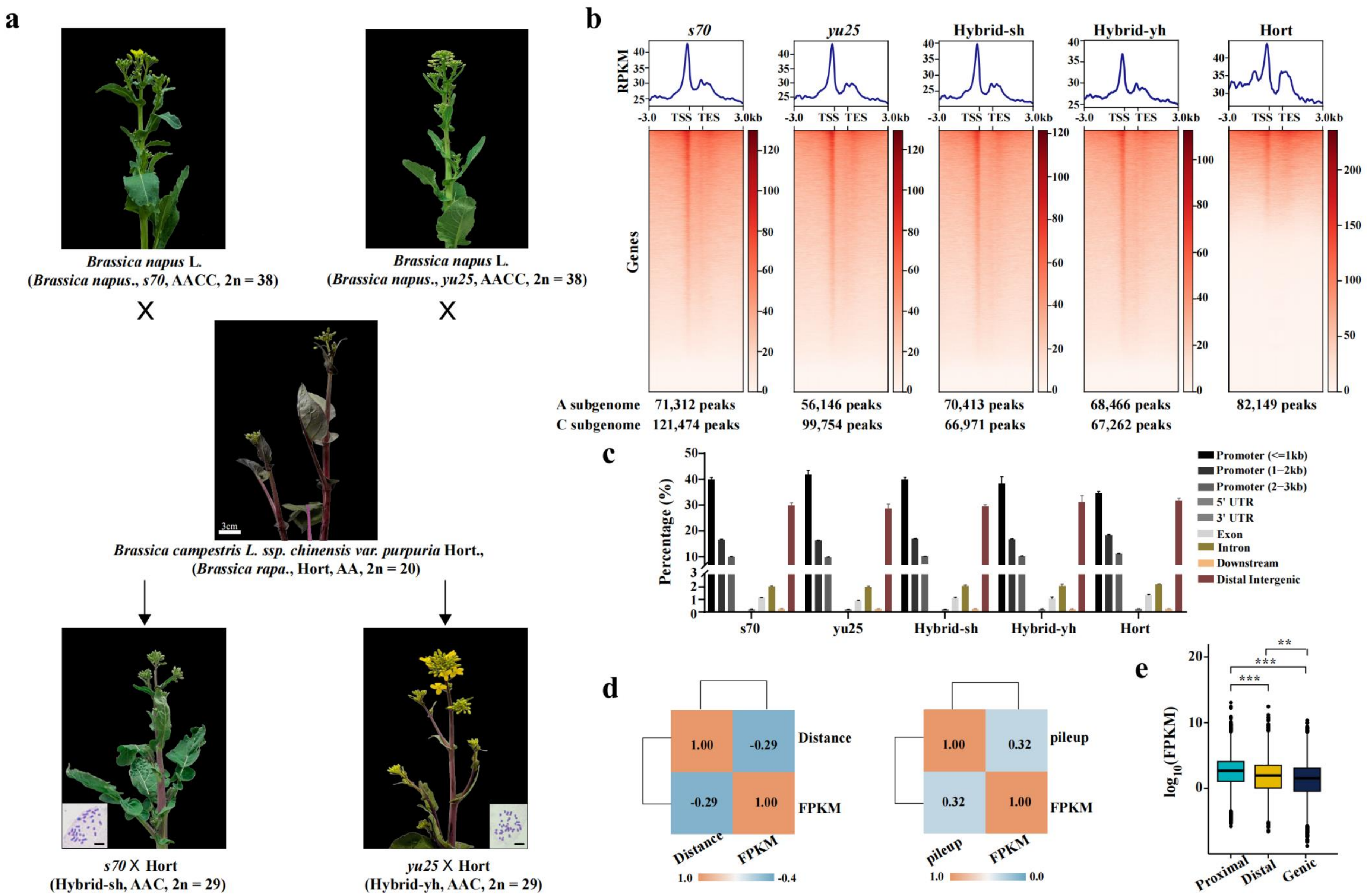

973

974 **Figure 1. Overview of chromatin accessibility in the F$_1$ hybrids and their parental.**

(a) The images showed the phenotypes of the allotriploid F_1 hybrids and their parents at the flowering stage (scale bars=3 cm) and the typical chromosome number (Scale bars = 10 µm) of the allotriploid *Brassica* hybrids. **(b)** Chromatin accessibility around genes in the F_1 hybrids (Hybrid-sh and Hybrid-yh), and their relative parents (maternal parents: *s70* and *yu25*; paternal parent: Hort). **(c)** The bar graph showed the proportion of ACR regions in the genome of F_1 hybrids and their relative parents. **(d)** Comparison of gene expression and ACR intensity (left) and ACR distance (right). **(e)** Expression levels of genes associated with different types of ACRs. Boxplots showed the median (horizontal line). The upper and lower quartiles were the boundaries of the boxplots. The outlier was the data point outside the whiskers of the box plot. The Kruskal-Wallis test calculated the *p*-value (**$p < 0.01$; ***$p < 0.001$).

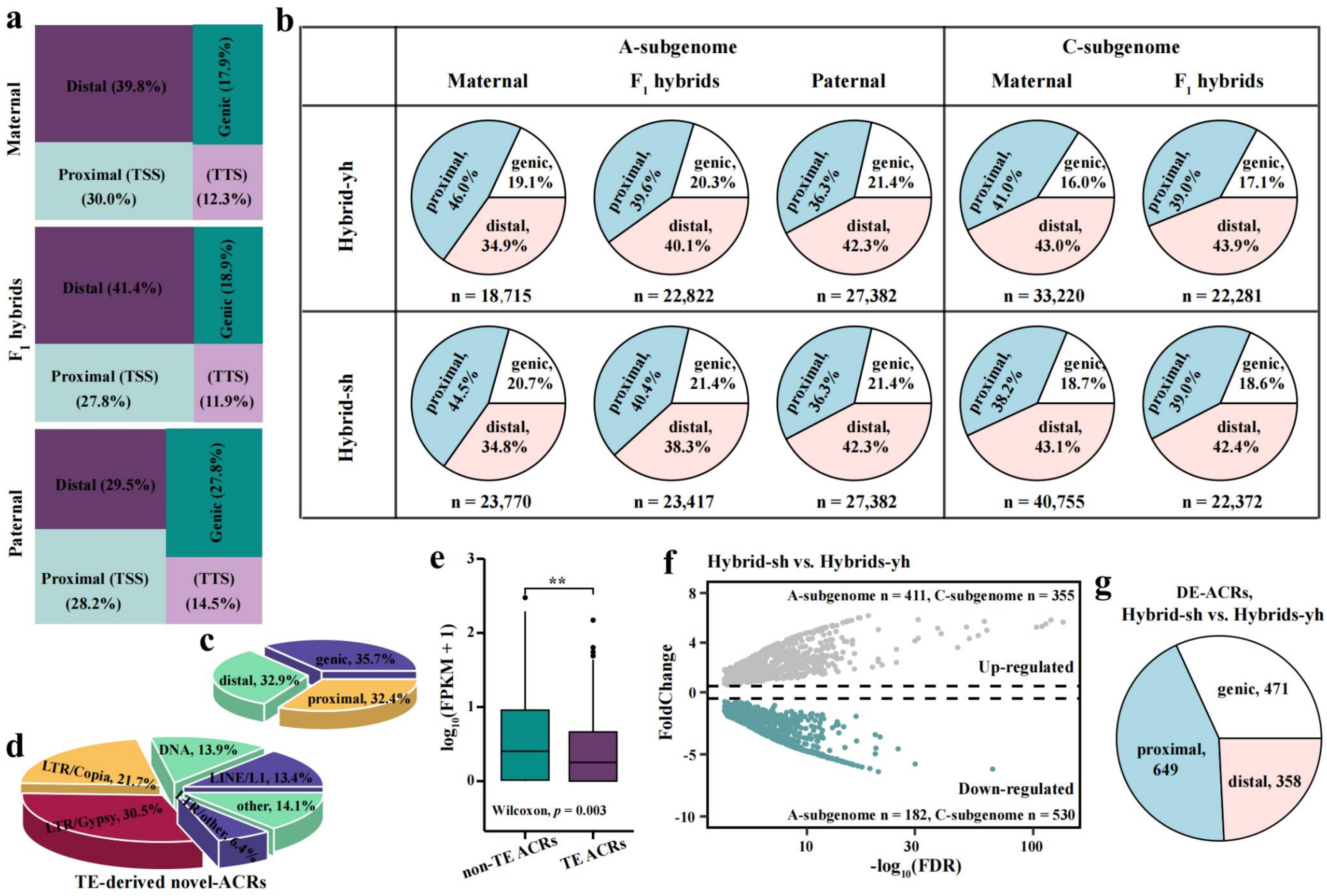
a
Maternal
Distal (39.8%)
Genic (17.9%)
Proximal (TSS) (30.0%)
(TTS) (12.3%)
F1 hybrids
Distal (41.4%)
Genic (18.9%)
Proximal (TSS) (27.8%)
(TTS) (11.9%)
Paternal
Distal (29.5%)
Genic (27.8%)
Proximal (TSS) (28.2%)
(TTS) (14.5%)
b
A-subgenome
C-subgenome
Maternal
F1 hybrids
Paternal
Hybrid-yh
proximal, 46.0%
genic, 19.1%
distal, 34.9%
n = 18,715
proximal, 39.6%
genic, 20.3%
distal, 40.1%
n = 22,822
proximal, 36.3%
genic, 21.4%
distal, 42.3%
n = 27,382
proximal, 41.0%
genic, 16.0%
distal, 43.0%
n = 33,220
proximal, 39.0%
genic, 17.1%
distal, 43.9%
n = 22,281
Hybrid-sh
proximal, 44.5%
genic, 20.7%
distal, 34.8%
n = 23,770
proximal, 40.4%
genic, 21.4%
distal, 38.3%
n = 23,417
proximal, 36.3%
genic, 21.4%
distal, 42.3%
n = 27,382
proximal, 38.2%
genic, 18.7%
distal, 43.1%
n = 40,755
proximal, 39.0%
genic, 18.6%
distal, 42.4%
n = 22,372
c
genic, 35.7%
distal, 32.9%
proximal, 32.4%
d
LTR/Copia, 21.7%
DNA, 13.9%
LINE/L1, 13.4%
other, 14.1%
LTR/other, 6.4%
LTR/Gypsy, 30.5%
TE-derived novel-ACRs
e
log10(FPKM + 1)
**
Wilcoxon, p = 0.003
non-TE ACRs
TE ACRs
f
Hybrid-sh vs. Hybrids-yh
A-subgenome n = 411, C-subgenome n = 355
Up-regulated
Down-regulated
A-subgenome n = 182, C-subgenome n = 530
FoldChange
-log10(FDR)
g
DE-ACRs, Hybrid-sh vs. Hybrids-yh
genic, 471
proximal, 649
distal, 358

982

Figure 2. Distribution of ACR in F_1 hybrids and their parental lines.

(a) The graph showed the proportion of ACR regions in Genic (overlapping with a gene), Proximal [within 2 kb upstream or downstream of a gene, including transcription start site (TSS) and transcription termination site (TTS)], and Distal (more than 2 kb away from any gene) in the F1 hybrids and their parents. **(b)** Genomic distribution of ACRs in the two hybrids and their parents. The percentage of ACRs categorized as genic, proximal, and distal within different subgenomes (A and C). **(c)** The percentage of novel ACRs in genic, proximal, and distal. **(d)** The pie chart showed the proportion of TE-derived novel ACRs (TE-driven ACRs when more than 50% of the region overlaps with TEs). **(e)** Boxplots showed the expression levels of genes associated with non-TE-mediated ACRs and TE-mediated ACRs. The Kruskal-Wallis test calculated the *p*-value (** $p < 0.01$). **(f)** The MA-plot showed differentially expressed ACRs (DE-ACRs) between Hybrid-sh and Hybrid-yh. **(g)** Pie charts showing the distribution of DE-ACRs in genic, proximal, and distal.

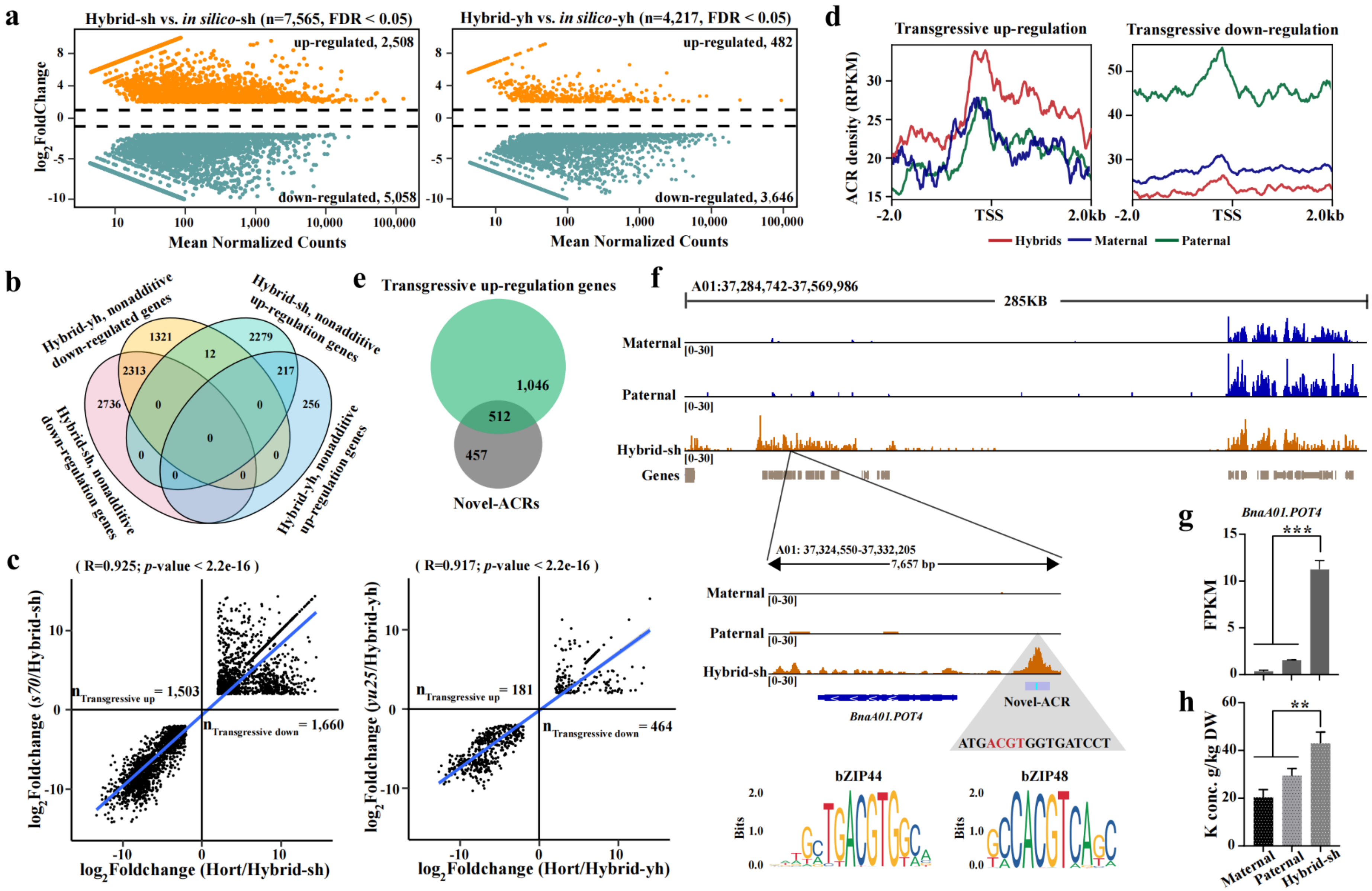
a
Hybrid-sh vs. in silico-sh (n=7,565, FDR < 0.05)
up-regulated, 2,508
down-regulated, 5,058
Hybrid-yh vs. in silico-yh (n=4,217, FDR < 0.05)
up-regulated, 482
down-regulated, 3,646
log2FoldChange
Mean Normalized Counts
b
Hybrid-yh, nonadditive down-regulated genes
Hybrid-sh, nonadditive up-regulation genes
Hybrid-sh, nonadditive down-regulation genes
Hybrid-yh, nonadditive up-regulation genes
c
(R=0.925; p-value < 2.2e-16)
(R=0.917; p-value < 2.2e-16)
n Transgressive up = 1,503
n Transgressive down = 1,660
n Transgressive up = 181
n Transgressive down = 464
log2 Foldchange (s70/Hybrid-sh)
log2 Foldchange (yu25/Hybrid-yh)
log2 Foldchange (Hort/Hybrid-sh)
log2 Foldchange (Hort/Hybrid-yh)
d
Transgressive up-regulation
Transgressive down-regulation
ACR density (RPKM)
TSS
Hybrids
Maternal
Paternal
e
Transgressive up-regulation genes
1,046
512
457
Novel-ACRs
f
A01:37,284,742-37,569,986
285KB
Maternal
Paternal
Hybrid-sh
Genes
A01: 37,324,550-37,332,205
7,657 bp
BnaA01.POT4
Novel-ACR
ATGACGTGGTGATCCT
bZIP44
bZIP48
Bits
g
BnaA01.POT4
FPKM
h
K conc. g/kg DW
Maternal
Paternal
Hybrid-sh

Figure 3. Non-additive genes in F_1 hybrids.

(a) The MA-plot showed differentially expressed genes (DEGs) between the F_1 hybrids and the mid-parent value (MPV). **(b)** Venn diagram showing the overlap of Hybrid-yh nonadditive down-regulated genes, nonadditive up-regulated genes, Hybrid-sh nonadditive down-regulated genes, nonadditive up-regulated genes. **(c)** The scatterplot showed the distribution of differential expression levels of Hybrid-sh (left) and Hybrid-yh (right) versus maternal (*x*-axis) and paternal (*y*-axis) parents. **(d)** The graph showed the ACR densities of transgressive up-regulated (left) and down-regulated (right) genes. **(e)** Venn diagram showed an overlap of novel ACRs and transgressive up-regulated genes. **(f)** The genome browser showed ATAC-seq peaks around *Potassium Transporter 4* (*POT4*) in the parents and Hybrid-sh, and the enrichment of the *bZIP44* and *bZIP48* motif (ACGT) in the promoter region of *POT4*. **(g)** and **(h)**, the bar graph showed the expression level of *BnaA01.POT4* **(g)**, and potassium levels in Hybrid-sh and its relative parents **(h)**. Error bars indicated the mean ± SD of three biological replicates. In **(h)** and **(i)**, the student's *t*-test was used to calculate significance where ** and *** indicated $p < 0.01$ and $p < 0.001$, respectively.

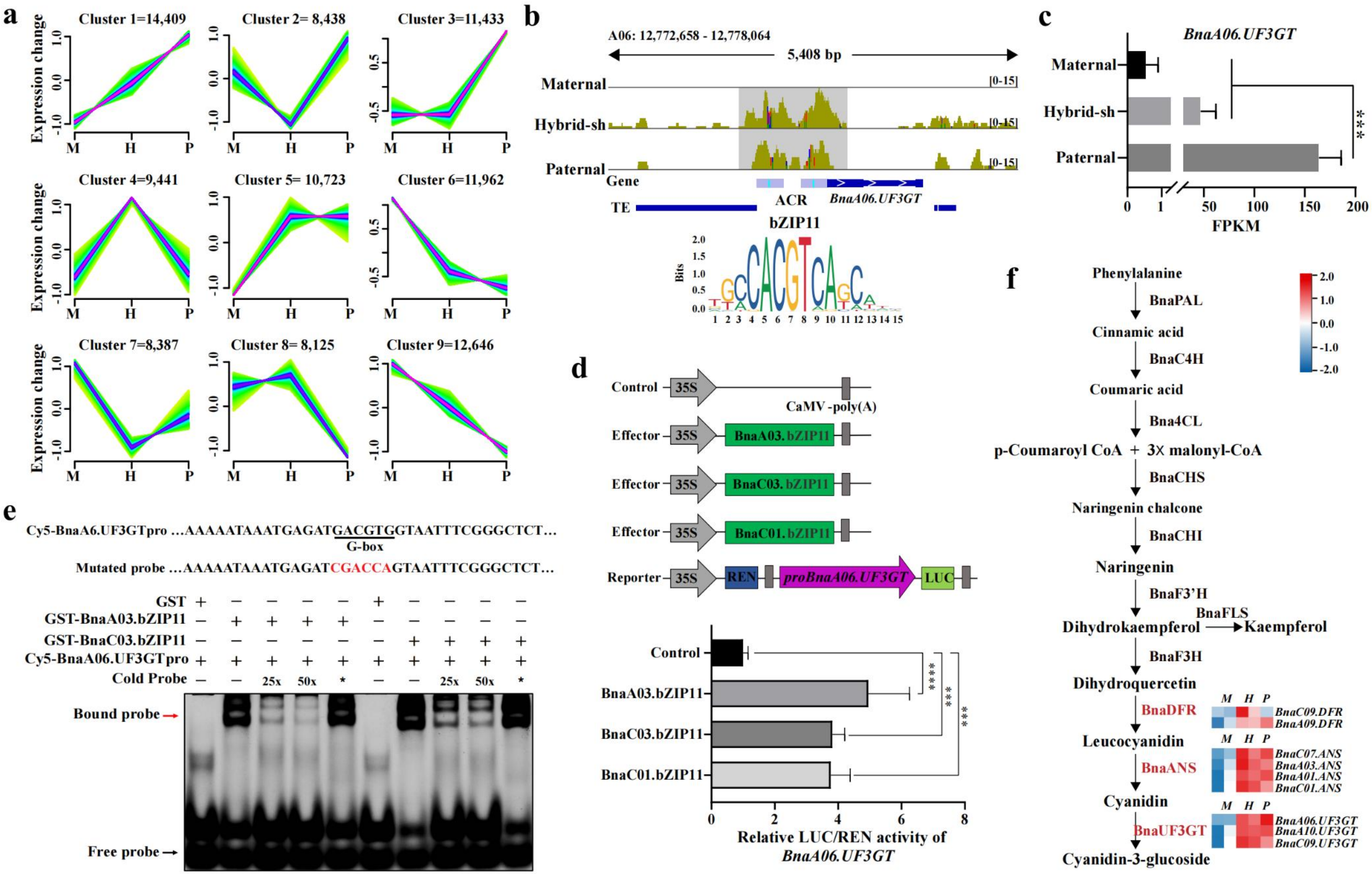

a
Cluster 1=14,409
Cluster 2= 8,438
Cluster 3=11,433
Cluster 4=9,441
Cluster 5= 10,723
Cluster 6=11,962
Cluster 7=8,387
Cluster 8= 8,125
Cluster 9=12,646
Expression change
M H P
b
A06: 12,772,658 - 12,778,064
5,408 bp
Maternal
Hybrid-sh
Paternal
Gene
TE
ACR
BnaA06.UF3GT
bZIP11
c
BnaA06.UF3GT
Maternal
Hybrid-sh
Paternal
FPKM
d
Control
Effector
Reporter
35S
CaMV -poly(A)
BnaA03.bZIP11
BnaC03.bZIP11
BnaC01.bZIP11
REN
proBnaA06.UF3GT
LUC
Relative LUC/REN activity of BnaA06.UF3GT
e
Cy5-BnaA6.UF3GTpro ...AAAAATAAATGAGATGACGTGGTAATTTCGGGCTCT...
G-box
Mutated probe ...AAAAATAAATGAGATCGACCAGTAATTTCGGGCTCT...
GST
GST-BnaA03.bZIP11
GST-BnaC03.bZIP11
Cy5-BnaA06.UF3GTpro
Cold Probe
Bound probe
Free probe
f
Phenylalanine
BnaPAL
Cinnamic acid
BnaC4H
Coumaric acid
Bna4CL
p-Coumaroyl CoA + 3X malonyl-CoA
BnaCHS
Naringenin chalcone
BnaCHI
Naringenin
BnaF3'H
Dihydrokaempferol
BnaFLS
Kaempferol
BnaF3H
Dihydroquercetin
BnaDFR
BnaC09.DFR
BnaA09.DFR
Leucocyanidin
BnaANS
BnaC07.ANS
BnaA03.ANS
BnaA01.ANS
BnaC01.ANS
Cyanidin
BnaUF3GT
BnaA06.UF3GT
BnaA10.UF3GT
BnaC09.UF3GT
Cyanidin-3-glucoside

Figure 4. A-subgenome SPA-ACR drives bZIP11 to promote UF3GT expression in paternal and Hybrid-sh.

(a) The graphs showed the *c*-means soft clustering analysis of chromatin accessibility levels of A-subgenomes in the Hybrid-sh and its relative parents. **(b)** The genome browser showed the ATAC-seq peaks around *BnaA06.UF3GT* in the parents and Hybrid-sh, and the bZIP11 motif (ACGT) enrichment in the promoter region of *BnaA06.UF3GT*. **(c)** The bar graph showed the expression level of *BnaA06.UF3GT* in the Hybrid-sh and its relative parents. **(d)** Schematic diagrams of the effector and reporter constructs for the dual-luciferase transcriptional activity assay. The bar graph showed the LUC/REN activities of Arabidopsis protoplasts after co-transformation with the (p*BnaA06.UF3GT:LUC*) and reporter constructs (p*35S* (control), and p*35S:BnaA03.bZIP11* (*BnaC03.bZIP11* and *BnaC01.bZIP11*)). **(e)** The EMSA results showed that BnaA03.bZIP11 and BnaC03.bZIP11 could directly bind to the *BnaA06.UF3GT* promoter. Increasing amounts (25- and 50-fold) of the unlabeled DNA fragments were added as competitors. The red arrow indicated the shift bands. Red letters indicate the mutated G-box cis-element within the *BnaA06.UF3GT* promoter. An asterisk indicates that the mutated cold competitor probes were added to the panel. **(f)** Anthocyanin biosynthesis pathway. The colored boxes were the gene expression heatmap from RNA-seq, normalized by $\log_2$FPKM (calculation method). Genes responsible for Leucocyanidin, Cyanidin, and Cyanidin-3-glucoside steps were shown in red. BnaPAL, phenylalanine ammonialyase; BnaC4H, cinnamic acid 4-hydroxylase; Bna4CL, 4-coumarate: coenzyme A ligase; BnaCHS, chalcone synthase; BnaCHI, chalcone isomerase; BnaF3H, flavanone 3-hydroxylase; BnaF3′H, flavonoid 3′-hydroxylase; BnaDFR, dihydroflavonol-4-reductase; BnaANS, anthocyanidin synthase; BnaUF3GT, UDP glucose-flavonoid 3-O-glucosyltransferase. The colored boxes are the heatmap of gene expression from RNA-seq normalized by $\log_2$FPKM. In **(h)** and **(i)**, error bars indicated the mean ± SD of three biological replicates. Student's *t*-test was used to calculate significance, with *** indicating $p < 0.001$.

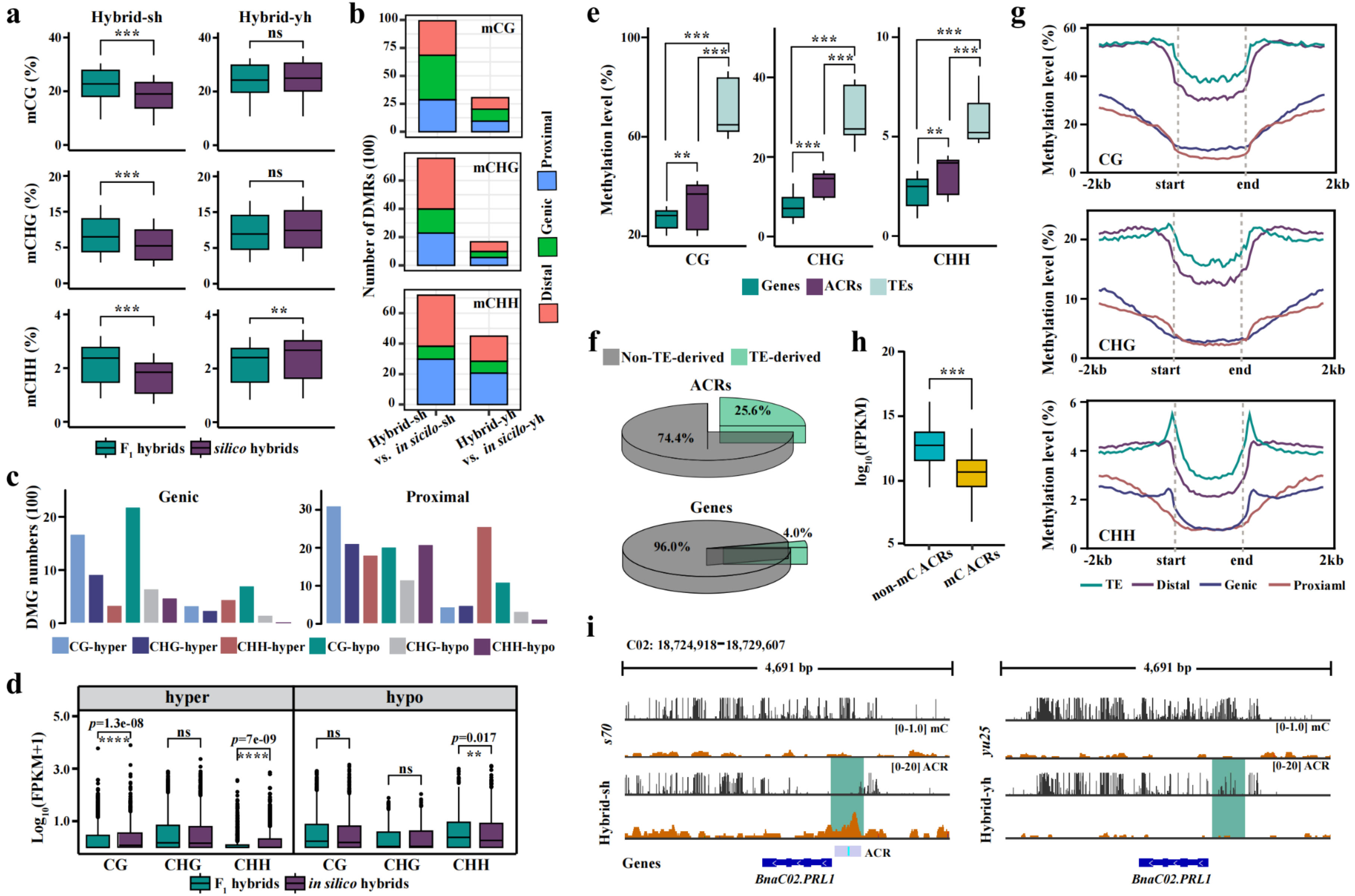

1020

1021 **Figure 5. Divergence of DNA methylation landscape between parents and hybrids.**

(a) The boxplots showed the total DNA methylation level (CG, CHG, and CHH) of F_1 hybrids (Hybrid-sh (left) and Hybrid-yh (right)) and *in silico* hybrids (Wilcoxon rank-sum test; $^{**}p < 0.01$; $^{***}p < 0.001$; ns, no significant difference). **(b)** The bar graph showed the number of DMRs (Differentially Methylated Regions) between F_1 hybrids and *in silico* hybrids of 200 bp bins. Different colors indicate the distribution of DMRs in the distal (red), genic (green), and proximal (blue) regions. **(c)** The bar graph showed the number of genic (left) and proximal (right) DMGs (Differentially Methylated Genes) in the Hybrid-sh and Hybrid-yh. **(d)** Boxplots showed the expression levels of hyper- and hypo-DMGs in F_1 hybrids and *in silico* hybrids with methylation sites in proximal regions. The *y*-axis represented the gene expression level $\log_{10}$(FPKM+1) (Wilcoxon rank-sum test; $^{**}p < 0.01$; $^{***}p < 0.001$; ns, no significant difference). **(e)** The boxplots showed the methylation level (CG, CHG, and CHH) across genes, TEs, and ACRs. **(f)** The pie chart showed the proportion of TE-derived and non-TE-derived ACRs (top) and genes (bottom). The TE-derived ACR was defined as having more than 50% of the region overlapping with TEs. **(g)** The image showed the methylation level (CG, CHG, and CHH) of TE ACRs, Distal ACRs, Genic ACRs, and Proximal ACRs. **(h)** The boxplots showed the expression level of genes that associated with methylated and non-methylated ACRs. **(i)** The genome browser showed DNA methylation loci around *BnaC02.PRL1* in F_1 hybrids (Hybrid-sh and Hybrid-yh) and their relative parents. In **(e)** and **(h)**, The upper and lower quartiles were boundaries of the boxplots. The Kruskal-Wallis test calculated the *p*-value ($^{**}p < 0.01$; $^{***}p < 0.001$).

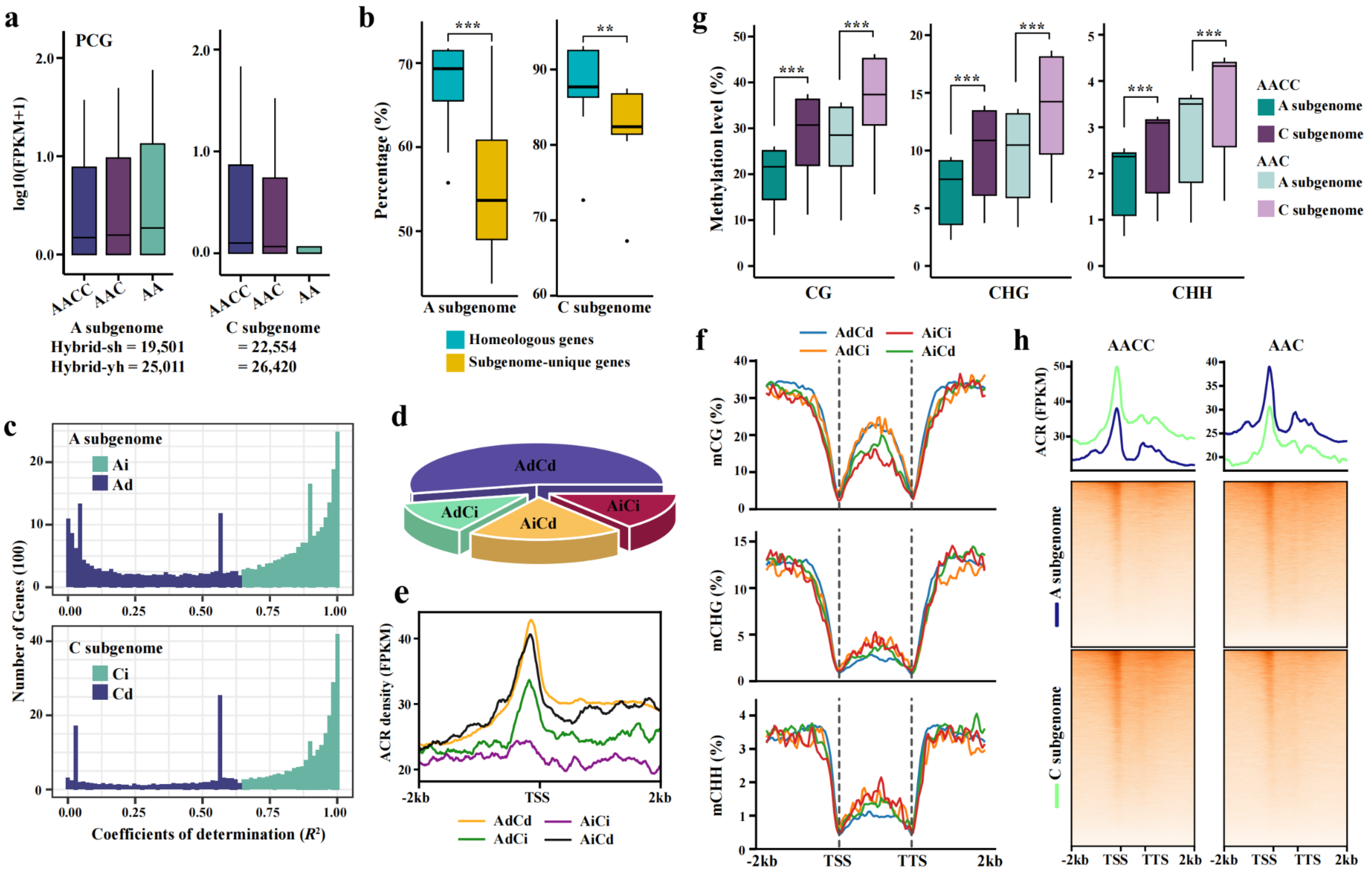
a
PCG
log10(FPKM+1)
AACC
AAC
AA
A subgenome
C subgenome
Hybrid-sh = 19,501
= 22,554
Hybrid-yh = 25,011
= 26,420
b
Percentage (%)
A subgenome
C subgenome
Homeologous genes
Subgenome-unique genes
g
Methylation level (%)
CG
CHG
CHH
AACC
A subgenome
C subgenome
AAC
A subgenome
C subgenome
c
A subgenome
Ai
Ad
C subgenome
Ci
Cd
Number of Genes (100)
Coefficients of determination (R^2)
d
AdCd
AdCi
AiCd
AiCi
e
ACR density (FPKM)
-2kb
TSS
2kb
AdCd
AiCi
AdCi
AiCd
f
AdCd
AdCi
AiCi
AiCd
mCG (%)
mCHG (%)
mCHH (%)
-2kb
TSS
TTS
2kb
h
AACC
AAC
ACR (FPKM)
A subgenome
C subgenome
-2kb
TSS
TTS
2kb

Figure 6. Effects of genomic imbalance on accessible chromatin regions and DNA methylation in F_1 hybrids.

(a) The expression level (top) and number (bottom) of dose-dependent genes in the A and C-subgenomes. **(b)** The boxplots showed the proportion of dose-dependent homologous gene pairs and dose-dependent genome-unique genes in the A and C-subgenomes. **(c)** The graph showed the number of dose-dependent and dose-independent homologous genes in the A and C-subgenomes. Pearson's correlation was used to test and divide all homologous genes into two groups based on the coefficient of determination (R^2) to compare the characteristics of dose-dependent and dose-independent homologous genes. Homologous genes with statistically significant correlations were designated as dose-dependent A (Ad) and C (Cd) genes, while those with in significant correlations were designated as dose-independent A (Ai) and C (Ci) genes. **(d)** The pie chart showed the percentage of AdCd, AdCi, AiCd, and AiCi duplicated genes. **(e)** and **(f)**, the images showed the ACR density **(e)** and DNA methylation level (CG, CHG, and CHH) **(f)** between AdCd, AdCi, AiCd, and AiCi duplicated genes. **(g)** and **(h)**, the images showed the distribution of DNA methylation level **(g)** and ACR density **(h)** of A and C-subgenomes in AACC and AAC.

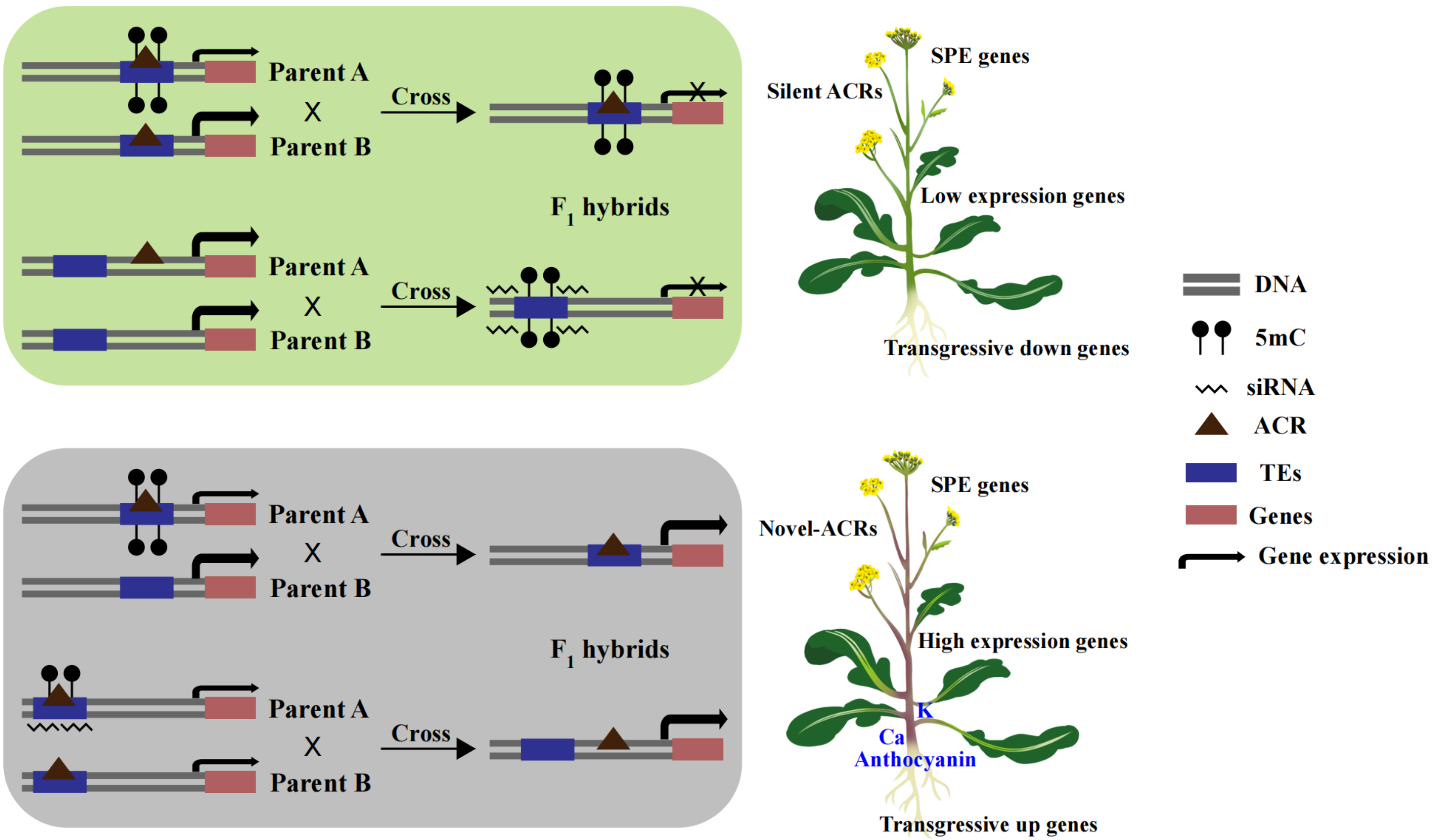

1046

1047 **Figure 7. Model of hybridization-induced variation of accessible chromatin regions and DNA methylation sites in F_1 hybrids.**

F_1 hybrids inherit accessible chromatin regions (ACRs), sRNA, and DNA methylation loci from their parents, resulting in gene transgressive and single parental expressions (SPE). Silent ACR: ACRs were identified in parents but not in F_1 hybrids. Novel ACR: ACRs were identified in F_1 hybrids but not in parents. Transgressive up genes: The gene expression level of the F_1 hybrids was higher than that of the parents. Transgressive down genes: The gene expression level of F_1 hybrids was lower than that of the parents. SPE: Genes are expressed only in one parent and F1 hybrids but not in the other.

Click here to access/download
Supplementary Material
Supplemental information-Final.docx

Cover letter
Click here to access/download;Personal Cover;Cover letter.docx

Dear GigaScience Editor,

We would like to thank you and the reviewers for your constructive feedback on our manuscript. Your insightful comments and guidelines for revision have been invaluable in improving the quality of our work. We now submit the revised manuscript titled "**Interspecific hybridization in *Brassica* species leads to changes in agronomic traits through the regulation of gene expression by chromatin accessibility and DNA methylation (GIGA-D-24-00365)**" by Quan et al. In response to the main comments and suggestions, we have made several significant improvements, providing more robust and quantitative evidence for our findings. We have also clarified our explanations and refined the language throughout.

1. Introduction and Discussion Revision: We have revised the background and discussion section with recent advances in accessible chromatin regions (ACRs), DNA methylation, and its role in gene regulation in species of the genus *Brassica* or other plants.

2. Data availability: We calculate the sequencing depth of ATAC-seq and demonstrate that the data has good reproducibility and high quality through IGV visualization.

3. Revision of Results: The Results section has been updated to include the differences in DNA methylation and ACR that arise from interspecific hybridization in the two hybrids. Additionally, we analyzed the distribution and shifts of ACRs in both the A and C subgenomes. The discussion also addresses the reasons for these observed differences.

In summary, we have clarified the results in the manuscript and discussed the relevant findings to address the important concerns and criticisms raised by the reviewers and the editor. Specific responses to each reviewer are also provided below. Major changes and improvements in the revised manuscript are highlighted in blue. We hope our revised manuscript can now be considered for publication in GigaScience.

Thank you for considering our submission.

With best wishes

Cheng Dai
