# Supplementary material for: Interspecific hybridization in Brassica species leads to changes in agronomic traits through the regulation of gene expression by chromatin accessibility and DNA methylation
Source: Gigascience. 2025 Apr 22;14:giaf029. doi: 10.1093/gigascience/giaf029 (PMC12012897; doi:10.1093/gigascience/giaf029)
Supplement: giaf029_Authors_Response_To_Reviewer_Comments_Original_Submission [file giaf029_authors_response_to_reviewer_comments_original_submission.pdf]

Author's Response To Reviewer Comments

Dear GigaScience Editor,

We would like to thank you and the reviewers for your constructive feedback on our manuscript. Your insightful comments and guidelines for revision have been invaluable in improving the quality of our work. We now submit the revised manuscript titled "Interspecific hybridization in Brassica species leads to changes in agronomic traits through the regulation of gene expression by chromatin accessibility and DNA methylation (GIGA-D-24-00365)" by Quan et al. In response to the main comments and suggestions, we have made several significant improvements, providing more robust and quantitative evidence for our findings. We have also clarified our explanations and refined the language throughout.
1. Introduction and Discussion Revision: We have revised the background and discussion section with recent advances in accessible chromatin regions (ACRs), DNA methylation, and its role in gene regulation in species of the genus Brassica or other plants.
2. Data availability: We calculate the sequencing depth of ATAC-seq and demonstrate that the data has good reproducibility and high quality through IGV visualization.
3. Revision of Results: The Results section has been updated to include the differences in DNA methylation and ACR that arise from interspecific hybridization in the two hybrids. Additionally, we analyzed the distribution and shifts of ACRs in both the A and C subgenomes. The discussion also addresses the reasons for these observed differences.
In summary, we have clarified the results in the manuscript and discussed the relevant findings to address the important concerns and criticisms raised by the reviewers and the editor. Specific responses to each reviewer are also provided below. Major changes and improvements in the revised manuscript are highlighted in blue. We hope our revised manuscript can now be considered for publication in GigaScience. Thank you for considering our submission.
With best wishes
Cheng Dai

Reviewer reports:

Reviewer #1: The manuscript presents a wealth of data on chromatin accessibility and DNA methylation during polyploidization, offering fresh insights into these processes in the context of interspecific hybridization. While the study provides valuable observations, several key issues must be addressed to strengthen the conclusions.
Major Points:
1. Data Quality Concerns: In Fig 1B, the Hort dataset appears to be of lower quality compared to the other datasets, which may affect some conclusions. Similarly, Figure 2d shows low enrichment levels, indicating potential issues with data quality. This could impact conclusions regarding ACR distribution and differences before and after allopolyploidization. The authors should carefully reconsider the conclusions drawn, especially regarding ACR calling.
Response: Thanks for your suggestions! The ATAC-seq peaks were enriched in the following samples: 192,786 in s70, 155,900 in yu25, 137,384 in Hybrid-sh, 135,728 in Hybrid-yh, and 82,149 in Hort (new Fig. 1b). The maternal lines (s70 and yu25) are the allotetraploid Brassica napus (AACC, 2n=38), the paternal line (Hort) is the diploid Brassica rapa (AA, 2n=20), and the F1 hybrid is an allotriploid hybrid (Hybrid-sh and Hybrid-yh; AAC, 2n=29). The ACR peaks in Hort were less than maternal lines and F1 hybrids. This could be due to the Hort lacking the C subgenome. We then compared the ACR peaks in different subgenomes in F1 hybrids and parental lines. The result showed that 71,312, 56,146, 70,413, 68,466, and 82,149 ACR peaks were identified in the A subgenome of s70, yu25, Hybrid-sh, Hybrid-yh, and Hort, respectively (new Fig. 1b). However, in the C subgenome, 121,474, 99,754, 66,971, and 67,262 ACR peaks were identified in s70, yu25, Hybrid-sh, and Hybrid-yh (new Fig. 1b), indicating that the ACR densities in the C-subgenome are suppressed by hybridization. We have revised the results by including the ACR peaks in different subgenomes. Please find the relative data in new Figure 1b, and the results in lines 212-223. To minimize the impact on the data from the reduction of ACR peaks caused by the deletion of the C subgenome in Hort, we analyzed the distribution of ACRs and identified novel ACRs following

interspecific hybridization by separately comparing the A and C subgenomes. For single-parent activation ACRs, we concentrated solely on the peaks associated with the A subgenome.
There are two main categories within non-additive genes: expression level dominance (ELD) and transgressive (Yoo et al. 2014). Based on gene expression level, the transgressive genes could be further categorized as transgressive up-regulated genes and transgressive down-regulated genes (Yoo et al. 2014). We have identified 3,163 and 645 transgressive expression genes in Hybrid-sh and Hybrid-yh, respectively (new Fig. 3c). In Hybrid-yh, the number of down-regulated genes (464) was significantly higher than the number of up-regulated genes (181) (new Fig. 3c). In comparison, no difference was observed between the number of up-regulated (1,503) and down-regulated (1,660) transgressive genes in Hybrid-sh (new Fig. 3c). The ACR densities of transgressive up-regulated genes in F1 hybrids were higher than parental lines (new Fig. 3d). In contrast, ACR densities of transgressive down-regulated genes in F1 hybrids were significantly lower than parental lines (new Fig. 3d). These results indicate that a significant correlation between the expression of transgressive genes and ACRs. We have revised Figure 3 and the results in lines 319-327.

2. Read Distribution in Parent Tracks: In Fig 2G, the two parent tracks seem to have no detectable reads. The authors should verify the sequencing depth and confirm whether it is sufficient for reliable data interpretation.
Response: Thanks for your suggestions! We have verified the average sequencing depth of ATAC-seq by samtools (depth -b). The results showed that the average sequencing depth of chromatin accessibility regions for s70, yu25, Hybrid-sh, Hybrid-yh, and Hort were 17, 18, 20, 19, and 17X values, respectively (new Fig.S3). The genome browser results showed that consistent ACR peaks were identified in three biological replicates of the parental line and F1 hybrids (new Fig.S3). We identified 969 novel ACRs and 25 silent ACRs in F1 hybrids (new Fig. S5a). Among them, novel ACRs in the A and C subgenomes were 209 and 760 (new Fig. S5b), respectively, indicating that the formation of novel ACRs in the C subgenome was more significant than in the A subgenome. For instance, in a window with a range of 7,657 bp, a novel ACR was identified in Hybrid-sh (new Fig. 3f). These results indicate that interspecific hybridization may lead to the activation of ACR in unopened regions (Wang et al. 2023; Han et al. 2022). We have revised Figures S3, S5, and Figure 3, and the results are in lines 212-215, 270-285, and 295-347.

3. Distribution and Shift of ACRs in the A and C Subgenomes: The authors conclude that interspecific hybridization induces changes in ACR distribution and abundance, especially in gene and proximal regions. However, it would be crucial to clarify how these changes differ between the A and C subgenomes. Are these changes biased toward one subgenome, or are they converging? The authors should compare their findings with studies such as the referenced paper on cotton hybrids (Ref. 36) and discuss the similarities and differences.
Response: Thanks for your suggestions! The parental lines s70, yu25, and Hort exhibited 23,770, 18,715, and 27,382 ACRs in the A subgenomes (new Fig. 2b). In contrast, 40,755 and 33,220 ACRs were identified in the C subgenomes of s70 and yu25, respectively (new Fig. 2b), showing the divergent distribution of ACRs between the A subgenome and the C subgenome. Significant differences in ACR distribution between subgenomes A and D were also found in G. hirsutum (AADD) (Han et al. 2022). We further analyzed whether there is immediate convergence in the distribution of different types of ACRs across subgenomes in interspecific hybrids. We further analyzed whether there is immediate convergence in the distribution of different types of ACRs (genic, proximal, and distal ACRs) across subgenomes in interspecific hybrids. There was no difference in genic ACR between the F1 hybrid and its parental A subgenome; however, significant differences were observed between proximal and distal ACRs (new Fig. 2b). This suggests that genic ACRs in the A subgenome converge immediately following interspecific hybridization. Notably, in the A subgenome, proximal ACRs were most abundant in the maternal line, followed by F1 hybrids, while the paternal line exhibited the least abundance (new Fig. 2b); in contrast, distal ACRs show the opposite trend (paternal > F1 hybrids > maternal) (new Fig. 2b). However, no significant differences were found in the proportions of the genic, proximal, and distal ACR in the C subgenome (new Fig. 2b), suggesting that the different types of ACRs converge immediately upon the halving of the C subgenome following interspecific hybridization. We have revised the new Figure 2; the results are in lines 248-268.

Minor Points:
1. The chromosome number diagram in Figure 1a lacks a scale bar, which should be included for reference.
Response: Corrected! Please find the new Fig. 1a

2. In Fig 2F, it would be useful to show the proportions of various TE classes across the genome for better context.
Response: Corrected! Please find the new Fig. 2d

3. The order of Hort distribution in Figure 2b should be made consistent for better comparison.
Response: Corrected! Please find the new Fig. 2a

4. The number of additively expressed genes is mentioned, but further explanation is needed regarding how these genes were identified and their biological significance.
Response: Thanks for your suggestions! To identify additive and nonadditive gene expression, we constructed independently in silico 'hybrids' by combining the RNA-seq data from sequenced parental individuals in a 1:1 ratio for the B. napus (s70 and yu25) and B. rapa (Hort) datasets. This ratio reflects the respective 1:2 genomic contribution of the parents to the F1 hybrids. The differentially expressed genes (DEGs) were identified by comparing the expression levels of genes between the hybrids and in silico hybrids, using the criteria of an adjusted p-value < 0.05 and a $|\log 2$ fold change$| \geq 1.5$. The results showed that the majority of expressed genes exhibited additive expression patterns in both Hybrid-sh (82.9%) and Hybrid-yh (90.1%), and more than 86% of the additive genes were conserved in the two hybrids (new Fig. 3a). GO enrichment analysis of conservative additive genes revealed enrichment for cellular process, rhythmic process, metabolic process, carbon-nitrogen utilization (new Table S4). This suggests that conserved additive genes are necessary for normal plant growth and development. We have revised the materials & methods and results sections in lines 164-171 and 295-318, respectively.

5. Clarify whether the enriched genes in Hybrid-yh are non-additively up-regulated, as the connection to gene regulation is unclear.
Response: Thanks for your suggestions! We identified 7,567 and 4,129 non-additively genes in Hybrid-sh and Hybrid-yh, respectively. In Hybrid-sh, there were 2,508 non-additively up-regulated genes and 5,058 non-additively down-regulated genes. In contrast, Hybrid-yh exhibited 482 non-additively up-regulated genes and 3,646 non-additively down-regulated genes. The non-additively up-regulated genes in Hybrid-sh were mainly associated with responses to biotic and abiotic stimuli, immune responses, and metabolic processes (new Table S5). Conversely, non-additively up-regulated genes in Hybrid-yh were enriched in ion transport, response to hormones, and metabolic processes (new Table S6). GO enrichment of non-additively up-regulated genes in F1 hybrids suggests an enhanced capacity for metabolite production and improved tolerance to environmental conditions. We have revised the new Figure 3; the results are in lines 294-318.

6. Replace "enrichment" with "GO enrichment" to ensure clarity in the text.
Response: Corrected!

7. The section title needs revision, as much of the content focuses on gene expression rather than chromatin changes.
Response: Thanks for your suggestions! We have revised the section title to “Nonadditive gene expression in F1 hybrids”.

8. The authors should cite relevant literature supporting the impact of LTRs and LINEs on gene regulation.
Response: Thanks for your suggestions! LTRs contribute to the silencing of active elements, affecting transposition potential and siRNA targeting levels and ultimately influencing the function of the element in the genome (Bousios et al. 2016). LTRs and LINE TEs in the < 2 kb promoter region of the gene decreased gene body chromatin accessibility and gene expression (Jordan et al. 2020). We have cited the relative research papers.

9. Provide more information on the distribution of novel ACRs and their overlap with TEs. It would be important to discuss whether this overlap influences gene expression.
Response: Thanks for your suggestions! It is a great idea to analyze the association between novel ACRs and their overlap with TEs. We identified 969 novel ACRs and 25 silent ACRs in F1 hybrids (new Fig. S5a). Among them, novel ACRs in the A and C subgenomes were 209 and 760, respectively (new Fig. S5b), indicating that the formation of novel ACRs in the C subgenome was more significant compared to the A

subgenome. The distribution of novel ACRs revealed that the proportions of ACRs in the genic, proximal, and distal were 35.7%, 32.4%, and 32.9%, respectively (new Fig. 2c). Furthermore, the chromosomal distribution of novel ACRs was mainly located at the telomeres, distal to the centromere (new Fig. S5c). 375 (38.7%) of these novel ACRs were classified as TE-driven ACRs, with LTR/Gypsy-type retrotransposons playing a prominent role (30.4%) compared to other types of TEs (new Fig. 2d). We found that gene expression in the proximal regions of non-TE-mediated ACRs was significantly higher than that of TE-mediated ACRs (new Fig. 2e). Transposable element (TE)-mediated ACRs may exhibit lower gene expression levels due to the influence of methylation on both the TE and its adjacent sequences. In contrast, non-TE-mediated ACRs are typically unaffected by transposable elements; consequently, DNA methylation levels in these regions may be reduced, thereby facilitating the expression of genes in neighboring areas. We have revised the results and discussions in lines 270-285.

10. The conclusion that hybrids tend to inherit specific sequences from the parent with higher methylation needs further explanation. How was this determined?
Response: Thanks for your suggestions! At the chromosomal level, a bias in the total methylation levels of CG, CHG, and CHH towards the hypermethylated parent was observed (new Fig. S11a). Further, the methylation levels of transposable element (TE) bodies in F1 hybrids and their parental lines across the whole genome were analyzed. We identified 25.9-30.6%, 35.0-40.2%, and 39.7-44.3% TEs in F1 hybrids CG, CHG, and CHH contexts, respectively, which were not significantly different from the maternal line (s70 and yu25) (new Fig. S11b). Compared with the paternal line (Hort), the proportions of TE no differences in CG, CHG, and CHH contexts that were observed in F1 hybrids were 13.5-13.9%, 19.6-20.0%, and 22.6-22.7% (new Fig. S11b). This indicates that the F1 hybrid tended to inherit higher methylation levels from the parent with higher methylation. We have revised the new Figure S11; the results are in lines 409-418.

11. The threshold used for dose-dependent and independent gene identification lacks a clear explanation. Please provide details on how these thresholds were determined.
Response: Thanks for your suggestions! The identification of dosage-dependent and independent genes was followed in the previous study (Shi et al. 2015). Briefly, Pearson correlation tests, employing the Benjamini and Hochberg false discovery rate (FDR) method, were conducted to assess the relationship between gene expression and genotype dosage for the A-subgenome (1/2 : 2/3 : 1) and C-subgenome (1/2 : 1/3 : 0) genes. The Pearson correlation and multiple test corrections were performed using the adjustment method in R. Genes exhibiting a significant correlation between expression and dosage (R2 > 0.64, FDR < 0.05) were classified as dosage-dependent. In contrast, those with no significant correlation were deemed dosage-independent. For instance, the Pearson correlation coefficient for genomic dosage (1/2 : 2/3 : 1) and gene expression values (1.72, 1.96, 2.54) of the gene BnaA01G0002000ZS is 0.99, with an R2 of 0.99, indicating that this gene is dosage-dependent. We have also revised the materials and methods section in lines 172-182.

Overall, this study is rich in data and offers valuable insights into epigenetic regulation during hybridization. However, addressing these issues will help to clarify the findings and improve the manuscript's impact.
Reviewer #2: This study investigates the construction of two hybrids through the crossing of two Brassica napus cultivars (s70, AsAsCsCs; yu25, AyAyCyCy, allotetraploid) with a Brassica rapa cultivar (B. campestris L. ssp. chinensis var. purpuria Hort.; AhAh, diploid). The research systematically explores accessible chromatin regions (ACRs), DNA methylation, and gene expression. Notable differences in the expression of non-additive genes and up-regulated transgressive genes associated with metal ion accumulation, as well as SPE genes linked to anthocyanin accumulation, were observed. Furthermore, the study reveals the role of ACRs and DNA methylation in regulating gene expression, including the impact of dosage effects. Before the article is accepted, the following revisions are recommended

1. The introduction is overly brief and lacks sufficient background information. It should include recent progress on accessible chromatin regions (ACRs), DNA methylation, and their role in gene regulation, especially in Brassica species or other crops. A clearer description of the research objectives and their potential implications would enhance the introduction.
Response: Thanks for your suggestions! We have revised the background section.

2. The differential expression of non-additive genes between Hybrid-sh and Hybrid-yh is reported, with

insights from GO-enrichment analysis. However, the authors should focus more on the shared gene expression patterns and provide a more in-depth discussion of how interspecies hybridization induces gene expression changes via ACRs and DNA methylation.
Response: Thanks for your suggestions! The 2,530 identical non-additive expressed genes (2,313 down-regulated and 217 up-regulated) were isolated in both Hybrid-sh and Hybrid-yh (new Fig. 3b). These down-regulated genes were associated with primary meristem tissue development, organic acid transmembrane transport, phloem transport, and auxin polar transport by GO enrichment analysis (new Table S7). In contrast, the up-regulated genes were enriched with response to external biotic stimulus, lipid metabolic process, and defense response pathways (new Table S8). We have revised the results in lines 312-318 and updated the discussion in lines 597-614.

3. The differences in DNA methylation and ACR patterns between the two hybrids (Hybrid-sh and Hybrid-yh) induced by interspecies hybridization need to be more clearly presented in the figures and text. Additionally, the authors should offer a detailed discussion of the reasons behind these differences.
Response: Thank you for your suggestions! We have analyzed differentially expressed accessible chromatin regions (DE-ACRs) in the two hybrids, identifying 1,478 DE-ACRs in Hybrid-yh compared to Hybrid-sh. Among these, 766 were differentially up-regulated ACRs, while 712 were differentially down-regulated (new Fig. 2f). Notably, the number of DE-ACRs in the C-subgenome (855) was significantly higher than that in the A-subgenome (593), illustrating a positive correlation between the number of DE-ACRs and genome size (new Fig. 2f). We further found that proximal DE-ACRs were the most numerous, followed by genic DE-ACRs, with distal DE-ACRs being the least prevalent. This indicates an uneven distribution of DE-ACRs across the genome (new Fig. 2g). Additionally, we identified 9,464 differentially methylated regions (DMRs) between Hybrid-sh and Hybrid-yh. In the context of Hybrid-sh, we recorded 2,370, 1,289, and 1,518 hyper-DMRs, along with 2,317, 1,256, and 714 hypo-DMRs in the CG, CHG, and CHH contexts, respectively (new Fig. S12b). Furthermore, regarding differential methylation loci (DMLs), we observed 5,323, 2,271, and 5,902 hyper-DMLs, as well as 5,448, 1,840, and 3,503 hypo-DMLs in the CG, CHG, and CHH contexts respectively, when compared to Hybrid-sh (new Fig. S12b). The differences in the number of hyper-DMRs (hyper-DMLs) and hypo-DMRs (hypo-DMLs) between the two hybrids stemmed from the DMRs (DMLs) in the CHH context. This data highlights the unbalanced changes in the levels of hyper- and hypo-CHH methylation between the two hybrids. We have updated the results in lines 286-293 and 437-444, as well as the discussion in lines 580-591.

4. In the section on "Accessible Chromatin Regions and DNA Methylation Differ in Genome Dosage Effects," the authors should provide a more thorough discussion in relation to previous studies on Brassica napus or other polyploid crops. This would help contextualize the findings and demonstrate how they align with or differ from existing knowledge.
Response: Thank you for your suggestions! In comparative studies examining gene expression patterns within polyploid plant species, it has been observed that most genes across different subgenomes exhibit coordinated expression, either in a dosage-dependent or dosage-independent manner (Shi et al. 2015; Tan et al. 2016). In the resynthesized allotetraploid Arabidopsis, approximately 56% of the alleles displayed congruent dosage expression patterns (Shi et al. 2015). Similar trends were noted in synthetic Brassica napus and its derivatives, where 58% of the A and C subgenome genes dosage expressed in the same direction (Tan et al. 2016). Our study on homologous genes from the A and C subgenomes in a related context further supports these findings, revealing that 66% of the genes exhibited coherent dosage expression, with 54% showing dosage-dependent behavior (AdCd) and 12% exhibiting dosage-independent behavior (AiCi) (new Fig. 6d). These results highlight the prevalence of both coordinated and independent regulatory mechanisms that influence gene expression in polyploid genomes. This underscores the importance of a nuanced understanding of the genetic and epigenetic factors that govern these expression patterns. We have updated the discussion in lines 540-560.

5. The discussion section currently lacks sufficient detail and does not adequately compare the results with previous studies. It would benefit from a deeper analysis of the novel insights provided by this study, as well as a discussion of any differences between the two hybrids.
Response: Thanks for your suggestions! We have revised the whole discussion section.

References
Shi X, Zhang C, Ko DK, Chen ZJ. Genome-wide dosage-dependent and -independent regulation contributes

to gene expression and evolutionary novelty in plant polyploids. Mol Biol Evol 2015;32(9): 2351-66.
Tan C, Pan Q, Cui C, Xiang Y, Ge X, Li Z. Genome-Wide Gene/Genome Dosage Imbalance Regulates Gene Expressions in Synthetic Brassica napus and Derivatives (AC, AAC, CCA, CCAA). Front Plant Sci 2016;7:1432.
Han M, Sun Q, Zhou J, Qiu H, Guo J, Lu L, et al. Insertion of a solo LTR retrotransposon associates with spur mutations in 'Red Delicious' apple (Malus × domestica). Plant Cell Rep 2017;36(9):1375-1385.
Wang P, Gu M, Yu X, Shao S, Du J, Wang Y, et al. Allele-specific expression and chromatin accessibility contribute to heterosis in tea plants (Camellia sinensis). Plant J 2023;112(5):1194-1211.
Bousios A, Diez CM, Takuno S, Bystry V, Darzentas N, Gaut BS. A role for palindromic structures in the cis-region of maize Sirevirus LTRs in transposable element evolution and host epigenetic response. Genome Res 2016;26(2):226-37.
Jordan KW, He F, de Soto MF, Akhunova A, Akhunov E. Differential chromatin accessibility landscape reveals structural and functional features of the allopolyploid wheat chromosomes. Genome Biol 2020;21(1):176.
Han J, Lopez-Arredondo D, Yu G, Wang Y, Wang B, Wall SB, et al. Genome-wide chromatin accessibility evolution during polyploidization in cotton. Proc Natl Acad Sci U S A 2022;119(44):e2209743119.
Yoo MJ, Liu X, Pires JC, Soltis PS, Soltis DE. Nonadditive gene expression in polyploids. Annu Rev Genet 2014;48: 485-517.
