# Supplementary material for: Interspecific hybridization in Brassica species leads to changes in agronomic traits through the regulation of gene expression by chromatin accessibility and DNA methylation
Source: Gigascience. 2025 Apr 22;14:giaf029. doi: 10.1093/gigascience/giaf029 (PMC12012897; doi:10.1093/gigascience/giaf029)
Supplement: giaf029_Reviewer_1_Report_Original_Submission — Zefu Lu -- 10/14/2024 [file giaf029_reviewer_1_report_original_submission.pdf]

Reviewer Report

Title: Interspecific hybridization in Brassica species leads to changes in agronomic traits through the regulation of gene expression by chromatin accessibility and DNA methylation

Version: Original Submission **Date:** 10/14/2024

Reviewer name: Zefu Lu

Reviewer Comments to Author:

The manuscript presents a wealth of data on chromatin accessibility and DNA methylation during polyploidization, offering fresh insights into these processes in the context of interspecific hybridization. While the study provides valuable observations, several key issues must be addressed to strengthen the conclusions.

Major Points:

1. Data Quality Concerns: In Fig 1B, the Hort dataset appears to be of lower quality compared to the other datasets, which may affect some conclusions. Similarly, Figure 2d shows low enrichment levels, indicating potential issues with data quality. This could impact conclusions regarding ACR distribution and differences before and after allopolyploidization. The authors should carefully reconsider the conclusions drawn, especially regarding ACR calling.
2. Read Distribution in Parent Tracks: In Fig 2G, the two parent tracks seem to have no detectable reads. The authors should verify the sequencing depth and confirm whether it is sufficient for reliable data interpretation.
3. Distribution and Shift of ACRs in the A and C Subgenomes: The authors conclude that interspecific hybridization induces changes in ACR distribution and abundance, especially in gene and proximal regions. However, it would be crucial to clarify how these changes differ between the A and C subgenomes. Are these changes biased toward one subgenome, or are they converging? The authors should compare their findings with studies such as the referenced paper on cotton hybrids (Ref. 36) and discuss the similarities and differences.

Minor Points:

1. The chromosome number diagram in Figure 1a lacks a scale bar, which should be included for reference.
2. In Fig 2F, it would be useful to show the proportions of various TE classes across the genome for better context.
3. The order of Hort distribution in Figure 2b should be made consistent for better comparison.
4. The number of additively expressed genes is mentioned, but further explanation is needed regarding how these genes were identified and their biological significance.
5. Clarify whether the enriched genes in Hybrid-yh are non-additively up-regulated, as the connection to gene regulation is unclear.
6. Replace "enrichment" with "GO enrichment" to ensure clarity in the text.
7. The section title needs revision, as much of the content focuses on gene expression rather than chromatin changes.

8. The authors should cite relevant literature supporting the impact of LTRs and LINEs on gene regulation.
9. Provide more information on the distribution of novel ACRs and their overlap with TEs. It would be important to discuss whether this overlap influences gene expression.
10. The conclusion that hybrids tend to inherit specific sequences from the parent with higher methylation needs further explanation. How was this determined?
11. The threshold used for dose-dependent and independent gene identification lacks a clear explanation. Please provide details on how these thresholds were determined.
Overall, this study is rich in data and offers valuable insights into epigenetic regulation during hybridization. However, addressing these issues will help to clarify the findings and improve the manuscript's impact.

Methods

Are the methods appropriate to the aims of the study, are they well described, and are necessary controls included? Choose an item.

Conclusions

Are the conclusions adequately supported by the data shown? Choose an item.

Reporting Standards

Does the manuscript adhere to the journal's guidelines on [minimum standards of reporting?] Choose an item.

Choose an item.

Statistics

Are you able to assess all statistics in the manuscript, including the appropriateness of statistical tests used? Choose an item.

Quality of Written English

Please indicate the quality of language in the manuscript: Choose an item.

Declaration of Competing Interests

Please complete a declaration of competing interests, considering the following questions:

- Have you in the past five years received reimbursements, fees, funding, or salary from an organisation that may in any way gain or lose financially from the publication of this manuscript, either now or in the future?

- Do you hold any stocks or shares in an organisation that may in any way gain or lose financially from the publication of this manuscript, either now or in the future?
- Do you hold or are you currently applying for any patents relating to the content of the manuscript?
- Have you received reimbursements, fees, funding, or salary from an organization that holds or has applied for patents relating to the content of the manuscript?
- Do you have any other financial competing interests?
- Do you have any non-financial competing interests in relation to this paper?

If you can answer no to all of the above, write 'I declare that I have no competing interests' below. If your reply is yes to any, please give details below.

I declare that I have no competing interests

I agree to the open peer review policy of the journal. I understand that my name will be included on my report to the authors and, if the manuscript is accepted for publication, my named report including any attachments I upload will be posted on the website along with the authors' responses. I agree for my report to be made available under an Open Access Creative Commons CC-BY license (http://creativecommons.org/licenses/by/4.0/). I understand that any comments which I do not wish to be included in my named report can be included as confidential comments to the editors, which will not be published.

Choose an item.

To further support our reviewers, we have joined with Publons, where you can gain additional credit to further highlight your hard work (see: https://publons.com/journal/530/gigascience). On publication of this paper, your review will be automatically added to Publons, you can then choose whether or not to claim your Publons credit. I understand this statement.

Yes Choose an item.
