# Supplementary material for: Interspecific hybridization in Brassica species leads to changes in agronomic traits through the regulation of gene expression by chromatin accessibility and DNA methylation
Source: Gigascience. 2025 Apr 22;14:giaf029. doi: 10.1093/gigascience/giaf029 (PMC12012897; doi:10.1093/gigascience/giaf029)
Supplement: giaf029_Reviewer_2_Report_Original_Submission — Chaobo Tong -- 12/25/2024 [file giaf029_reviewer_2_report_original_submission.pdf]

Reviewer Report

Title: Interspecific hybridization in Brassica species leads to changes in agronomic traits through the regulation of gene expression by chromatin accessibility and DNA methylation

Version: Original Submission **Date:** 12/25/2024

Reviewer name: Chaobo Tong

Reviewer Comments to Author:

This study investigates the construction of two hybrids through the crossing of two Brassica napus cultivars (s70, AsAsCsCs; yu25, AyAyCyCy, allotetraploid) with a Brassica rapa cultivar (B. campestris L. ssp. chinensis var. purpuria Hort.; AhAh, diploid). The research systematically explores accessible chromatin regions (ACRs), DNA methylation, and gene expression. Notable differences in the expression of non-additive genes and up-regulated transgressive genes associated with metal ion accumulation, as well as SPE genes linked to anthocyanin accumulation, were observed. Furthermore, the study reveals the role of ACRs and DNA methylation in regulating gene expression, including the impact of dosage effects. Before the article is accepted, the following revisions are recommended

1. The introduction is overly brief and lacks sufficient background information. It should include recent progress on accessible chromatin regions (ACRs), DNA methylation, and their role in gene regulation, especially in Brassica species or other crops. A clearer description of the research objectives and their potential implications would enhance the introduction.
2. The differential expression of non-additive genes between Hybrid-sh and Hybrid-yh is reported, with insights from GO-enrichment analysis. However, the authors should focus more on the shared gene expression patterns and provide a more in-depth discussion of how interspecies hybridization induces gene expression changes via ACRs and DNA methylation.
3. The differences in DNA methylation and ACR patterns between the two hybrids (Hybrid-sh and Hybrid-yh) induced by interspecies hybridization need to be more clearly presented in the figures and text. Additionally, the authors should offer a detailed discussion of the reasons behind these differences.
4. In the section on "Accessible Chromatin Regions and DNA Methylation Differ in Genome Dosage Effects," the authors should provide a more thorough discussion in relation to previous studies on Brassica napus or other polyploid crops. This would help contextualize the findings and demonstrate how they align with or differ from existing knowledge.
5. The discussion section currently lacks sufficient detail and does not adequately compare the results with previous studies. It would benefit from a deeper analysis of the novel insights provided by this study, as well as a discussion of any differences between the two hybrids.

Methods

Are the methods appropriate to the aims of the study, are they well described, and are necessary controls included? Choose an item.

Conclusions

Are the conclusions adequately supported by the data shown? Choose an item.

Reporting Standards

Does the manuscript adhere to the journal's guidelines on minimum standards of reporting? Choose an item.

Choose an item.

Statistics

Are you able to assess all statistics in the manuscript, including the appropriateness of statistical tests used? Choose an item.

Quality of Written English

Please indicate the quality of language in the manuscript: Choose an item.

Declaration of Competing Interests

Please complete a declaration of competing interests, considering the following questions:

- Have you in the past five years received reimbursements, fees, funding, or salary from an organisation that may in any way gain or lose financially from the publication of this manuscript, either now or in the future?
- Do you hold any stocks or shares in an organisation that may in any way gain or lose financially from the publication of this manuscript, either now or in the future?
- Do you hold or are you currently applying for any patents relating to the content of the manuscript?
- Have you received reimbursements, fees, funding, or salary from an organization that holds or has applied for patents relating to the content of the manuscript?
- Do you have any other financial competing interests?
- Do you have any non-financial competing interests in relation to this paper?

If you can answer no to all of the above, write 'I declare that I have no competing interests' below. If your reply is yes to any, please give details below.

I declare that I have no competing interests

I agree to the open peer review policy of the journal. I understand that my name will be included on my report to the authors and, if the manuscript is accepted for publication, my named report including any attachments I upload will be posted on the website along with the authors' responses. I agree for my

report to be made available under an Open Access Creative Commons CC-BY license (http://creativecommons.org/licenses/by/4.0/). I understand that any comments which I do not wish to be included in my named report can be included as confidential comments to the editors, which will not be published.

Choose an item.

To further support our reviewers, we have joined with Publons, where you can gain additional credit to further highlight your hard work (see: https://publons.com/journal/530/gigascience). On publication of this paper, your review will be automatically added to Publons, you can then choose whether or not to claim your Publons credit. I understand this statement.

Yes Choose an item.
